# Mucosal‐Associated Invariant T Cells: Origins, Biological Functions, Diseases, and Therapeutic Targets

**DOI:** 10.1002/mco2.70445

**Published:** 2025-11-01

**Authors:** Cheng Zhu, Qian Huai, Yishan Du, Xingyu Li, Fumin Zhang, Yongkang Zhang, Mengwei Wu, Ying Dai, Xiaolei Li, Hanren Dai, Hua Wang

**Affiliations:** ^1^ Department of Oncology The First Affiliated Hospital of Anhui Medical University Hefei China; ^2^ Key Laboratory of Anti‐Inflammatory and Immune Medicine (Anhui Medical University) Ministry of Education Hefei China; ^3^ Department of Geriatrics Division of Life Sciences and Medicine The First Affiliated Hospital of USTC University of Science and Technology of China Hefei China; ^4^ Department of Gastroenterology The First Affiliated Hospital of Anhui Medical University Hefei China; ^5^ The Second Department of Critical Care Medicine The Second Affiliated Hospital of Anhui Medical University MOE Key Laboratory of Anti‐Inflammatory and Immune Medicine and Institute of Clinical Pharmacology Anhui Medical University Hefei China

**Keywords:** autoimmune disease, cancer, immunotherapy, infectious disease, MAIT cells, MR1, unconventional T cells

## Abstract

Mucosal‐associated invariant T (MAIT) cells are a highly conserved population of immune cells that can be activated via the major histocompatibility complex class I‐related protein pathway or cytokine pathways, playing a central role in immune surveillance. This review provides comprehensive information on their thymic developmental origin, tissue‐specific distribution, and microbial regulatory networks, with a focus on analyzing the bidirectional regulatory mechanisms in diseases. In infectious diseases, MAIT cells eliminate pathogens through the rapid release of cytokines; however, sustained antigen exposure leads to functional exhaustion. In autoimmune diseases, their migration disorders and proinflammatory cytokine secretion of MAIT cells exacerbate tissue damage. In the tumor microenvironment, they play a paradoxical role, being capable of mediating antitumor effects while also being reprogrammed into a protumor phenotype. Based on their tissue targeting ability and functional plasticity, we discuss novel strategies for targeted therapy, including engineering chimeric antigen receptor–MAIT cells to enhance tumor killing, blocking exhaustion pathways to reverse functional impairment, and regulating the microbiota–metabolic axis to reprogram cell activity. This review integrates cutting‐edge evidence, reveals the translational potential of MAIT cells as a cross‐disease regulatory hub, and provides a theoretical framework for precision immunotherapy.

## Graphical abstract

Mucosa‐associated invariant T (MAIT) cells are an evolutionarily conserved class of innate‐like T lymphocytes that rely on MR1 molecules to recognize microbial metabolites and play a central role in immune surveillance and inflammatory regulation. In this paper, we systematically integrated the whole process of MAIT cells from thymic development, peripheral tissue settlement to microbial–metabolic microenvironment regulation, focusing on revealing their dual functional roles in three major categories of infectious diseases, autoimmune disorders, and tumors: both early protective effects and mediation of pathological damage. The cutting‐edge therapeutic strategies targeting MAIT cells, including CAR–MAIT cell engineering design, depletion function reversal, and microbial–metabolic axis reprogramming, are further summarized to provide theoretical basis and translational perspectives for immunotherapy across disease modalities.

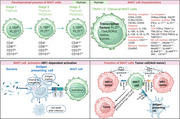

## 1 Introduction

Mucosal‐associated invariant T (MAIT) cells are an evolutionarily conserved subset of innate‐like T lymphocytes. Their development depends on major histocompatibility complex (MHC)‐Ib‐related protein 1 (MR1)‐mediated positive selection in the thymus. They recognize microbial vitamin B2 (riboflavin) metabolic derivatives through a semi‐invariant T‐cell receptor (TCR Vα7.2–Jα33) and play a central role in host defense, tissue repair, and immune regulation [1]. Although relatively rare in peripheral circulation, MAIT cells play an irreplaceable hub role in bridging innate and adaptive immune responses by releasing interferon‐gamma (IFN‐γ), tumor necrosis factor α (TNF‐α), and cytotoxic molecules. Their functional status is continuously shaped by the dynamic changes in microbial communities, tissue microenvironment, and metabolites [2].

In recent years, studies have revealed that MAIT cells exhibit complex functional heterogeneity and paradoxical biological behaviors in disease progression. In infectious diseases, these cells show dual characteristics: they can exert a strong protective effect in the early stages of infection, but may undergo progressive functional and population decline under sustained antigen exposure [3, 4]. In autoimmune diseases, MAIT cells can maintain the integrity of the intestinal epithelial barrier by secreting interleukin (IL)‐22 and can also transform into a major source of the proinflammatory factor IL‐17A under pathological conditions [5, 6]. In the tumor microenvironment (TME), MAIT cells can bind to the antigen‐presenting molecule MR1 on tumor cells, thereby exerting antitumor effects. This specific recognition mechanism enables MAIT cells to launch immune attacks against tumor‐specific antigens presented by MR1, thereby inhibiting tumor growth and spread [7]. In addition, MAIT cells can secrete a variety of cytokines, such as TNF, IFN‐γ, perforin, and granzyme B (GZMB), to further activate immune responses and enhance antitumor effects [1, 8]. In terms of protumor effects, MAIT cells can exhibit immune functions similar to those of Th17 cells. The IL‐17 secreted by MAIT cells can promote cancer progression through multiple mechanisms, such as upregulating the expression of vascular endothelial growth factor (VEGF) and VEGF receptors, promoting the recruitment and expansion of immunosuppressive cell populations in tumors, and participating in immune escape and drug resistance of tumor cells [9, 10, 11, 12]. However, research on these aspects currently faces multidimensional bottlenecks: a lack of systematic analysis on how tissue microenvironment‐specific signals dynamically shape the functional phenotype of MAIT cells; lack of clarity regarding key molecular switches that drive their transformation from a protective to a pathological nature; targeted therapy strategies facing challenges such as insufficient in vivo persistence, difficulty in tissue‐specific delivery, and safety risks.

Starting from the origin and developmental trajectory of MAIT cells, this article systematically elaborates on the molecular mechanisms of their biological functions; analyzes paradoxes regarding their role in infectious diseases, autoimmune diseases, and tumors; and discusses the translational prospects and challenges of MAIT cells as new therapeutic targets in combination with recent research findings. By integrating basic research and clinical evidence, we expected to provide new ideas for the prevention and treatment of MAIT cell‐related diseases.

## 2 Developmental Origin and Biological Properties of MAIT Cells

As an unconventional T cell subpopulation that has attracted much attention in the field of immunology, MAIT cells have made key breakthroughs in the study of their functional properties and developmental mechanisms in recent years. In this section, we systematically describe the biological properties of MAIT cells, including their unique recognition mechanism of microbial riboflavin metabolites presented by MR1 molecules via semi‐constant TCRs, and their distribution patterns in different tissues. Focusing on the developmental trajectory of MAIT cells within the thymus and their species‐specific differences, we also resolve their dual activation pathways through TCR‐dependent and cytokine‐mediated activation. Finally, the understanding of the unique localization of MAIT cells in the immune system is deepened by comparing the recognition mechanisms and functional properties of other unconventional T cells (NKT, γδT).

### 2.1 The Discovery and Phenotype of MAIT Cells

#### 2.1.1 MAIT Cells: A Brief History

MAIT cells were initially discovered over 30 years ago after identification of a population of αβ T cells enriched in the double‐negative (CD4^−^CD8^−^) subset expressing an invariant Vα7.2–Jα33 TCR [13]. A T cell subset expressing a homologous TCR‐α chain was then identified in mice and cattle [14], indicating evolutionary conservatism and functional importance. MAIT cells are restricted to the nonpolymorphic MHC class I‐like protein MR1, a β2‐microglobulin‐associated antigen‐presenting molecule. Subsequent studies established the term MAIT cell, due to the relative enrichment of these T cells within mucosal tissues [15]. MAIT cell development is a stepwise process, with an intrathymic selection followed by peripheral expansion [16]. Two studies from 2010 demonstrated that MAIT cells respond to a wide range of bacteria and yeasts, but that viruses were nonstimulatory [17, 18]. In 2012, a breakthrough was made revealing that the activation ligand for MAIT cells is a precursor derivative of vitamin B2, a molecule only found in bacteria and yeast capable of synthesizing riboflavin [19]. Further work in 2014 clarified that the potent stimulatory ligand in the riboflavin synthesis pathway was a nonenzymatic derivative of 5‐A‐RU (5‐amino‐6‐d‐ribitylaminouracil) [20]. Subsequently, the identification of vitamin B2/B9 precursors as ligands for MR1 allowed for the rapid development of MR1 tetramers in mice and humans [21, 22]. Researchers have currently begun to investigate the mechanisms of MAIT cells in immune regulation and disease defense, while also strengthening research on their clinical applications, providing more precise strategies and methods for immunotherapy and vaccine design [23, 24, 25, 26, 27]. The next stage of research will focus on elucidating the relationship between MAIT cells and the development of various diseases, providing new ideas and directions for disease prevention and treatment.

#### 2.1.2 Distribution of MAIT Cells

**FIGURE 1:**
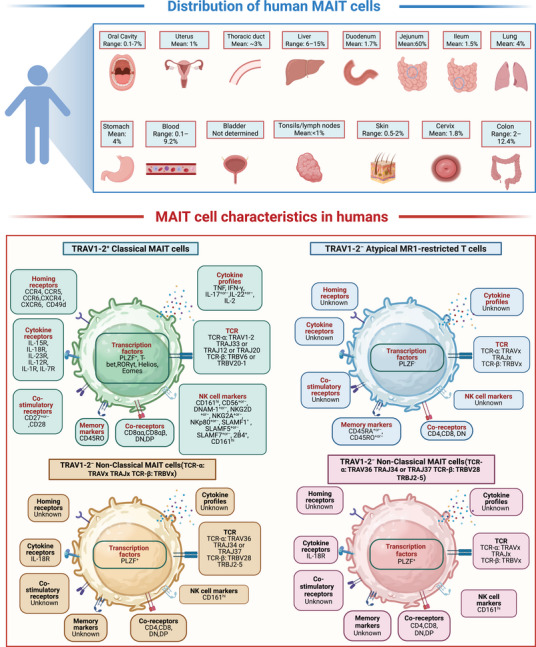
Distribution and characteristics of MAIT cells in human. Grouping of MAIT cells based on the definition proposed by Godfrey et al. [1, 369]. Phenotype of human MAIT cells: TRAV1‐2^+^ classical MAIT cells, TRAV1‐2^−^ atypical MR1‐restricted T cells, TRAV1‐2^−^ nonclassical MAIT cells (TCR‐α: TRAVx TRAJx, TCR‐β: TRBVx), TRAV1‐2^−^ nonclassical MAIT cells (TCR‐α: TRAV36 TRAJ34 or TRAJ37, TCR‐β: TRBV28 TRBJ2‐5). The picture is created in https://www.biorender.com/.

**FIGURE 2:**
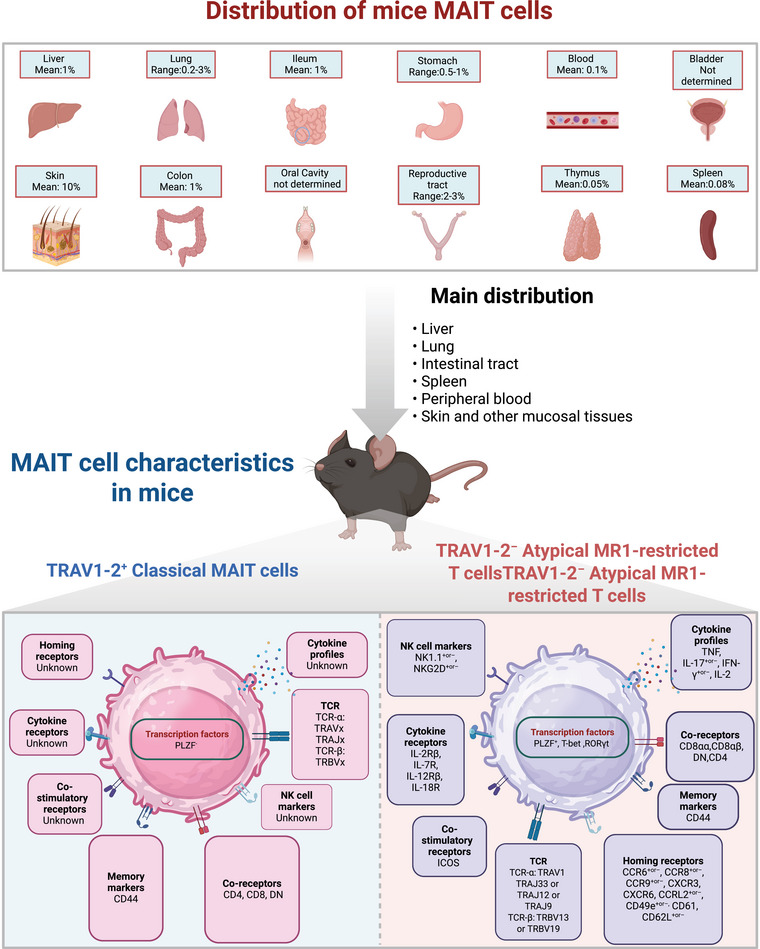
Distribution and characteristics of MAIT cells in mice. Grouping of MAIT cells based on the definition proposed by Godfrey et al. [1, 369]. Phenotype of mouse MAIT cells: TRAV1‐2^+^ classical MAIT cells, TRAV1‐2^−^ atypical R1‐restricted T cells. The picture is created in https://www.biorender.com/.

MAIT cells are an important subset of human T cells, accounting for 1–35% of T cells, and are widely distributed throughout. MAIT cells are enriched in the intestine (most abundant in the jejunum, about 60% of CD4^−^ T cells), liver (20–50% of T cells), and lungs (2–4% of T cells), and are also found in small quantities in the kidneys, lymphoid organs (tonsils and lymph nodes), ovaries, prostate, adipose tissue, and skin [22, 28, 29, 30, 31, 32, 33, 34, 35, 36, 37, 38, 39, 40, 41, 42]. In peripheral blood, MAIT cells account for 1–10% of total T lymphocytes, with their proportion peaking in adulthood and declining with age. Additionally, the number of MAIT cells in women of childbearing age is significantly higher than in age‐matched males [43]. MAIT cells are relatively conserved in evolution between mice and humans, but their frequencies are significantly different. They account for only 0.05, 0.08, and 1% in the thymus, spleen, and liver of mice, respectively (Figures 1 and 2) [14, 21, 44, 45].

#### 2.1.3 Phenotype of MAIT Cells

MAIT cells have three subtypes, mainly CD8^+^ T cells (70–80%), followed by CD4^−^CD8^−^ T cells (10–20%), with a minority being CD4^+^ T cells [46]. In detail, MAIT cell can also exhibit an effector memory phenotype (CD45RA^−^CD45RO^+^CD95^hi^CD62L^lo^), CCR7^−^CCR9^int^ CCR5^hi^ CCR6^hi^ CXCR6^hi^ (in human peripheral blood) [33]. The phenotype of MAIT cells is characterized by the expression of specific cell surface markers, including semi‐invariant TCR and chemokine receptors. The unique TCR of MAIT cells consists of a constant α chain and a variable β chain. In humans, the α chain of MAIT cells mainly includes Vα7.2–Jα33 (with some variant subtypes like Vα7.2–Jα20), where Vα7.2 is a variable segment and Jα33 is a joining segment. Additionally, the variable β chain typically includes Vβ2 and Vβ13. In mice, the constant α chain of MAIT cell TCR mainly consists of Vα19–Jα33. Correspondingly, the variable β chain includes Vβ6, Vβ8.1, and Vβ8.2 [1, 15, 38].

MAIT cells express a diverse array of tissue‐homing chemokine receptors, such as CCR6, CXCR6, CCR5, CCR2, and α4β7, reflecting their homing abilities to different anatomical sites. For example, the high levels of CXCR6 expression by MAIT cells lead to their high frequency in the liver [33, 47, 48, 49]. Expression of CCR6 promotes migration to mucosal surfaces and responses to bacteria and fungi [50]. In addition, MAIT cells also express various natural killer (NK) receptors on their surface, such as CD161, NKG2D, NKP30, and NKp80 [34, 51].

In addition, MAIT cells also express various transcription factors, such as T‐bet, RORγt, PLZF, STAT3, EOMES, and Blimp‐1. As well as orchestrating MAIT cell development, PLZF is known to regulate the ability of invariant NK T (iNKT) cells, Vδ2^+^ γδ T cells, and NK cells to be activated by cytokines [52, 53, 54]. Meanwhile, RORγt and STAT3 are involved in the differentiation program of MAIT cells toward the Th17 type, controlling their production of IL‐17 and IL‐22 [48, 55, 56]. The expression of T‐bet, EOMES, and Blimp‐1 endows MAIT cells with characteristics of conventional CD8 T cells, including the production of IFN‐γ and cytotoxic granules [57, 58, 59].

### 2.2 Development and Activation of MAIT Cells

#### 2.2.1 Development of MAIT Cells

MAIT cells are derived from the thymus and undergo a developmental pathway akin to conventional T cells, involving positive and negative selection within the thymic cortex. Throughout this process, the TCR and other immune‐related receptors of MAIT cells are subject to appropriate selection and regulation. It is worth noting that the development process of MAIT cells in mice differs from that in humans.

In mice, the development of MAIT cells is activated by recognizing and responding to metabolites synthesized by microorganisms. MAIT cells undergo three developmental stages in the thymus: the stage 1 is defined as CD24^+^CD44^−^, the stage 2 is CD24^−^CD44^−^, and the stage 3 is CD24^−^CD44^+^ [60].

In humans, the development process of MAIT cells does not initially occur through direct activation by bacteria, but is related to early immune education and differentiation. Three similar stages of MAIT cell development can be identified in human body, albeit through the use of different cell‐surface markers. The corresponding stages are defined as CD27^−^CD161^−^, CD27^+^CD161^−^, and CD27^+^CD161^+^ cells [60].

**FIGURE 3:**
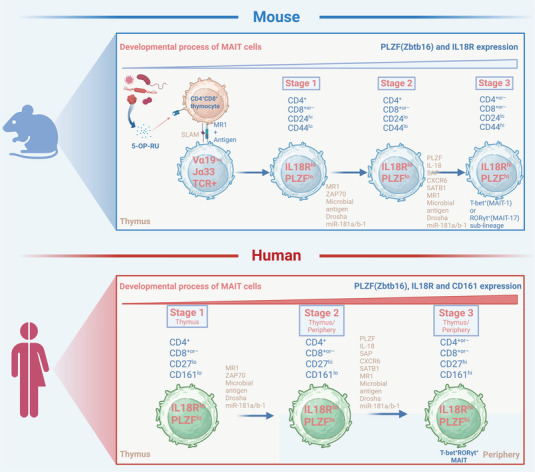
Development stages of MAIT cells. Top, development process of MAIT cells in mice. Bottom, development process of MAIT cells in humans. Mouse stage 1 (CD24⁺CD44^−^) is driven by microbial metabolites, and after stage 2 (CD24^−^CD44^−^), the functional differentiation of IFN‐γ⁺MAIT1 and IL‐17⁺MAIT17 is completed in the thymus during stage 3 (CD24^−^CD44⁺). In humans, the process begins in a germ‐free environment, where CD27^−^CD161^−^ precursors are selected by the thymic epithelial cell MR1‐riboflavin ligand, sequentially transitioning to CD27⁺CD161^−^ and CD27⁺CD161⁺, and ultimately maturing in the periphery. Human stage 2/3 cells can be detected simultaneously in the thymus and blood, whereas mouse stage 3 cells are exclusively present in the thymus. The picture is created in https://www.biorender.com/.

During the development process, interaction with MR1 is required at each stage, along with distinct cofactors. The progression from stage 1 to stage 2 also depends on Drosha (the microRNA processing enzyme), miR‐181a/b‐1, and the presence of undefined microbial factors because this process is impaired in germ‐free mice [60, 61]. In addition, signaling through SLAM‐associated protein (SAP) also appears crucial for the development of mouse MAIT cells beyond stage 1, but its role in human MAIT cell development is unclear, as the normal numbers of peripheral MAIT cells observed in patients with SAP deficiency [62, 63]. Transition from stage 2 to 3 represents key events in MAIT cell maturation where MAIT cells not only enter their final maturation stage but also diversify into functionally distinct subsets, which is regulated by multiple factors, including PLZF, IL‐18, and undefined microbial factors [60]. In mice, MAIT cells differentiate into functionally distinct MAIT1 (T‐bet⁺ RORγt^−^) and MAIT17 (T‐bet^−^ RORγt⁺) subpopulations during thymic development and maintain stable phenotypic characteristics [21, 60]. In stark contrast, human MAIT cells typically exhibit a mixed MAIT1/17 phenotype, primarily due to their universal coexpression of the T‐bet and RORγt transcription factors. This coexpression pattern is particularly pronounced in human peripheral blood MAIT cells, enabling them to flexibly produce effector molecules such as IFN‐γ or IL‐17A in response to microenvironmental stimuli, reflecting functional plasticity [60, 64]. Notably, despite this mixed phenotypic background, MAIT cells in human tissues exhibit functional bias. Circulating MAIT cells primarily exhibit IFN‐γ‐dominant MAIT1‐like responses, while subpopulations in mucosal or inflammatory tissues tend toward IL‐17A secretion, suggesting that local microenvironments can regulate their functional polarization (Figure 3) [65].

Despite some differences between mouse and human are observed, a deep understanding of the developmental process of MAIT cells in mice remains important for studying the development and function of human MAIT cells.

#### 2.2.2 Activation of MAIT Cells

MAIT cells could be activated through two distinct manners, including TCR‐dependent manner by antigens derived from microbe or TCR‐independent activation by cytokines [1, 19, 21, 27, 48, 49, 51, 66, 67, 68, 69, 70, 71, 72, 73, 74, 75, 76, 77, 78, 79, 80, 81]. Tellingly, TCR‐dependent and TCR‐independent signals drive MAIT cells to exert overlapping and specific effector functions, affecting both host defense and tissue homeostasis [34, 49, 68, 75, 76, 82, 83, 84, 85, 86, 87].

Riboflavin, which is produced by most microorganisms, could activate MAIT cells through a TCR‐dependent pathway [17, 18, 68, 88, 89]. Studies have found that the intermediate metabolites of the riboflavin synthesis pathway, such as 5‐A‐RU, 5‐(2‐oxopropylideneamino)‐6‐d‐RU (5‐OP‐RU), and 5‐(2‐oxoethylideneamino)‐6‐d‐RU (5‐OE‐RU), can bind to MR1, thereby strongly activating MAIT cells through a TCR‐dependent pathway [20, 90, 91]. In contrast, 6‐formylpterin (6‐FP) and acetyl‐6‐FP (Ac‐6‐FP) can competitively bind to MR1, thereby inhibiting the activation of MAIT cells [19, 90, 92, 93]. Research has also found that antigen‐presenting cells (APCs), upon capturing intact bacteria rather than soluble ligands entering the lysosomal lumen, upregulate MR1 through the nuclear factor κB (NF‐κB) protein or IFN signaling pathways, thereby activating MAIT cells through a TCR‐dependent pathway [94].

Upon activation, MAIT cells produce different cytokines and mediators, depending on the mode of activation, the characteristics of the stimulating factors, and the postactivation microenvironment. In human, following recognition of VB2‐type antigens presented by MR1, the expression of CD25, CD69, and CD161 is elevated [95, 96]. MAIT cells from human tissues such as the liver and female reproductive tract make large amounts of IL‐17A and IL‐22 after activation. However, activated human blood MAIT cells secrete predominantly IFN‐γ and TNF, and only a minor population produces IL‐17A [33, 34, 42]. The major population of MAIT cells in mice expresses RORγt, which secretes IL‐17 upon activation, while a smaller subset expresses T‐bet, which produces IFN‐γ upon activation. Accordingly, mouse MAIT cells exhibit a CD44^hi^CD62L^lo^ memory phenotype and produce high levels of IL‐17A, whereas other cytokines, such as IFN‐γ, IL‐4, IL‐10, IL‐13, and granulocyte–macrophage colony‐stimulating factor (GM‐CSF), are produced at low to moderate levels [21, 45]. Investigations into the secretion of factors by MAIT cells in mice and humans are warranted.

Without TCR‐mediated antigen recognition, MAIT cells can be partially activated by cytokines, foregoing the requirement for detection of microbial antigens and allowing responses to other inflammatory stimuli such as viral infections [68, 72, 86, 97, 98, 99, 100, 101, 102]. Accordingly, MAIT cells express high levels of innate cytokine receptors, such as IL‐1R, IL‐12R, IL‐18R, and IL‐23R, and can respond to these cytokines in the absence of TCR ligation. Tellingly, IL‐12 and IL‐18 have been well studies in TCR‐independent activation of MAIT cells [72]. In addition, IL‐15 can specifically activate distinct functions of MAIT cells in synergy with IL‐12 and/or IL‐18. MAIT cells could also be activated by IL‐23, which shares the receptor chain IL‐12Rβ1 with IL‐12 [103]. Notably, IL‐23 costimulates antigen‐specific MAIT cell activation and enables vaccination against bacterial infection. The type I IFNs IFN‐α or IFN‐β and the gut‐associated proinflammatory cytokine, TNF‐like protein 1A (TL1A/TNFSF15) can cooperate with IL‐18 and/or IL‐12 to activate MAIT cells [72, 104, 105, 106, 107]. Cytokine‐dependent activation requires a combination of at least two cytokines, but either alone does not [72, 76]. Toll‐like receptor ligands can also activate MAIT cells via induction of activating cytokines [68, 76]. Interestingly, superantigens (SAg) are microbe‐produced toxins that bind both class II MHC molecules and TCR, causing activation of the T cell. MAIT cells are major responders to microbial infection and SAgs. Noticeably, MAIT cells can be activated not only directly by binding of the SAg to its TCR‐Vβ region, but also indirectly by releasing IL‐12 and IL‐18 from the SAg‐activated T cells [108, 109, 110]. Moreover, activated human MAIT cells are able to instruct dendritic cell (DC) maturation through the MR1 and CD40L‐dependent pathway, resulting in secretion of IL‐12, indicating that MAIT cells can also promote bystander activation of other cells [111].

**FIGURE 4:**
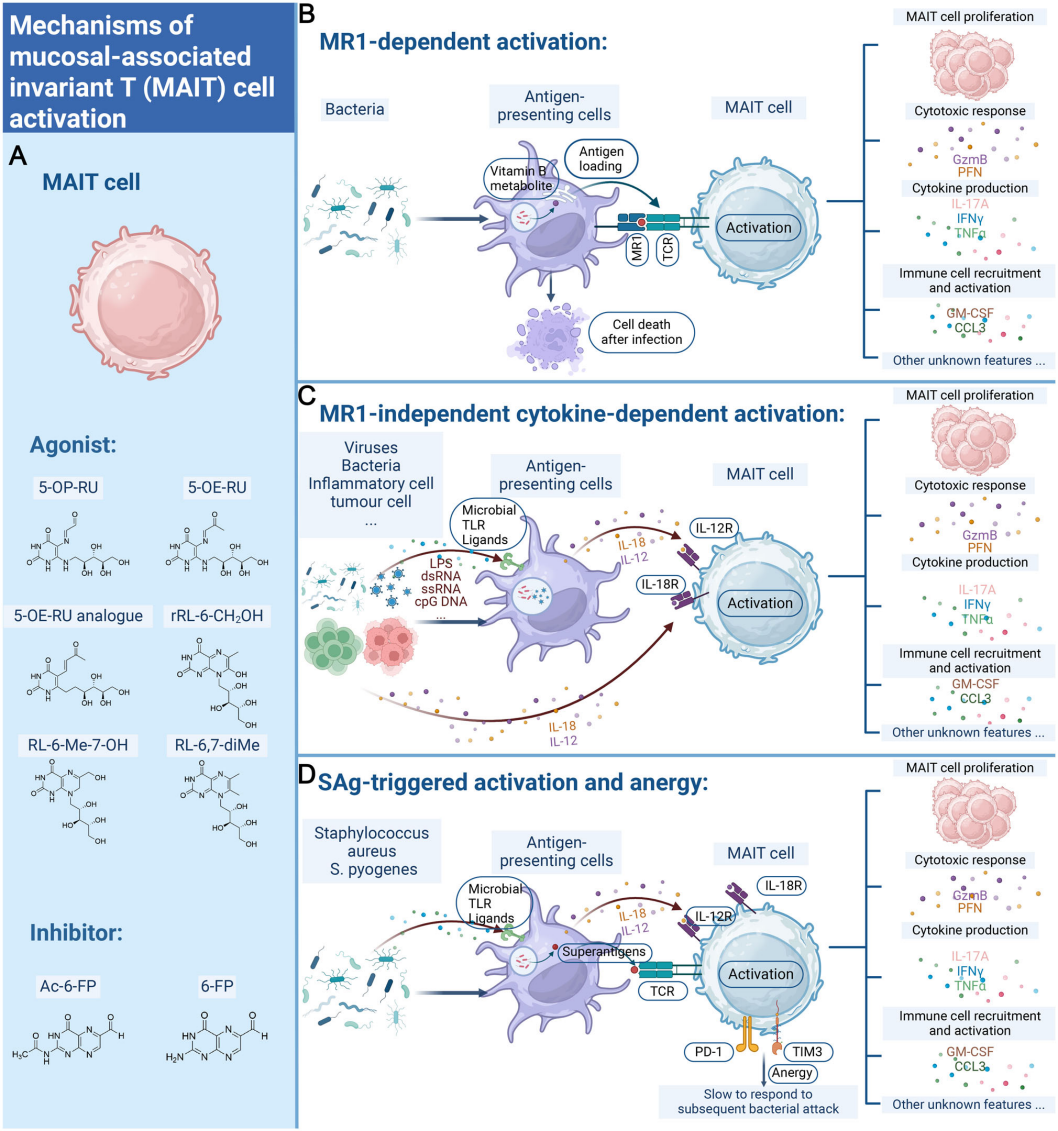
Activation process of MAIT cells. (A) Activators and inhibitors of MAIT cells. (B) MR1‐dependent activation. (C) MR1‐independent cytokine‐dependent activation. (D) SAg‐triggered activation and anergy. The picture is created in https://www.biorender.com/.

TCR‐ and/or cytokine‐activated MAIT cells could exert overlapping and specific effector functions, affecting both host defense and tissue homeostasis. Two activation modes may act in concert or independently, depending upon the stimulus. Noteworthy, 5‐OP‐RU plus additional TLR agonists causes higher levels of activation as well as proliferation of the MAIT cell pool. Moreover, IL‐12 and IL‐18, in synergy with TCR triggering, promote the activation of MAIT cells [68, 86]. Currently, besides IL‐12, IL‐15, and IL‐18 which can activate independently of TCR, most cytokines (such as IL‐1β, IL‐7, and TNF‐α) act synergistically with TCR signaling [34, 85, 86, 87]. This diversity of activation pathways provides flexibility to the immune system and plays a crucial role in responding to various pathogens and environmental stresses (Figure 4).

### 2.3 Atypical T Cells: MAIT, NKT, γδT

Unconventional T cells, as a unique subset of T lymphocytes, possess the characteristic of being activated without relying on antigen presentation by classical MHC molecules. They play an irreplaceable role in initiating immune responses, protecting mucosal barriers, and regulating diseases (Table 1). Among them, iNKT cells, γδT cells, and MAIT cells are important members of this family [112]. As heterogeneous populations connecting innate immunity and adaptive immunity, they can quickly sense and respond to microenvironmental changes related to infections and tumors and play a key role in immune responses [113]. In terms of tissue distribution, MAIT cells are enriched in the liver (accounting for 20–40% of liver T cells) and the lamina propria of the intestine; iNKT cells are mainly resident in the liver and adipose tissue; γδT cells are densely distributed in the epidermal layer of the skin, intestinal epithelium, and genital mucosa. Studies have shown that most unconventional T cells can develop in the thymus, but their differentiation trajectories are significantly different. MAIT cells and iNKT cells share a mechanism of positive selection that depends on the MR1/CD1d molecules expressed by thymic epithelial cells. The semi‐invariant TCR (Vα24–Jα18/Vβ11) of iNKT cells can specifically recognize glycolipid antigens (such as α‐GalCer) presented by CD1d; while MAIT cells bind microbial riboflavin metabolites (5‐OP‐RU) delivered by MR1 through Vα7.2–Jα33 TCR. The differentiation of γδT cells shows significant heterogeneity. Their TCR is formed by the free combination of Vγ9/Vδ2 or Vδ1 chains, which can directly sense phosphorylated antigens (such as HMBPP) or stress molecules (such as MICA/B) without the participation of APCs [112]. These unique antigen recognition properties enable unconventional T cells to broadly respond to pathogenic invasions and cellular stress (such as abnormal metabolism of tumor cells), thereby demonstrating significant advantages in tumor surveillance and elimination—they can directly initiate immune responses without the need for complex antigen processing. In terms of the requirements for activation signals, different types of unconventional T cells also have their own characteristics. The activation of MAIT cells is “dependent on dual signals.” It requires both the antigen signal presented by MR1 and costimulatory signals from cytokines such as IL‐12 and IL‐18, with the antigen signal being a necessary prerequisite for initiating activation. This strict signal requirement endows their activation with a strong targeting ability toward microbial infections. The activation of iNKT cells, on the other hand, exhibits “signal flexibility.” They can be activated either solely through the antigen signal from CD1d–glycolipid complexes or directly by cytokines like IL‐12 and IL‐18 in the absence of antigens. This dual activation mode, which is both “antigen‐dependent and antigen‐independent,” allows them to cope with specific infections and participate in nonspecific inflammatory responses. γδT cells have the most lenient requirements for activation signals; they can initiate effector functions with only a single signal (such as phosphorylated antigens or IL‐2) and are not prone to functional exhaustion after activation. This characteristic enables them to maintain long‐term immune activity in chronic infections and TMEs [114]. Based on differences in their biological characteristics, the three types of cells have shown differentiated development paths in therapeutic development. MAIT cells possess MR1‐restricted tumor recognition ability; for instance, acute myeloid leukemia (AML) cells highly express MR1, making them a new target for chimeric antigen receptor (CAR)–MAIT therapy—preclinical models have shown that the tumor clearance rate can reach 60%. Meanwhile, the metabolic dependence of MAIT cells also indicates their value in the design of antituberculosis vaccines; for example, the 5‐OP‐RU adjuvant can enhance immune responses. However, overall, clinical applications based on MAIT cells are still in the developmental stage [23, 24]. iNKT cells have the property of activating DCs to enhance tumor antigen presentation, which makes them highly effective in DC vaccines pulsed with α‐GalCer. In addition, multiple clinical trials have confirmed the antitumor effects of CAR–iNKT in multiple myeloma (MM) and neuroblastoma [115, 116]. Due to their MHC‐nonrestrictive killing property, γδ T cells have made rapid progress in CAR–γδ T therapy, showing significant efficacy especially in allogeneic treatments for hematological malignancies such as MM and Hodgkin's lymphoma. Beyond CAR engineering, these cells are further modified to coexpress IL‐2, thereby extending their antitumor activity [117, 118, 119]. However, these three types of cells face common challenges in their applications that urgently need to be addressed: first, insufficient in vivo persistence; second, being affected by the suppressive microenvironment; third, the presence of terminal exhaustion. In summary, unconventional T cells with unique biological characteristics are expected to become a new frontier in future immunotherapy, and their basic research and clinical translation in the field of immunology deserve further in‐depth exploration.

**TABLE 1:** Classification and applications of unconventional T cells.

Unconventional T cell types	Subtypes	marker	Recognized molecules	Antigen recognition	Tissue distribution	Abundance in blood	Function	Application in disease therapy	NCT	References
γδT cell	Vδ1, Vδ2, Vδ3, Vδ5	γδ TCR, CD3	CD1d	HMBPP, IPP	Intestine, lung	Around 1–10%	Bridging innate and adaptive immunity while mounting a rapid response to infections	B7‐H3‐targeting CAR–γδT cells carrying IL‐2 in myeloid leukemia CD19‐targeting CAR–γδT cells in B cell malignancies NKG2DL‐targeting CAR–γδT cells in solid tumor NKG2DL‐targeting CAR–γδT cells in hematological malignancies BCMA‐targeting CAR–γδT cells in multiple myeloma CD20‐targeting CAR–γδT cells in diffuse large B cell lymphoma, follicular lymphoma CD123‐targeting CAR–γδT cells in acute myeloid leukemia HLA‐G‐targeting CAR–γδT cells in triple negative breast cancer, non‐small cell lung cancer, or glioblastoma CAR–γδ T cells with flavivirus antigen in flavivirus infections	NCT04796441 NCT02656147 NCT04107142 NCT05302037 NCT06279026 NCT04911478 NCT04796441 NCT05388305 NCT06150885	[120]
NKT cell	Type I NKT (iNKT), type II NKT cells	TCR, CD1d	CD1d	glycolipid antigens	Adipose tissue, liver, lung, intestine	Around 0.001–1%	Detecting lipid antigens and exhibiting characteristics of both NK cells and T cells	GD2‐targeting CAR–iNKT in neuroblastoma CD19‐targeting CAR–iNKT in B cell lymphoma CD19‐targeting CAR–iNKT in lymphoma CD70‐targeting CAR–iNKT in solid cancer	NCT03294954 NCT05487651 NCT03774654 NCT06728189 NCT06394622	—
MAIT cell	—	TCR, MR1	MR1	intermediates of riboflavin biosynthesis	lung, gastrointestinal tract, colon	Around 1–10%	Recognizing microbial metabolites and protecting mucosal surfaces	Her2‐targeting CAR–MAIT in breast cancer CD19 targeting CAR–MAIT in B cell lymphoma	—	[24]

National Clinical Trial (NCT) data sources: ClinicalTrials.gov.

## 3 Functional Characteristics and Roles of MAIT Cells in Anti‐Infective Immunity

MAIT cells are a key subset of innate‐like T cells that play key roles in anti‐infective immunity. Owing to their dual activation mode—antigen recognition mediated by MR1 and nonspecific activation driven by cytokines—along with characteristics such as rapid response and wide distribution, MAIT cells can combat infections caused by various pathogens such as bacteria, viruses, and fungi. In recent years, studies have revealed the different functions of MAIT cells during different types of infections. During bacterial infections, MAIT cells can either directly clear pathogens or undergo functional exhaustion; during viral infections, their response of MAIT cells varies with infection stage and viral type; their role in antifungal immunity is regulated by multiple factors; additionally, their role during parasitic and rickettsial infections has also begun to be elucidated. The following sections will provide details regarding the mechanism of action, dynamic characteristics, and clinical significance of MAIT cells during different infections, demonstrating their central position in anti‐infective immunity and the complexity of their regulation.

### 3.1 Bacteria

MAIT cells can recognize microbe‐derived vitamin B_2_ metabolic derivatives (e.g., antigens produced by certain gram‐negative bacteria such as *Escherichia coli*, *Klebsiella pneumoniae*, and *Shigella flexneri*) presented by the MHC class I‐related molecule MR1 through the MR1‐dependent pathway. Activation of this pathway mediates cytotoxic effects against infected cells [1, 121, 122]. On the contrary, the MR1‐independent pathway is triggered by IL‐12 and IL‐18 produced by APCs via the TLR8 signaling pathway following bacterial exposure, a process that does not rely on the riboflavin synthesis capacity of bacteria [123, 124, 125, 126]. Temporal dynamic studies have shown that during *E. coli* infection, early activation is primarily MR1 dependent, whereas late‐stage activation requires the combination of signals from MR1 and IL‐12/IL‐18 [127, 128].

Activated MAIT cells exert antibacterial functions through cytokine secretion. For instance, under IgG opsonization, the secretion of IFN‐γ secretion is significantly enhanced during *E. coli* infection [129, 130]; during *Salmonella typhi* infection, IFN‐γ acts synergistically with TNF‐α and IL‐17A to inhibit bacterial proliferation, enhance macrophage activity, and recruit immune cells [131]; in the presence of IL‐15, MAIT cells exhibit a strengthened IFN‐γ response to *Mycobacterium tuberculosis* lysates [125]. Additionally, MAIT cells display direct cytotoxicity, such as directly killing *E. coli* via granulysin [127] and lysing *S. flexneri*‐infected epithelial cells [129].

MAIT cells exhibit significant phenotypic plasticity during bacterial infections. In mouse models, RORγt⁺ MAIT17 cells can differentiate into RORγt⁺ T‐bet⁺ MAIT1/17 or RORγt^−^ T‐bet⁺ MAIT1 cells following infection with *Legionella* or *Francisella tularensis* to adapt to mucosal immune demands [2, 132]. Their migration dynamics also possess distinctive features. In patients with active tuberculosis (TB) [125], cystic fibrosis complicated by *Pseudomonas aeruginosa* infection [131], *S. typhi* infection [133], *Vibrio cholerae* infection [134], and septic shock [135, 136], the number of MAIT cells in peripheral blood is significantly reduced due to apoptosis and migration [137, 138], sputum and bronchoalveolar lavage fluid from patients with community‐acquired pneumonia [139, 140] and most of these cells are in an activated state. MAIT cells primarily play a protective role during infections. Clinical studies have shown that patients with sepsis with a higher frequency of circulating MAIT cells have a better prognosis [135]. MR1‐deficient mice (which lack MAIT cells) exhibit increased splenic bacterial loads after *E. coli* infection [121, 141], enhanced susceptibility to *K. pneumoniae*, and high mortality owing to disseminated infection [141]. Mice with MAIT cell expansion show an improved ability to control *E. coli* and *M. tuberculosis* infection [123]. MAIT cells can also mediate protection against *Legionella longbeachae* pulmonary infection via IFN‐γ and GM‐CSF and promote protective immune responses in sepsis [132]. Contrarily, abnormal activation of MAIT cells may also mediate pathological damage; for example, in chronic *Helicobacter pylori* infection, activation of gastric MAIT cells can exacerbate gastritis and gastric atrophy [128, 142, 143].

Certain pathogens, such as *Staphylococcus aureus*, can disrupt the normal function of MAIT cells via a SAg strategy. SAgs abnormally activate MAIT cells through a dual mechanism: first, direct binding via MHC class II molecules and the TCR Vβ region; second, amplification via an IL‐12/IL‐18‐mediated cytokine storm. This activation causes MAIT cells to release large amounts of proinflammatory cytokines (e.g., IFN‐γ and TNF‐α) in the early stage of infection, making them a key driver of toxic shock syndrome. Excessive activation rapidly induces the functional exhaustion of MAIT cells, characterized by sustained upregulation of inhibitory receptors such as LAG‐3 and TIM‐3, and leads to the loss of MR1‐dependent responsiveness to bacterial intrinsic antigens [109, 144]. This SAg‐mediated biphasic response (“excessive activation–functional exhaustion”) exacerbates the inflammatory response in the early stage of infection and suppresses antibacterial immunity in the later stages, thereby facilitating the immune evasion by gram‐positive bacteria.

### 3.2 Virus

#### 3.2.1 Human Immunodeficiency Virus

The role of MAIT cells in viral diseases was first observed in chronic human immunodeficiency virus (HIV)‐1 infection wherein patients exhibit a dual defect in peripheral blood MAIT cells, characterized by reduced numbers and impaired function [32, 145]. Although antiretroviral therapy (ART) can partially restore the responsiveness of residual MAIT cells to bacterial antigens, the reduction in their numbers is typically irreversible [85, 146]. This phenomenon was initially puzzling, as MAIT cells are predominantly CD8⁺, express CCR5 at high levels, but rarely express CD4, which are properties that should render them less susceptible to HIV infection [147]. Subsequent studies revealed that a small subset of CD4⁺ MAIT cells, owing to their high CCR5 expression levels and specific epigenetic features, can serve as a latent HIV reservoir even after long‐term ART [148]. Importantly, MAIT cell exhaustion exhibits tissue heterogeneity. Their numbers are drastically reduced in the lungs [149] and lymph nodes [40] but relatively preserved in the female reproductive tract and intestinal mucosa suggesting that cell redistribution may be a contributing factor [150]. Notably, MAIT cell exhaustion is less severe in the intestinal mucosa, indicating that the reduction of peripheral blood MAIT cells may partially result from their migration to tissues to counteract microbial translocation [151, 152]. This is consistent with the increased intestinal barrier disruption and microbial translocation observed in MR1‐deficient mice [153]. The degree of MAIT cell exhaustion is closely associated with the level of systemic immune activation. Studies have shown that activation‐induced pyroptosis is a key mechanism driving this phenomenon [154]. Although ART can partially restore MAIT cell function, their numerical and functional deficits persist. This persistence may be related to the inhibitory effect mediated by IL‐10 secreted by monocytes under chronic IFN‐α stimulation [155].

In contrast to what is observed during chronic infection, MAIT cells undergo expansion during acute HIV‐1 infection. Their numbers increase significantly and exhibit high levels of activation in the blood and intestinal mucosa in the early stage of infection [156]. A rhesus macaque model of HIV infection further confirmed this phenomenon by demonstrating upregulated α4β7 integrin expression [157]. This activated state is closely correlated with markers of microbial translocation [158]. Emerging evidence indicates that MAIT cells can be activated by HIV‐1 via cytokine‐dependent (e.g., IL‐12/IL‐18) or non‐TCRdependent mechanisms. Once activated, they secrete chemokines such as CCL3, CCL4, and CCL5, which directly inhibit viral replication by competitively blocking the CCR5 receptor [159]. This effect is virus tropism specific. It is ineffective against CXCR4‐tropic strains (e.g., HIV‐LAI) and does not depend on cell contact or IFN‐γ. A similar phenomenon has been observed in HTLV‐1 infection. The frequency of MAIT cells in the peripheral blood of patients decreases, and residual cells exhibit significant functional impairment despite displaying a highly activated state [160]. Collectively, MAIT cells display a biphasic response during HIV infection. They exert antiviral effects via chemokines in the acute phase but undergo irreversible exhaustion owing to sustained activation in the chronic phase. This mechanism provides a theoretical basis for MAIT cell‐targeted immunotherapeutic strategies.

#### 3.2.2 Respiratory Virus

MAIT cells play a critical and functionally diverse role in the immunity against respiratory viral infections, leveraging their properties of tissue residency and rapid responsiveness [161]. MAIT cells in human lung tissue show high expression levels of cytotoxic effector molecules, such as granzyme and perforin, as well as cytokines with antibacterial activity (e.g., IL‐26). They can also sense inflammatory signals via IL‐12R/IFN‐γR to initiate functional responses. The protective effect of MAIT cells is particularly significant in influenza virus infection. Here, the MAIT cells are activated through an IL‐18‐dependent pathway and secrete IFN‐γ and GZMB to restrict viral spread [97]. Clinical data show that the accumulation of MAIT cells in lung tissue during the early stage of infection is positively correlated with patient survival rate. Animal experiments further confirm that adoptive transfer of MAIT cells significantly reduces the mortality of mice infected with H1N1 influenza virus [73].

In COVID‐19, MAIT cells exhibit a dual role. On one hand, they migrate from peripheral blood to lung tissue and may be activated by infected macrophages in an MR1‐dependent manner, thereby exerting antiviral effects [162, 163]; on the other hand, their excessive activation is associated with disease severity. In patients who are critically ill, MAIT cells show skewed IL‐17A expression, which is closely linked to cytokine storm and coagulation dysfunction. This abnormally activated state is even an independent predictor of patient death [164]. The core of this functional contradiction lies in the timing of activation timing and microenvironmental regulation. During the early stage of infection, the expression of CD69 (an early activation marker) on MAIT cells is correlated with better oxygenation index in patients. However, sustained activation is accompanied by lung tissue damage, suggesting that the functional orientation of MAIT cells is jointly determined by the timing of activation and local microenvironment [165].

Measles virus has evolved a unique MAIT cell evasion strategy. The virus directly induces MAIT cell infection and initiates apoptotic programs by binding with high affinity to the CD150 receptor on the surface of MAIT cells with high affinity. This active clearance mechanism leads to MAIT cell exhaustion, which in turn explains the characteristic immunosuppressive state and increased susceptibility to secondary infections following measles infection [166].

#### 3.2.3 Viral Hepatitis

In patients with chronic hepatitis C virus (HCV) infection, the frequency of MAIT cells is significantly reduced in both the peripheral blood and liver tissue. Eberhard et al. showed that the proportion of peripheral blood MAIT cells in HCV‐infected individuals was only 58% of that in healthy controls (1.35 vs. 2.33%), and this proportion was further decreased in patients with HIV/HCV coinfection [167]. Liver tissue biopsy results also indicated that the proportion of intrahepatic MAIT cells in the HCV‐infected group was significantly lower than that in the noninfected group, and the residual cells showed high expression levels of activation markers such as CD38 and HLA‐DR. A longitudinal follow‐up of seven patients revealed that even after successful antiviral treatment, the number of MAIT cells was not restored to normal levels. This phenomenon of intrahepatic MAIT cell exhaustion contradicts the hypothesis that “cells migrate to sites of inflammation” [79, 167]. These residual MAIT cells continuously exhibit high expression levels of activation molecules (e.g., CD38 and HLA‐DR) and exhaustion‐related molecules (e.g., PD‐1), accompanied by downregulation of the IL‐7 receptor and impaired expression of transcription factors Helios and PLZF. After HCV infection, the MR1‐dependent antigen responsiveness of MAIT cells is severely impaired, whereas their responsiveness to nonspecific cytokine stimulation (e.g., IL‐12/IL‐18) is retained [81]. This functional impairment remains irreversible even after viral clearance. Regardless of whether IFN‐based or direct‐acting antiviral treatment regimens are used, MAIT cells cannot be effectively restored in terms of quantity or function. Unlike other immune cells, the recovery of MAIT cells is difficult to achieve even after successful HCV clearance with IFN‐free treatment regimens. Furthermore, IFN‐α treatment regimens can further inhibit the cytokine responsiveness of MAIT cells [168]. Notably, in vitro experiments have shown that MAIT cells are highly sensitive to type I IFNs. APCs infected with HCV can activate MAIT cells; however, sustained activation may eventually induce cell exhaustion [28].

By contrast, MAIT cells in patients with chronic hepatitis B virus infection exhibit a distinctly different phenotype. Their frequency in peripheral blood and liver is comparable to that in healthy individuals, and they remain highly enriched in liver tissue. The expression of the cell activation marker CD38 is significantly increased, whereas that of exhaustion markers such as PD‐1 and TIM‐3 is not upregulated. Functionally, MAIT cells can still produce IFN‐γ in response to stimulation, and their cytotoxic potential is enhanced. This activated state is associated with viral replication levels. After antiviral treatment with entecavir, the expression of CD38 expression can return to normal levels, whereas the quantity and function of MAIT cells are not significantly affected [78].

### 3.3 Fungus

In recent years, the crucial role of MAIT cells in antifungal immunity has become increasingly clear. Similar to their response to bacterial infections, MAIT cells rely on the MR1‐dependent pathway to initiate responses in antifungal immunity as well. During *Aspergillus spp*. infection, DCs can activate MAIT cells after presenting antigens via MR1. This activation leads MAIT cells to rapidly upregulate the expression of CD69, TNF, and IFN‐γ expression within 4 h, and significantly increase perforin and GZMB levels after 24 h. Ultimately, MAIT cells mediate fungal clearance through direct cytotoxicity, demonstrating functional characteristics of rapid response and efficient antifungal activity [46].

However, fungal pathogens have evolved various strategies to regulate or evade the immune surveillance of MAIT cells. For instance, the secreted effector protein Cmi1 of Candida albicans can target the TBK1 protein in host cells, inhibiting the activation of the type I IFN signaling pathway. This interference impairs the functional activation of MAIT cells, enabling the fungus to achieve immune evasion. Mechanistic validation experiments showed that after infection with *Cmi1‐knockout C. albicans* strains, the IFN‐I response of MAIT cells was significantly enhanced, and the survival rate of infected animal models also increased accordingly. This directly confirms the inhibitory effect of Cmi1 on the antifungal function of MAIT cells [169]. In addition, *C. parapsilosis* can form heterogeneous drug‐resistant subpopulations to evade the early recognition by MAIT cells, leading to the failure of clinical preventive antifungal therapy and subsequent breakthrough bloodstream infections. This has become another important mechanism through which fungi escape MAIT cell surveillance [170].

Notably, the function of MAIT cells in fungal infections is not limited to a single “protective role.” Their functional orientation is jointly regulated by the type of infection (acute/chronic) and microenvironmental signals. In chronic fungal infections, stimulation with IL‐18 leads to the functional deviation of MAIT cells. On the one hand, they inhibit the secretion of the anti‐inflammatory factor IL‐10, and on the other hand, they produce large amounts of proinflammatory factors such as IL‐17A and GM‐CSF. Excessive proinflammatory responses exacerbate tissue damage at the infection site. Further studies revealed that the transcription factor c‐MAF plays a key regulatory role in the anti‐inflammatory function of MAIT cells. The absence of c‐MAF significantly impairs the anti‐inflammatory regulatory ability of MAIT cells, leading to further aggravation of tissue damage [171]. In the context of coinfections, the function of MAIT cells exhibits greater complexity. For instance, during a C. albicans and S. aureus coinfection, the fungus can physically encapsulate the bacteria, helping them evade the killing effect of neutrophil extracellular traps. MAIT cells reverse this evasion phenomenon by secreting GM‐CSF [172].

Beyond the strategies of the pathogens themselves, the regulatory role of environmental factors in the antifungal activity of MAIT cells cannot be ignored. Studies have confirmed that benzaldehyde derivatives present in cigarette smoke can specifically block the antigen‐presenting function of MR1 molecules, thereby inhibiting the activation of MAIT cells. This leads to a weakened antifungal immune response in the body, thereby increasing the risk of developing fungal pneumonia [173]. This finding suggests that environmental factors play a non‐negligible role in the immune regulation of MAIT cells.

### 3.4 Parasites and Rickettsia

Compared with the research on the role of MAIT cells during bacterial, viral, and fungal infections, the study on their role in the immune response against parasitic and rickettsial infections is still in the exploratory stage. However, existing evidence has initially revealed potential roles in acting against these infections [2].

In malaria, MAIT cells may be involved in the host's protective immunity; however, the specific mechanism remains to be elucidated. Studies have shown that the number of MAIT cells decreases significantly in the early stage (11–18 h) of* Plasmodium falciparum* infection, and that a significant expansion occurs in the subsequent months [174]. MAIT cells may directly kill parasite‐infected cells or inhibit pathogen proliferation by releasing cytotoxic mediators and cytokines.

In patients with *Rickettsia tsutsugamushi* infection (scrub typhus), the number of MAIT cells in the peripheral blood decreases while being activated, accompanied by a reduction in TNF‐α secretion and impaired function. The number of these cells is associated with disease severity. This phenomenon is closely related to systemic immune dysregulation, among which the high expression of CD69 may play a key role [175, 176]. Mechanistically, MAIT cells may recognize APCs infected by rickettsiae, initiate activation programs, and participate in the immune response. They regulate the intensity and direction of the immune response by secreting cytokines to assist the body in clearing rickettsial infections. However, the specific molecular pathways of this process need to be thoroughly investigated. The immune mechanisms of MAIT cells during parasitic and rickettsial infections urgently need to be further explored. Their interactions with other immune cells and dynamic changes at different stages of infection are all important directions for future research.

In conclusion, through their unique MR1‐restricted recognition and cytokine response mechanisms, MAIT cells play a central protective role in protecting against bacterial and fungal infections and acute viral invasion. However, they exhibit dual function: excessive activation can lead to immunopathological damage, whereas chronic infections may lead to irreversible functional exhaustion. Furthermore, their powerful effector functions can be exploited by pathogens such as *S. aureus*, serving as a breakthrough for immune evasion. These crucial and complex functions in the immune response to a range of infections make MAIT cells a highly promising target for immunotherapy.

## 4 Regulatory Role of MAIT Cells in Autoimmune Diseases

In addition to playing a crucial role in host defense, a growing body of evidence indicates that MAIT cells are involved in the pathogenesis of various autoimmune diseases. They exhibit significant functional plasticity—they can not only exert protective functions through the rapid production of cytokines and execution of cytotoxicity but also participate in pathogenic processes. This dual function is precisely regulated by the local microenvironment and microbial signals. In this section, following discussion focuses on the abnormal response mechanisms of MAIT cells under autoimmune conditions and evaluates their translational potential as disease biomarkers and therapeutic targets.

### 4.1 Multiple Sclerosis

MAIT cells exhibit a dual role with both protective and proinflammatory effects in the pathological process of multiple sclerosis (MS). Within the central nervous system microenvironment, MAIT cells express antioxidant molecules, such as Selenop and Fth1, at high levels. They maintain the integrity of the meningeal barrier by scavenging reactive oxygen species (ROS) in the meningeal region, thereby inhibiting neuroinflammation and alleviating cognitive impairment. Studies have shown that Mr1^−^^/^^−^ mice lacking MAIT cells exhibit meningeal leakage, abnormal activation of microglia, and defects in learning and memory functions. However, these pathological changes can be reversed by adoptive transfer of MAIT cells or administration of antioxidant therapy [177].

In experimental autoimmune encephalomyelitis (EAE)—a classic animal model of MS—the functional duality of MAIT cells is further highlighted. The MAIT cells infiltrating the brain of EAE models are mainly of the MAIT17 and MAIT1/17 subtypes. Single‐cell transcriptome analysis revealed that these cells simultaneously enrich inflammation‐related pathways and tissue repair‐related pathways, indicating the plasticity of their functions. Further studies have confirmed that TCR‐dependent activation can induce the secretion of the amphiregulatory factor amphiregulin by MAIT cells. This factor can effectively inhibit excessive activation of astrocytes and ultimately reduce the disease severity in EAE models, clarifying the protective role of TCR‐mediated MAIT cell activation in the control of neuroinflammation [178].

With respect to clinical disease characteristics, the phenotypic and functional changes of MAIT cells are specific to MS subtypes. Among patients with clinically progressive MS, those with primary progressive MS (PP‐MS) exhibit unique MAIT cell exhaustion characteristics. Here, the number of CD8⁺MAIT cells in peripheral blood is specifically reduced, and tissue homing‐related subsets (central and effector memory MAIT cells) show significant exhaustion. This phenomenon is not observed in patients with relapsing‐remitting MS or amyotrophic lateral sclerosis, suggesting that MAIT cell exhaustion may be a specific immune biomarker for PP‐MS, thereby presenting a potential immunological indicator for the clinical differential diagnosis of PP‐MS [179].

In addition, in patients with MS, the proinflammatory functional potential of MAIT cells is significantly enhanced mainly manifested by a marked increase in IL‐17 secretion. This phenomenon is positively correlated with the upregulated expression of IL‐7 receptor α chain (IL‐7Rα, CD127) on the surface of MAIT cells [180]. In vitro functional experiments further confirmed that IL‐7 stimulation can significantly amplify the IL‐17 secretion level of MAIT cells in patients with MS and promote the expression of RORγt (a core transcription factor of Th17 cells) and CCR6 (a characteristic chemokine receptor of Th17 cells). These findings suggest that the IL‐7 signaling pathway may be a key regulatory factor driving the differentiation of MAIT cells in patients with MS toward a Th17‐like proinflammatory phenotype, thereby participating in the neuroinflammatory pathological process of MS [180].

### 4.2 Rheumatoid Arthritis

The frequency of MAIT cells in the peripheral blood of patients with early untreated rheumatoid arthritis (RA) is not significantly different from that in healthy individuals or patients with spondyloarthritis. However, the distribution of their subsets shows obvious changes. The proportion of CD4⁺ MAIT cells is abnormally increased, whereas that of CD8⁺ MAIT cells is significantly decreased, accompanied by profound inhibition of CD161 expression. This suggests that the chronic inflammatory microenvironment induces phenotypic remodeling of MAIT cells. These MAIT cells exhibit reduced responsiveness to bacterial stimulation (e.g., immobilized *E. coli*), with a significantly decreased upregulation of activation markers CD25 and CD69, indicating impairment of their immune surveillance function, which may be associated with intestinal flora dysbiosis [181].

This functional impairment is not limited to the initial stage of RA. Recent studies based on high‐dimensional single‐cell technology have further revealed that MAIT cells, together with innate lymphoid cells, such as γδ T cells and group 3 innate lymphoid cells, constitute the peripheral immune signature of patients with RA. Changes in their specific subsets have potential associations with disease activity and treatment response [182]. A longitudinal study conducted by Lien et al. [183] revealed that the expression of MAIT cell signature genes (including *GZMK*, *NCR3*, and *SLC4A10*) in the peripheral blood of patients with RA during pregnancy was continuously downregulated. This reduction remained statistically significant even after excluding the interference of changes in cell proportion, confirming that their functional dysregulation is independent of alterations in the overall immune cell composition [183].

Regarding the core mechanism of immune dysregulation in RA, the pathogenesis of the disease is essentially a synergistic imbalance between innate immunity and adaptive immunity. The abnormal activation of the IL‐23/IL‐17 inflammatory axis is a key pathway driving neutrophil‐mediated joint inflammation [184]. As an important “mediator” between intestinal microbial antigens and local joint inflammation, MAIT cells can not only initiate immune responses by recognizing intestinal microbial metabolites but also participate in regulating the secretion of proinflammatory factors such as IL‐17. Functional defects in MAIT cells may disrupt the balance of “microbial antigen–immune regulation–joint homeostasis,” thereby exacerbating the collapse of local immune homeostasis in joints and ultimately promoting the pathological progression of RA.

### 4.3 Inflammatory Bowel Disease

MAIT cells actively participate in the occurrence and development of inflammatory bowel disease (IBD) by virtue of their MR1‐restricted antigen‐recognition ability and tissue‐homing properties. With respect to disease subtype‐specific characteristics, significant differences exist in the regulatory patterns of MAIT cells between patients with ulcerative colitis (UC) and those with Crohn's disease (CD).

Single‐cell analysis shows that IL‐17A⁺CD161⁺ effector memory T cells are significantly expanded in the colonic mucosa of patients with UC, accompanied by an increased proportion of IL‐17A⁺ regulatory T‐cell subsets. This phenotypic feature is closely associated with the activation of local intestinal Th17‐type inflammatory responses, suggesting that MAIT cells may be involved in the mucosal inflammatory process of UC through IL‐17A‐mediated proinflammatory effects [185]. By contrast, CD is characterized by the distinctive accumulation of IL‐1β⁺ DCs and monocytes in the intestinal tract, and MAIT cells exhibit a distribution pattern of “peripheral reduction–local accumulation,” wherein the frequency of MAIT cells decreases in peripheral blood along with significant accumulation in inflamed intestinal tissues. This phenomenon clearly indicates the specific migration of MAIT cells to intestinal lesion sites [186].

Altered activation status of MAIT cells is a common feature in IBD; however, it also exhibits subtype differences. In patients with IBD, the expression level of the activation marker HLA‐DR⁺CD38⁺ on the surface of MAIT cells is significantly increased, and the costimulatory molecule NKG2D and inhibitory receptor BTLA are upregulated simultaneously, reflecting the synergistic imbalance between activation and regulatory signals. Functionally, the ability of MAIT cells to secrete IL‐17 is significantly enhanced (this feature is more prominent in patients with UC), whereas MAIT cells in the peripheral blood of patients with CD show a characteristic IL‐1β⁺ phenotype. These findings further confirm the differences in functional polarization of MAIT cells in different subtypes of IBD [185]. This functional polarization is closely associated with intestinal flora dysbiosis—intestinal flora involved in riboflavin metabolism can activate MAIT cells through MR1‐dependent antigen presentation and drive their directional homing to the intestinal mucosa via chemokine axes such as CCR6–CCL20 and CCR9–CCL25. Tissue distribution studies show that under physiological conditions, MAIT cells are mainly localized in the intestinal lamina propria, with a small number present in the epithelium, and their abundance in the cecal epithelium is higher than that in the colon. In the pathological state of IBD, MAIT cells exhibit a significantly enhanced intestinal infiltration ability owing to the interaction between α4β7 integrin and mucosal addressin cell adhesion molecule‐1. Further verification using a random forest model reveals that the tissue‐specific expansion of MAIT cells can serve as one of the potential immune biomarkers for distinguishing UC from CD, providing a new immunological perspective for the subtype differentiation of IBD subtypes [29]. Notably, MAIT cells may exert a dual role of “proinflammation and tissue repair” in IBD. On the one hand, they can exacerbate intestinal mucosal inflammation by secreting proinflammatory factors such as IL‐17 and IL‐22; on the other hand, studies involving animal models of colitis have shown that they possess tissue repair potential [186]. This functional plasticity does not occur randomly but is finely regulated by microbial metabolites and cytokines in the local intestinal microenvironment, which ultimately determines the functional orientation of MAIT cells in the pathological process of IBD.

### 4.4 Autoimmune Hepatitis

In patients with autoimmune hepatitis (AIH), MAIT cells exhibit the characteristics of concurrent numerical reduction and functional impairment, and such abnormalities are resistant to standard immunosuppressive therapy. Yuksel et al. [187] showed that the frequency of MAIT cells in the peripheral blood of untreated pediatric patients with AIH is significantly decreased, and that the number of MAIT cells in the liver is also notably lower than that in healthy individuals. Even after immunosuppressive therapy, the number of MAIT cells does not show significant recovery, suggesting that the loss of MAIT cells may impair liver immune surveillance function and contribute to the chronicity of the disease [187]. A study by Renand et al. [188] on adult patients with AIH further revealed the functional abnormalities and pathological mechanisms of MAIT cells. Although MAIT cells in adult patients with AIH display an activated phenotype with high GZMB expression, their core effector functions (such as IFN‐γ secretion) are significantly impaired, presenting a contradictory state of “activation–exhaustion.” Moreover, the number of GZMB⁺ MAIT cells infiltrating the liver tissue is positively correlated with the severity of liver fibrosis [188]. This result indicates that activated MAIT cells may directly damage hepatocytes or activate hepatic stellate cells through GZMB‐mediated cytotoxicity, thereby driving the process of liver fibrosis. This provides a new cellular‐level explanation for the mechanism of liver injury in AIH.

### 4.5 Systemic Lupus Erythematosus

In patients with systemic lupus erythematosus (SLE), the frequency of peripheral MAIT cells is significantly reduced, and the degree of reduction is negatively correlated with disease activity (e.g., SLEDAI score), which is particularly prominent in patients with active lupus nephritis (LN) [100]. This phenomenon arises from a dual mechanism. On the one hand, MAIT cells in patients with SLE undergo abnormal activation, characterized by increased expression of surface activation markers CD69 and CD25. Excessive activation further induces an increase in “activation‐induced cell death,” directly leading to the loss of cell numbers [100]. On the other hand, MAIT cells have an inherent defect in the calcium/calcineurin/NFAT1 signaling pathway. This defect impairs the nuclear translocation of the NFAT1 transcription factor, thereby directly inhibiting the production of IFN‐γ and laying the foundation for the functional exhaustion of MAIT cells [189]. In addition, MAIT cells may participate in local pathological damage by migrating directionally to inflamed sites, such as the kidneys, and this process also exacerbates the reduction in the number of MAIT cells in peripheral blood. Notably, the defects of MAIT cells in patients with SLE are significantly associated with the numerical reduction and functional abnormalities of NKT cells. Both cell types coexpress the immune checkpoint molecule PD‐1 at high levels, suggesting that there is a common immune pathway dysregulation governing MAIT and NKT cells in SLE, and PD‐1‐mediated inhibitory signals may be one of the key nodes driving the functional abnormalities of both these two cell types [189].

At the functional level, MAIT cells in patients with SLE undergo significant functional polarization and remodeling—although IFN‐γ secretion is significantly decreased, proinflammatory and cytotoxic characteristics are remarkably enhanced, and this remodeling is more prominent in patients with LN [189]. Specifically, after stimulation with PMA/ionomycin, IL‐17 and GZMB expression levels in MAIT cells of patients with LN are significantly increased, and this increasing trend is more obvious, particularly in patients with proliferative glomerulonephritis (class III/IV) as the pathological type. Further analysis shows that the “high GZMB, low CD56” cytotoxic phenotype of MAIT cells is directly correlated with disease severity and can serve as an important functional indicator reflecting the pathological progression of SLE (particularly LN) [190].

Clinical prognostic studies have further confirmed the clinical value of MAIT cells—patients with LN with a higher frequency of peripheral MAIT cells, lower expression of the proliferation marker Ki‐67, and lower GZMB expression at baseline are more likely to achieve complete renal remission after receiving immunosuppressive induction therapy [190]. This result indicates that the cytotoxic activity of MAIT cells can be used as a potential biomarker for predicting the response to immunosuppressive therapy in patients with LN. This provides a new indicator for the precise clinical evaluation and optimization of treatment regimens for SLE.

### 4.6 Type 1 Diabetes Mellitus

Abnormalities in the number and function of MAIT cells in patients with type 1 diabetes mellitus (T1D) have become a central focus for research. Clinical studies have shown that the frequency of MAIT cells in the peripheral blood of children with newly diagnosed T1D is significantly reduced, accompanied by increased expression of activation/exhaustion markers (CD25, PD‐1). In terms of functional abnormalities, these MAIT cells exhibit increased secretion of GZMB and TNF‐α, along with decreased secretion of IL‐17A/IL‐22. This suggests that MAIT cells migrate to inflamed tissues and participate in pathological damage [191]. Further analysis revealed that the cytotoxic phenotype of MAIT cells with high GZMB expression is negatively correlated with the early onset age and disease severity in children with T1D, providing a potential indicator for evaluating disease progression [191].

Studies using animal models have further revealed the tissue‐specific functional differences of MAIT cells in T1D. In nonobese diabetic (NOD) mice, as the disease progresses, MAIT cells gradually accumulate in pancreatic islet tissue. These cells can secrete GZMB and IFN‐γ and directly promote the destruction of pancreatic β‐cells. By contrast, MAIT cells in intestinal tissue maintain the integrity of the intestinal mucosal barrier by secreting IL‐17A and IL‐22, demonstrating a dual functional characteristic of “pathogenesis–protection” [192]. This tissue‐specific functional imbalance is further confirmed in MR1‐deficient NOD mice. These mice show a significantly increased incidence of diabetes, accompanied by elevated intestinal permeability, decreased expression of intestinal tight junction proteins, and abnormal expansion of islet‐reactive T cells. This suggests that MR1‐mediated regulation of MAIT cells is crucial for maintaining intestinal homeostasis and islet immune balance. Mechanistically, DCs present bacterial metabolites via MR1 molecules to regulate MAIT cell differentiation. Under homeostatic conditions, MAIT cells tend to secrete protective cytokines; however, under conditions of intestinal flora dysbiosis or increased barrier permeability, inflammatory signals drive MAIT cells to differentiate into a pathogenic phenotype [193].

In the context of clinical translation, studies have proposed the use of the phenotypic characteristics of MAIT cells, such as frequency, expression of activation/exhaustion markers, and cytokine secretion profile, as potential biomarkers for assessing T1D risk [191]. Future intervention strategies targeting MAIT cells need to precisely balance their protective and pathogenic roles. Through targeted regulation, the synergistic effects of “enhancing intestinal barrier function” and “inhibiting islet tissue infiltration and damage” can be achieved, providing new perspectives for the precise treatment of T1D.

In brief, MAIT cells play a dual “protective–destructive” role in autoimmune diseases—they can maintain barriers and repair tissues but also exhibit abnormal activation to exacerbate inflammatory damage. This functional imbalance is precisely regulated by the local microenvironment and microbiota–immune interactions. MAIT cells have emerged as highly promising disease biomarkers and therapeutic targets. Future research should focus on their tissue‐specific regulatory mechanisms to develop precise immune intervention strategies.

## 5 The Contradictory Role of MAIT Cells in the TME

The TME is a dynamic niche composed of tumor cells, immune cells, stromal cells, extracellular matrix, and hypoxic/acidic signals. Its immunosuppressive properties drive immune evasion through inhibitory factors (TGF‐β, IL‐10), PD‐L1 expression, and the expansion of Tregs/myeloid‐derived suppressor cells (MDSCs) [194]. MAIT cells exhibit significant functional plasticity in the TME. In terms of antitumor effects, once activated, they secrete IFN‐γ/TNF‐α and cytotoxic molecules (GZMB, perforin), directly killing specific tumor cells and enhancing immune surveillance mediated by DCs/NK cells; in terms of protumor effects, under the influence of chronic stimulation, hypoxia, acidic pH, and inhibitory factors, MAIT cells undergo exhaustion and secrete IL‐17/IL‐8/MMPs, which promote tumor proliferation, invasion, and metastasis. Additionally, they strengthen immunosuppression by inhibiting the functions of effector T/NK cells and expanding Tregs/MDSCs/tumor‐associated macrophages (TAMs) [98, 195, 196, 197, 198, 199, 200, 201, 202]. The functional polarization of MAIT cells is dynamically regulated by tumor types and local microenvironmental signals. Targeted strategies (reversing exhaustion, blocking pathogenic subsets, adoptive therapy) provide new approaches for reshaping antitumor immunity. Here, we will introduce the frequency, functions, and other aspects of MAIT cells in tumors. This knowledge will pave the way for harnessing MAIT cells to enhance tumor immunity (Figure 4 and Table 2).

**TABLE 2:** Characteristics and clinical significance of MAIT cells in tumors.

Disease type	Frequency (tissue)	Frequency (blood)	Phenotype (tissue)	Phenotype (blood)	Function (tissue)	Function (blood)	Specific function and clinical significance	References
HCC	↓	↓	CD4−MAITs ↓ CD28/CD127/CCR6/CXCR6/CCR9↓ CD38/HLA‐DR/CXCR3/CCR2/CCR5^high^‐CTLA4/TIM‐3/PD‐1↑ BAX/BID/Bcl‐2‐	CD4^−^MAITs↓ CCR7‐CD45‐RA‐CD45RO^+^CD95^+^CD38/HLA‐DR‐ CD160↓ CTLA4/TIM‐3/PD‐1↑	IL‐8↑ IFN‐γ/IL‐17/granzyme B/perforin↓ IL‐4/IL‐10/IL‐22‐	IFN‐γ/IL‐17↓	MAIT cells in infiltrating liver cancer tend to favor tumor promotion, which is unfavorable for patient prognosis. TCGA liver cancer patients with low expression of the MAIT marker gene SLC4A10 have a poorer prognosis.	[200, 203]
CRLM	↓	↓	GZMB^+^ MAIT cells↓ IL‐12R/IL‐18R‐	—	IFN‐γ/granzyme B↓	—	There is extensive dysfunction of MAIT cells in CRLM that is determined by their physical location rather than by preoperative chemotherapy.	[204]
CCA	↓	—	CD69/CD103/Ki‐67↑ CD56/CXCR6/CCR6/PD‐1/HLA‐DR↓	—	Perforin/granzyme B↓	—	Tumor infiltration by MAIT cells is associated with good immune adaptability and predicts the survival rate of cholangiocarcinoma.	[205]
CRC	↑	↓/‐	CD45RO/CD69↓	CD45RO^+^IL‐18Rα^+^CD8^+^↓	IFN‐γ↑IL‐17↑	TNF‐α/IFN‐γ↓IL‐17A↑	The serum CEA levels of CRC patients are positively correlated with the percentage of tumor‐infiltrating MAIT cells, but negatively correlated with the percentage of circulating MAIT cells in late‐stage CRC patients. *Tumor‐infiltrating MAIT cells exhibit significantly reduced ability to produce IFN‐γ. *Patients with high infiltration of MAIT cells in the tumor have a worse prognosis.	[206, 207, 208]
EC	↑	↓	NKG2D↓	—	IFN‐γ/TNF‐α↓	—	MAIT cell levels are not affected by radiotherapy and chemotherapy treatment, unlike other T cell types.	[209]
GC	↑	↓	—	CCR6/CXCR6↑	IFN‐γ↓	—	The reduced circulation of MAIT cells in MAC patients is due to their migration to mucosal cancer tissues.	[98]
NSCLC	↑	↑	CXCR6⁺CD8⁺↑	CXCR6⁺CD8⁺↑	GNLY/PRF1/NKG7↑	—	MAIT cells primarily migrate from peripheral blood mononuclear cells (PBMCs) to tumor tissues mainly through the CCR6–CCL20 axis.	[210]
AML	↑	↓	—	—		—	—	[211, 212]
MM	↓	↓	—	—	CD27/IFN‐γ↓	—	The significant decrease in MAIT cell frequency may be associated with increased apoptosis or impaired survival ability.	[7, 213]

### 5.1 Lung Cancer

Lung cancer is a highly malignant primary tumor originating from bronchial mucosal epithelium or alveolar epithelium. As the leading cause of cancer‐related deaths worldwide, its pathogenesis is closely related to tobacco exposure, environmental carcinogens, and genetic susceptibility, causing more than 2 million deaths globally each year [214]. MAIT cells exhibit unique expression patterns, functional complexity, and prognostic value in the lung cancer microenvironment, and their biological behaviors are finely regulated by tissue localization, immunometabolism, and the local microenvironment. In the peripheral blood and tumor tissues of patients with non‐small cell lung cancer (NSCLC), MAIT cells, especially the CD8⁺ subset, show significant enrichment and high expression of the chemokine receptor CXCR6; this feature is closely related to the responsiveness to anti‐PD‐1/PD‐L1 immunotherapy [202, 215, 216]. Single‐cell transcriptome analysis reveals that CXCR6⁺ CD8⁺ MAIT cells enhance interactions with the TME through the CXCL16–CXCR6 axis, and their cytotoxicity‐related genes (such as *GNLY*, *PRF1*, *NKG7*) are significantly upregulated, promoting effector T cell infiltration and directly killing tumor cells [210]. In addition, similar studies, based on secondary mining of published data, also found that the abundance of MAIT cells in lung cancer tumor tissues is higher than that in adjacent noncancerous tissues, and MAIT cells are mainly driven to migrate from peripheral blood mononuclear cell (PBMC) to tumor tissues through the CCR6–CCL20 axis [217]. However, the function of MAIT cells is dual. In NSCLC patients with chronic obstructive pulmonary disease (COPD), although the number of tumor‐infiltrating CD8⁺ MAIT cells increases, they show a functionally exhausted phenotype, characterized by reduced secretion of GZMB and IFN‐γ, and increased expression of the immune checkpoint PD‐1. This exhausted state impairs their antitumor activity but is positively correlated with the responsiveness to neoadjuvant immunotherapy [173, 216]. This paradoxical phenomenon may be related to the metabolic reprogramming of MAIT cells. Under the influence of carcinogens such as tobacco smoke, the metabolic pathways of MAIT cells are dysregulated, leading to impairment of their immune function. Tobacco components (such as benzaldehyde derivatives) bind to MR1 protein and block the activation of MAIT cells by bacterial metabolites, further exacerbating the susceptibility to pulmonary infections and tumor progression [171, 218].

The value of MAIT cells as prognostic biomarkers is also controversial. A prospective study reported that an increased frequency of activated CD8^⁺^CD38^⁺^ MAIT cells in peripheral blood is significantly associated with shorter progression‐free survival (PFS) in lung cancer patients, and this population coincides with elevated serum proinflammatory factor levels, suggesting that it may serve as an early warning indicator for disease progression. However, the Sundstrom team failed to replicate the correlation between MAIT cell characteristics and ICI efficacy in an independent cohort, and there was no significant difference in the MAIT cell lineage between treatment responders and progressive patients. This contradictory result may be due to the heterogeneity of the study population or differences in detection methods [219]. Further subset analysis shows that circulating CXCR6⁺ CD8⁺ MAIT cells can serve as an effective predictive marker for the response to NSCLC immunotherapy. MAIT cells with high CXCR6 expression are enriched in the peripheral blood of immunotherapy responders, and their levels are significantly associated with prolonged PFS [210]. Conversely, the accumulation of intratumoral MAIT cells is positively correlated with the responsiveness to immunotherapy in COPD‐related lung cancer, but high PD‐1 expression indicates functional suppression, requiring combined immune checkpoint blockade (ICB) to restore their activity. In summary, as a bridge connecting innate and adaptive immunity, MAIT cells’ expression profiles, functional plasticity, and metabolic adaptability collectively shape the immune microenvironment of lung cancer. They not only provide new biomarkers for prognosis prediction but also open up new paths for combined immunotherapy. Future studies need to focus on the precise regulation of their subsets to overcome the therapeutic bottlenecks caused by functional heterogeneity.

### 5.2 Blood Cancer

Hematological malignancies are a group of malignant diseases originating from hematopoietic stem cells (HSCs) or progenitor cells, encompassing subtypes such as leukemia, lymphoma, and MM. They are characterized by abnormal cell proliferation, blocked differentiation, and immune dysfunction [220]. At the expression level, the quantity and phenotype of MAIT cells in patients with hematological malignancies show significant heterogeneity. In patients with MM at initial diagnosis, the frequency of MAIT cells in peripheral blood and bone marrow is significantly reduced, with no obvious enrichment in the bone marrow. This suggests that the reduction is not due to migration to the TME but may be related to increased apoptosis or impaired survival. Meanwhile, the expression of PD‐1 on their surface is elevated, and their function can be partially restored after PD‐1 antibody blockade [7, 213]; in patients with AML, the frequency of MAIT cells in the blood decreases, which is closely related to cytogenetic characteristics. Patients with adverse cytogenetic subtypes have a lower frequency of MAIT cells, while those carrying FLT3–ITD or IDH1/2 mutations have an increased frequency of MAIT cells. In addition, clonal infiltration of MAIT cells can be seen in the bone marrow of patients with NK‐type AML (M4/M5), and the infiltration degree is higher in those who do not achieve complete remission [211, 212]. In patients with chronic lymphocytic leukemia (CLL), the CD8⁺CD26^hi^ T cell subset is reduced, and MAIT cells dominate this subset, suggesting that CLL may affect MAIT cell homeostasis [221, 222].

In terms of functional mechanisms, MAIT cells play a dual role in hematological malignancies. On the one hand, activated MAIT cells can directly kill tumor cells by releasing cytotoxic molecules such as perforin and granzyme. For example, MM cell lines can be effectively killed by MAIT cells in the presence of the MR1 ligand 5‐OP‐RU. At the same time, MAIT cells secrete cytokines such as IFN‐γ and TNF‐α to activate DCs, enhance adaptive immune responses, and synergize with NK cells to inhibit tumor progression [222]; on the other hand, immunosuppressive factors in the TME can induce functional exhaustion of MAIT cells. MAIT cells in MM and AML patients highly express inhibitory receptors such as PD‐1 and TIM‐3, and cytokines such as IL‐10 and TGF‐β can weaken their cytokine secretion ability, or even make them switch to a proinflammatory phenotype that secretes IL‐17A, thereby promoting tumor angiogenesis [213, 223]. It is worth noting that the interaction between MAIT cells and other immune cells is complex. For example, in the allo‐hematopoietic stem cell transplantation (HSCT) model, donor MAIT cells can reduce graft‐versus‐host disease (GVHD) by inhibiting CD4+T cell responses, and their protective effect is related to IL‐17 secretion and intestinal microbiota regulation. However, some studies also suggest that MAIT cell infiltration may be associated with poor prognosis in AML patients, and the mechanism may be related to apoptosis caused by excessive activation or a proinflammatory phenotype.

In terms of prognostic evaluation, the quantity and functional status of MAIT cells are closely related to the clinical outcomes of patients with hematological malignancies. In MM and AML patients, those with reduced frequency of peripheral blood MAIT cells at initial diagnosis or high expression of PD‐1 in bone marrow MAIT cells have significantly shortened disease‐free survival and overall survival (OS), as well as a reduced response rate to chemotherapy [213]. In allo‐HSCT, a higher number of donor‐circulating MAIT cells is associated with a reduced posttransplant tumor recurrence rate and a lower risk of GVHD. Moreover, the diversity of the patient's intestinal microbiota is positively correlated with MAIT cell reconstitution, and the microbiota rich in riboflavin‐producing bacteria can promote MAIT cell activation and improve survival prognosis [224, 225, 226]. However, in AML patients, MAIT cell activation is related to adverse cytogenetic characteristics. Although patients carrying FLT3–ITD mutations have an increased frequency of MAIT cells, their prognosis is worse, suggesting that their functional status may be regulated by tumor molecular characteristics [211]. In addition, the impact of treatment methods on MAIT cells also indirectly affects the prognosis. Traditional chemotherapeutic drugs such as cyclophosphamide can significantly deplete MAIT cells, while PD‐1 inhibitors can partially reverse their exhausted state and restore cytotoxic functions. Improvement in MAIT cell function after PD‐1 blockade has been observed in MM patients [227, 228].

### 5.3 Liver Cancer

HCC is the sixth most common cancer and the fourth leading cause of cancer‐related death worldwide [229]. HCC often originates from viral infections or chronic inflammation caused by fatty liver and is complexly regulated by multiple factors, such as genetics, viruses, and the environment. The formation of a strong immune‐tolerant microenvironment in HCC leads to long‐term immune hyporesponsive status in patients, making them more susceptible to developing resistance to tumor immunotherapy [230]. Cytotoxic T lymphocytes (CTLs) are the “main force” involved in killing tumors in liver cancer. High infiltration indicates a good prognosis for patients [231]. As a special type of mucosa‐associated invariant T cell, MAIT cells have unique transcriptomes and phenotypes, which is also the main reason explaining their enrichment in the liver. However, their role in HCC remains unclear [34, 203, 232]. On the one hand, owing to significant differences in the distribution of MAIT cells in the peripheral blood and liver between mice and humans, MAIT cells are generally rarely studied in mice. On the other hand, MAIT cells exhibit significant heterogeneity among patients, and there are currently no systematic studies on this topic [28].

Existing studies suggest that the frequency of MAIT cells in HCC is lower in healthy adjacent liver tissue [200, 203, 215, 232, 233]. Analysis of MAIT cells from HCC patients revealed significant downregulation of trafficking‐related receptor factors such as CCR6, CXCR6, and CCR9, which may be the main factors affecting the transport and settling ability of MAIT cells in HCC progression [200]. Moreover, the number of MAIT cells in the peripheral blood of HCC patients also decreases and is related to lymphocyte counts, tumor stage, carcinoembryonic antigen levels, and tumor diameter; however, no correlation was found with age or T stage [98, 233]. The team led by Zheng and collaborators were the first to depict the immune landscape in the HCC microenvironment at the single‐cell level [203]. Based on the analysis of single‐cell TCR data, researchers have found many tumors tissue‐specific clonal expansion T cells within HCC, but most of these cells are in a state of exhaustion, revealing the reason that tumor cells evade immune surveillance. The present study also demonstrated that, compared with those in adjacent normal liver tissue, the number of MAIT cells in tumors was significantly lower. This finding was further confirmed by The Cancer Genome Atlas (TCGA) dataset, which revealed low expression levels of the MAIT marker gene SLC4A10 in HCC patients [203]. In addition to their decreased frequency, the phenotype, function, and immunoregulatory role of MAIT cells in HCC seem to be affected. Their antitumor function is significantly weakened, and they are reprogrammed in a protumor direction. Duan et al. evaluated MAIT cell distribution, phenotype, and function in the peripheral blood and tissues of HCC patients via flow cytometry and in vitro bioassays and conducted transcriptome analysis [200]. They reported that MAIT cells influenced by tumors significantly upregulate inhibitory molecules such as PD‐1, CTLA‐4, and TIM‐3, whereas CD160, KLRG1, IFN‐γ, and IL‐17 secretion is significantly reduced. Additionally, after coculture of APCs pretreated with *E. coli* with MAIT cells from different sources, MAIT cells from tumor tissues secreted lower levels of perforin and GZMB than did those from adjacent nontumor tissues and normal liver tissues; however, the levels of tumor‐promoting cytokines (such as IL‐18) were significantly increased. These findings suggest that the function of infiltrating MAIT cells in HCC is impaired and may even be reprogrammed to shift from antitumor immunity toward tumor‐promoting effects [200]. Huang et al. reported differences in the activity and function of MAIT cells in the peripheral blood and liver of HCC patients [233]. Specifically, MAIT cells in the livers of HCC patients express high levels of activation markers and exhaustion markers, including HLA‐DR, CD69, and PD‐1, which are not expressed in the peripheral blood [233]. Yao et al. used public single‐cell and bulk transcriptome data to reveal the role and phenotypic characteristics of MAIT cells in human malignancies [232]. They reported that MAIT cells in HCC express higher levels of T‐cell activation markers (CD38, HLA‐DRA) and exhaustion markers (PD‐1, CTLA4, HAVCR2) but lower expression levels of effector function‐related genes such as CD160 and KLRG1, which is consistent with the findings of previous studies [232]. One of the reasons for the low functionality of MAIT cells in tumors may be the downregulation of cytokine receptors such as IL7R, IFNGR1, IL18R1, and IL23R. These receptors mediate the activation of MAIT cells and regulate their activity and immune responses, playing important roles in tumor development and treatment [34, 234]. Furthermore, the study revealed that MAIT cells within HCC tumors expressed relatively high levels of the effector genes GZMB and IFN‐γ, which seems contradictory to previous findings.

In the immunological microenvironment of HCC, the function of MAIT cells is also regulated by other immune cells. Professor Tim F. Greten and his team analyzed MAIT cells in human and murine HCC via CO‐Detection by IndEXing imaging technology, flow cytometry analysis, and single‐cell RNA sequencing (scRNA‐seq) technology. They reported that under the influence of the tumor‐suppressive microenvironment, the tumor infiltration, activation level, and cytotoxicity of MAIT cells were reduced. Researchers subsequently used a weakly supervised convolutional neural network model (S^3^‐CIMA) to analyze the identification and characterization of tissue‐specific cell populations in the cellular microenvironment, thereby analyzing cell‒cell interactions. These results indicate that the loss of MAIT cell function is closely related to PD‐L1^⁺^ TAMs. In a mouse model, PD‐L1 checkpoint blockade inhibited the growth of primary liver cancer tumors. Additionally, liver CD163^⁺^ macrophages suppressed the function of patient‐isolated MAIT cells in vitro, explaining the heterogeneity of MAIT cells in HCC and providing a new perspective for PD‐1/PD‐L1 checkpoint blockade therapy for liver cancer [235]. Existing studies classify MAIT cells into two main subgroups, MAIT‐1 and MAIT‐17; however, the existence of new subgroups of MAIT cells and the dynamic changes and mechanisms of action of MAIT cell subgroups in liver cancer remain unclear. Fu et al. conducted phenotype and functional studies on MAIT cells from liver cancer patients using single‐cell RNA sequencing and flow cytometry, confirming the existence of a group of FOXP3^⁺^CXCR3^⁺^ MAIT cells in liver cancer patients [236]. These cells express high levels of Treg‐related molecules and can effectively suppress T‐cell proliferation. Importantly, MAITregs are present only in the peripheral blood of HCC patients and not in the peripheral blood of healthy individuals. Further research revealed that both FOXP3^−^CXCR3^⁺^ MAIT cells and MAITregs express high levels of β1‐adrenergic receptor (ADRB1) and that ADRB1 signaling promotes the differentiation and function of MAITregs through the cAMP–PKA pathway. Overall, this work identified a new group of immunosuppressive MAITregs that promote the development of liver immune tolerance in liver cancer patients and suggested that neuroimmune crosstalk may be involved in the regulation of liver immunity and the progression of liver cancer [236]. Interestingly, the coadministration of 5‐OP‐RU and the TLR9 agonist CpG strongly induced MAIT cell expansion and invasion, resulting in high expression of CD69, a significant effector memory phenotype, and the upregulation of effector molecules, including IFN‐γ, GZMB, and perforin. MAIT cell induces a strong and broad antitumor immune response in models of liver metastasis, HCC, lung metastasis, and subcutaneous tumors in mice [8].

There are currently conflicting views regarding the relationship between the infiltration level of MAIT cells and the prognosis of patients with HCC. Some studies have reported a significant correlation between the percentage of MAIT cells in HCC patients and the levels of alanine aminotransferase (ALT) and aspartate aminotransferase (AST), which may indicate poor clinical outcomes [233]. Duan et al. [200] evaluated the relationship between the tumor‐infiltrating MAIT cell density and the prognosis of HCC patients using flow cytometry, quantitative reverse transcription polymerase chain reaction, and immunohistochemical staining. These researchers reported that high levels of infiltrating tumor MAIT cells were significantly and independently associated with poor clinical outcomes in four independent cohorts [200]. Zheng et al. [203] identified SLC4A10 as a marker gene for MAIT cells. Using the HCC cohort in TCGA, they reported that lower expression of SLC4A10 was associated with poor prognosis [203]. Yao et al. [232] used scRNA‐seq datasets of HCC, CRC, and NSCLC samples to define a set of MAIT cell marker genes (*SLC4A10*, *KLRB1*, *ME1*, *TMIGD2*, *COLQ*, *RORC*, *ZBTB16*, *TLE1*, *IL23R*, *NCR3*, and *LST1*) that were stably highly expressed in MAIT cells. In the HCC cohort from TCGA, no prognostic associations were found between the MAIT score and OS or PFS [232]. These conflicting results may be due to the following reasons: (1) Different cohorts with different patient characteristics were used; (2) different MAIT cell marker genes were utilized (*TCRvα–Jα33* [200], *SLC4A10* [203], *KLRB1*, *ME1*, *TMIGD2, IL23R*, *NCR3*, *LST1*, *COLQ*, *RORC*, *ZBTB16*, and *TLE1* [232]); (3) varied MAIT cell subgroups and different activation and exhaustion statuses. Given these challenges, Fu et al. [236] conducted a comprehensive analysis of MAIT cell subgroups and reported that the proportion of MAITregs is positively correlated with poor clinical prognosis. Further analysis of the relationships between MAIT cell subgroups and prognosis remains a key focus of future research.

### 5.4 Cholangiocarcinoma

Cholangiocarcinoma (CCA) refers to malignant tumors arising from the epithelium lining the bile ducts. The site of occurrence can be classified into two main categories: intrahepatic cholangiocarcinoma and extrahepatic cholangiocarcinoma. Statistics show that CCA accounts for approximately 15% of all liver and bile duct tumors, making it the second most common malignancy of the liver. Its incidence is increasing annually worldwide [237, 238]. MAIT cells are located in the bile duct and can release cytokines and cytotoxic granules. Zimmer and colleagues comprehensively characterized tumor‐infiltrating MAIT cells in patients with CCA [205]. They reported that, compared with those in surrounding tissues, the presence of MAIT cells in tumors decreased or were lost to varying degrees, and this effect is potentially related to an increase in the bacterial load in the CCA TME. Like chronic inflammation, sustained bacterial stimulation leads to a decrease in the number of MAIT cells. Furthermore, a study of two independent cohorts revealed that patients with high MAIT cell levels have longer median survival, independent of clinical prognostic factors and other immune cells [205]. In the future, increasing our understanding of the composition and function of MAIT cells in the immune microenvironment of CCA will increase the potential for immunotherapy.

### 5.5 Colorectal Cancer

CRC is a malignant tumor originating from the epithelium of the colon and rectum. Internationally, CRC has a prevalence of 10%, placing it third in terms of incidence, and a mortality rate of 9.4%, ranking it second [239]. The survival rate of CRC patients is closely related to the stage at diagnosis, with a 5‐year survival rate of 90% for early‐stage diagnosis. In contrast, this value is only 13% for advanced‐stage disease [240]. The development and progression of CRC involve processes such as local mucosal infiltration, regional spread, and distant metastasis, with the participation of multiple genes and signaling pathways [241]. Multiple studies have confirmed that tumor immune escape is closely related to the occurrence and development of CRC [242, 243]. Owing to the abundant presence of MAIT cells in the colon and their important role in immune regulation and interactions with the microbiota, researchers are strongly interested in their function, making them among the most extensively studied T cells in CRC.

Multiple studies on CRC patients have confirmed that the frequency of MAIT cells in malignant tissue is greater than that in healthy adjacent tissues, especially in the tumor tissue of late‐stage CRC patients [98, 197, 206, 207]. In contrast, the number of MAIT cells in the blood of CRC patients is significantly reduced. Analysis revealed that the number of circulating MAIT cells (which express high levels of CCR6 and CXCR6) decreases due to their migration to mucosal cancerous tissues. This finding suggests MAIT cell infiltration and aggregation at the site of the lesion [98, 206]. Furthermore, a negative correlation exists between the number of circulating MAIT cells and tumor size; therefore, a decrease in the number of circulating MAIT cells is considered an indicator of the extent of cancer progression [98].

Although existing research has confirmed the presence of activated MAIT cells in the microenvironment of CRC, controversy still exists regarding their reactivity and whether they contribute to antitumor immunity or disease progression. To assess the antitumor effects of MAIT cells, Ling et al. cocultured HCT116 (human colon cancer cell line) and K562 (human erythroleukemia cell line) cells with MAIT cells [206]. They reported that activated MAIT cells produced cytokines (TNF‐α, IFN‐γ, and IL‐17) and, during the coculture process, upregulated the expression of cytotoxic markers (perforin and GZMB), increased CD107a expression, and caused cell cycle arrest (G2/M phase) in HCT116 cells in a cell contact‐dependent manner. These effects reduced HCT116 and K562 cell viability. These findings indicate a direct cytotoxic effect of MAIT cells on CRC cells [206]. Sundström et al. analyzed the frequency, phenotype, and function of MAIT cells in CRC and unaffected mucosa via flow cytometry [207]. They reported that regardless of tumor stage or location, significant accumulation of MAIT cells was detected in tumor tissues. Most MAIT cells in unaffected colon tissue produce IFN‐γ, TNF‐α, IL‐2, and GZMB, with only a few producing IL‐17. In tumors, the frequency of MAIT cells producing IFN‐γ was significantly reduced, whereas other cytokine analysis results revealed no difference. In vitro studies demonstrated that factors secreted by tumor tissues reduce the generation of IFN‐γ in MAIT cells. These findings suggest that MAIT cells have the ability to promote local immune responses in tumors, but the TME selectively decreases the Th1‐type response of MAIT cells [207]. However, Ruf et al. [235] obtained different results. These data indicate that infiltrating MAIT cells secrete Th1‐related cytokines and possess the ability to directly kill tumor cells without being affected. In addition to the release of classical Th1 and Th17 cell cytokines after activation, chronic stimulation can also lead to strong IL‐13 expression by MAIT cells. IL‐13 belongs to the IL family of inflammatory regulatory factors and plays multiple roles in tumors, including promoting tumor cell proliferation and invasion, as well as suppressing immune responses [244, 245]. Specifically, Kelly et al. [246] used RNA‐seq and qRT‐PCR to demonstrate high IL‐13 gene expression in chronically stimulated MAIT cells and identified IL‐13 directly via intracellular flow cytometry and multiplex bead analysis of MAIT cell cultures. Additionally, high levels of IL‐13 receptors are expressed in the CRC area and precancerous polyps, indicating that MAIT cells may promote tumor development and metastasis through the IL‐13 pathway [246].

Tregs are a subset of T cells with significant immunosuppressive effects and are characterized by the expression of FOXP3, CD25, and CD4 as cell phenotype features. Within tumors, Treg cells accumulate extensively, comprising more than 50% of the total T‐cell population, and these cells suppress antitumor immune responses through various pathways [247]. MAIT cells can differentiate into a Treg phenotype in CRC, but the function of these cells is different from that of traditional Treg cells. In the presence of sustained TCR‐dependent stimulation, FOXP3 is abundantly expressed on the surface of CD4⁺ MAIT cells [248]. In addition, in the context of specific antigen induction and FOXP3 and CD25 expression on CD8⁺ MAIT cells, these CD8⁺CD25⁺FOXP3⁺ cells strongly inhibited T‐cell proliferation in vitro [249, 250]. Li et al. [251] comprehensively analyzed tumor‐infiltrating MAIT cells in CRC using flow cytometry and scRNA‐seq, identifying a FOXP3⁺CD4⁺ MAIT cell subset. The surface marker expression pattern of this subset resembles that of conventional Treg cells, but these MAIT cells also express TNF‐α, a function that is distinct from that of conventional Treg cells [251]. Furthermore, this study revealed high expression of TCR‐induced CD39 in tumor‐infiltrating MAIT cells. Previous studies have shown that the diminished reactivity of intestinal MAIT cells is closely associated with CD39 expression [27]. The observed increase in IL‐17 production and the expression of the inflammatory genes RSG1, CCL3, and CCL4 validated the protumor effects of CD39⁺ MAIT cells. In CRC, MAIT cells can also inhibit NK cell function, hindering tumor immune responses. Specifically, the ability of MAIT cells to express IL‐17A is enhanced and IL‐17A can suppress NK cell antitumor immunity [201].

The liver is the primary target organ for hematogenous metastasis in CRC, and colorectal cancer liver metastasis (CRLM) is a key focus and challenge in CRC treatment. To explore the immune surveillance role of MAIT cells in CRLM, Shaler et al. [204] examined the frequency and function of peripheral blood, healthy liver tissue, tumor margins, and tumor‐infiltrating MAIT cells in 21 CRLM patients, reporting that CD3ε⁺Vα7.2⁺CD161⁺⁺ or CD3ε⁺MR1 tetramer⁺ MAIT cells exist in both healthy and tumor‐affected liver tissues. Functional analysis revealed that the MAIT cells within the metastatic lesions exhibited widespread functional impairment, involving the TCR and cytokine receptor signaling pathways. Therefore, targeting each pathway individually may not be sufficient to overcome the functional defects of MAIT cells. Additionally, the study demonstrated that functional impairment is affected by physical location and is independent of whether the patient received chemotherapy before surgery. Notably, this cohort included only 3 CRLM patients, so this finding needs to be confirmed in a larger cohort [204].

Currently, the exact prognostic value of MAIT cells in CRC is relatively limited. In a study by Zabijak et al. [208], survival curves and multivariate analysis revealed that patients with greater recruitment of MAIT cells to tumors compared with adjacent healthy tissues exhibited poorer clinical outcomes. Yao et al. [232] survival analysis independently replicated these observations, indicating that MAIT cell infiltration in CRC is a negative prognostic factor for OS.

### 5.6 Esophageal Cancer

The global rankings for the incidence and mortality rates of EC are seventh and sixth, respectively [239]. EC includes two histological types: esophageal squamous cell carcinoma (ESCC) and esophageal adenocarcinoma (EAC). EAC occurs mainly in developed countries, whereas ESCC mainly occurs in developing countries such as Southeast Asia and Africa. Treatment options for EC are limited, and the prognosis is poor. Although treatment modalities are advancing, with targeted therapies and immune checkpoint inhibitors (ICIs) being more readily available, the overall 5‐year survival rate still remains at approximately 15–20%. There is an urgent need for new treatment strategies [252, 253]. The research team at Trinity College reported the characteristics of MAIT cells in ECs. They observed MAIT cells in the blood and tumors of patients with EC and Barrett's esophagus and reported the following findings: (1) Compared with those in healthy donors, the number of MAIT cells in the blood of cancer patients was lower, but the number of MAIT cells in esophageal tumors was greater than that in healthy tissues. (2) Unlike other types of T cells, the number of MAIT cells was not influenced by radiotherapy or chemotherapy treatment. (3) Healthy MAIT cells can kill EC cells in vitro, but this killing is reduced when fresh tumor biopsy fluid is present, indicating that factors from the tumor can inhibit MAIT cell cytotoxicity. (4) MAIT cells extracted from esophageal tumors exhibit high levels of markers associated with functional suppression. These findings suggest that esophageal tumors are able to prevent the killing of MAIT cells by conveying a “do not kill” signal via these inhibitory markers [209]. Furthermore, data from TCGA indicate a positive correlation between the presence of MAIT cells within EC tumors and a favorable prognosis [232]. Overall, the identification of new approaches to reverse the tumor‐suppressive ability of MAIT cells may provide a novel therapeutic strategy against EC.

### 5.7 Gastric Cancer

Although the incidence and mortality rates have declined in the past few decades, GC remains one of the major global health challenges. According to 2020 estimates from GLOBOCAN, GC caused approximately 800, 000 deaths (7.7% of all cancer deaths) and is the fourth leading cause of cancer‐related deaths in both sexes. Furthermore, significant regional differences in the incidence of GC are noted, with higher rates observed in East Asia [239, 254]. Although there is less research on MAIT cells in GC than in CRC, there are still some studies supporting this area of research. Compared with that in healthy controls, the frequency of circulating MAIT cells in the peripheral blood tends to decrease in GC patients [98]. The research team led by Wang et al. [255] collected and analyzed scRNA‐seq data from precancerous lesions, primary lesions, and metastatic tissues of gastric adenocarcinoma (GAC), revealing a significant increase in the number of MAIT cells in GAC metastatic lesions. This finding highlights the different TMEs between primary and metastatic lesions [255]. Research by Jiang and colleagues [256] indicated that normal MAIT cells exhibit an effector memory T‐cell phenotype and induce cytotoxic responses. Furthermore, MAIT cells in adjacent nontumor samples and primary tumor samples express Th1 cell cytokines, but this expression is almost absent in MAIT cells found in metastatic samples. In contrast, MAIT cells from metastatic samples highly express KLRG1, which is associated with T‐cell dysfunction. Although further experiments are needed for validation, the use of MAIT cells may provide a new approach for antitumor therapy in patients with GC [256]. *Helicobacter pylori* (*H. pylori*) has been classified as a specific carcinogen by the World Health Organization and the U.S. Department of Health and Human Services. MAIT cells in the human gastric mucosa are localized near *H. pylori*. In addition, animal experiments have shown that gastric MAIT cells exhibit an effector memory Tc1/Tc17 phenotype and are associated with accelerated gastritis. The hallmark features include increased recruitment of neutrophils, macrophages, DCs, eosinophils, and non‐MAIT T cells to the site of inflammation, as well as the onset of significant gastric atrophy [257]. Moreover, compared with those from healthy controls, MAIT cells from patients with *H. pylori* gastritis secreted increased levels of IL‐9. The percentage of IL‐9⁺ MAIT cells was positively correlated with the inflammatory cytokines IL‐6, TNF, IFN‐γ, and IL‐17; the chemokine CCL20; and the mucin‐related genes *MUC1*, *MUC5*, and *MUC6*. These findings suggest that IL‐9⁺ MAIT cells potentially regulate mucosal inflammation in *H. pylori*‐mediated gastritis. Detailed analysis of its mechanism revealed that the OX40–OX40L pathway promotes mucosal MAIT cell proliferation and IL‐9 production in *H. pylori*‐positive gastritis [258]. In addition to directly recognizing *H. pylori*, MAIT cells can also produce cytokines and exhibit cytotoxic activity by recognizing macrophages infected with *H. pylori*.

### 5.8 Oral Cancer

The oral and maxillofacial regions constitute the initial part of the digestive tract and are closely related to eating and nutritional status. The anatomical locations of primary tumors include the lips, tongue, upper and lower gums, floor of the mouth, hard palate, retromolar trigone, and anterior two‐thirds of the tongue, with the incidence and mortality rates gradually increasing. Global cancer statistics for 2020 revealed 377, 713 new cases of oral cancer (OC) and 177, 757 deaths, indicating that OC poses a serious threat to global human health [239]. There is currently no direct research on MAIT cells in OC. The current evidence only suggests that MAIT cells may serve as potential regulatory factors in the pathogenesis of OC and as future therapeutic targets.

### 5.9 Pancreatic Cancer

**FIGURE 5:**
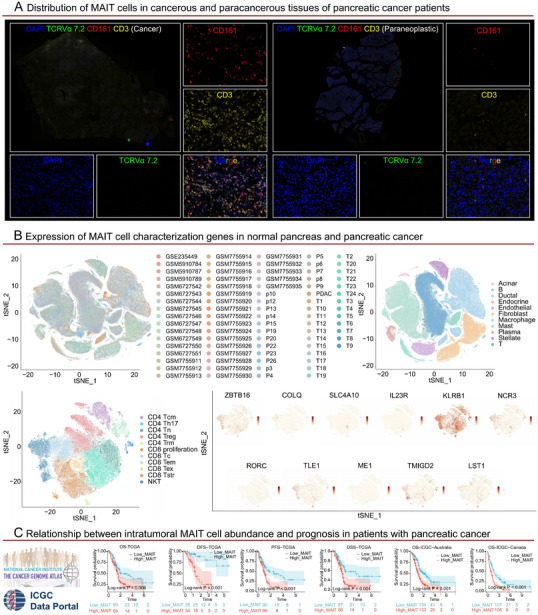
Distribution and prognosis of MAIT cells in pancreatic cancer. (A) Distribution of MAIT cells in cancerous and paracancerous tissues of PC patients analyzed by immunofluorescence. We defined CD3^+^TCRVα7.2^+^CD161^+^ cells as MAIT cells. Analysis revealed that the presence of MAIT cells was not detected in either cancerous or paracancerous tissues. (B) Single‐cell analysis of MAIT cell signature genes (SLC4A10, KLRB1, IL23R, NCR3, TMIGD2, LST1, COLQ, ME1, ZBTB16, RORC, TLE1) expression in cancerous and paracancerous tissues of PC patients (CRA001160, GSE155698, GSE197177, GSE205013, GSE217845, GSE235449, and GSE242230) [232, 370, 371, 372, 373, 374, 375, 376]. Overall, we did not identify MAIT cell subsets in PC patients, but we found expression of some of the MAIT cell signature genes in T cell subsets. This suggests that it is possible that a small number of MAIT cells may be present in PC, which may be due to migration of MAIT cells into the pancreas as a result of disruption of the local barrier function due to the tumor, intratumoral infection, and other reasons. (C) Relationship between intratumoral MAIT cell abundance and prognosis in PC patients (TCGA and International Cancer Genome Consortium [ICGC] databases [377, 378]). PC patients were classified into two groups based on MAIT cell signature genes, MAIT cell high expression and MAIT cell low expression (using multifactorial cox regression analysis to construct prognostic models). The results showed that patients with high MAIT cell expression had a poorer prognosis, both in the TCGA and ICGC databases.

Pancreatic cancer (PC) is currently recognized as one of the most malignant tumors. R0 surgical resection combined with adjuvant therapy is the only curative treatment option. However, owing to the occult nature of PC, difficulty in early diagnosis, and rapid progression, most patients are already beyond surgical indications at the time of diagnosis. In addition, PC is insensitive to antitumor drugs, leading to an extremely poor overall prognosis for patients. A global cancer statistics report for 2020 revealed 495, 773 new cases of PC and 466, 003 new deaths, with the number of new cases almost equal to the number of deaths [239]. The immune microenvironment of PC is characterized by an abnormally dense stroma, a low number of tumor cells, a low quantity of effector T cells, multiple immune suppressions, and so on. Essentially, it is a dense matrix formed due to excessive fibrosis caused by the active deposition of connective tissue [259]. Widespread fibrosis, a lack of blood vessels, immune infiltration, and a hypoxic stromal environment not only promote tumor growth and invasion but also increase resistance to antitumor drugs [260]. Research has shown that MAIT cells are undetectable in normal pancreatic tissue. In patients with pancreatitis, duodenal bacteria entering the pancreas through the pancreatic duct may cause MAIT cells to migrate to the pancreas and become activated. After the infection is cleared, MAIT cells may become depleted [261]. In patients with T1DM, the MAIT cell frequency not only increases with the development of diabetes but also increases with the production of GZMB and IFN‐γ, which may contribute to the destruction of beta cells [262, 263]. Similar results have also been reported in animal models of T1D (particularly in NOD mice). Compared with those in control C57BL/6 mice, MAIT cells in these mice are activated and produce higher levels of cytokines in the pancreatic lymph nodes, islets, and intestines. The frequency of MAIT cells increases as inflammation progresses in NOD mice [153]. In type 2 diabetes mellitus (T2DM), circulating levels of MAIT cells are significantly decreased. In some severely obese patients, MAIT cells are not detectable at all, and the frequency of MAIT cells is negatively correlated with body mass index and insulin sensitivity. Compared with healthy controls, MAIT cells in the blood present a significantly activated proinflammatory Th17 phenotype. After stimulation, T2DM patients produce increased levels of GZMB, IL‐17, and TNF‐α [264, 265]. At present, there are no reports on the relationship between MAIT cells and PC (Figure 5).

In general, MAIT cells exhibit significant functional plasticity in the TME. They directly kill tumor cells by secreting IFN‐γ, TNF‐α, and cytotoxic molecules, while also regulating innate immune responses of cells such as NK cells and macrophages. However, under chronic stimulation, hypoxia, and the action of inhibitory factors, MAIT cells are prone to exhaustion, shift toward secreting protumor factors such as IL‐17, and may even differentiate into regulatory subsets with immunosuppressive functions‐ultimately promoting tumor progression. This dual role is tightly regulated by tumor type, TME signals, and intercellular interactions. Targeting the functional polarization of MAIT cells provides a novel strategy for enhancing antitumor immunity, and its potential as a prognostic biomarker and therapeutic target urgently requires further exploration.

### 5.10 Crosstalk Between MAIT Cells and Other Cells in Tumors

**FIGURE 6:**
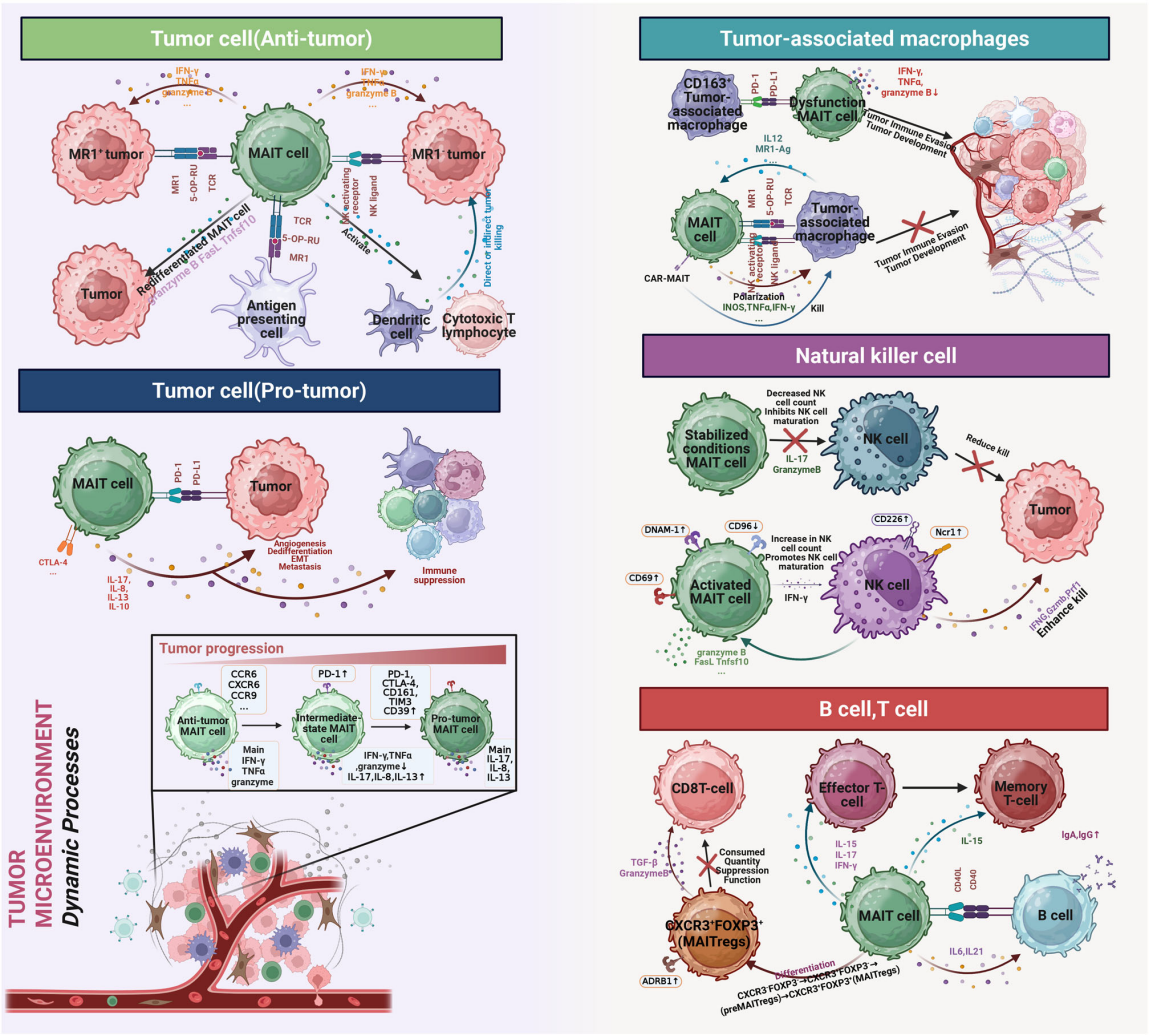
Cross‐talk between MAIT cells and other cells in tumors. The picture is created in https://www.biorender.com/.

The TME refers to the internal environment composed of tumor cells, locally infiltrating immune cells, stromal cells, and so on, and is the material basis for tumor cell survival. The immune cells include lymphoid cells (T cells, B cells, and NK cells) and myeloid cells (macrophages, DCs, monocytes, and neutrophils), whereas the stromal cells include fibroblasts and endothelial cells. In addition to directly acting on tumor cells, MAIT cells can also interact with various immune cells, generating various indirect antitumor or protumor immune responses (Figure 6).

#### 5.10.1 Natural Kill Cells

NK cells exhibit direct cytotoxic functions in innate immunity. They express a variety of activating and inhibitory receptors on the cell surface to monitor malignant cells. The anticancer effects of NK cells involve two main aspects. The first is direct killing of tumor cells by the release of perforin and granzymes or through death receptors. The second is their role as regulatory cells in the immune system by secreting cytokines and chemokines, activating the killing effect of T cells, and so on [266, 267]. Although MAIT cells and NK cells differ in their activities and recognition mechanisms, they participate in different aspects of immune responses. MAIT cells express activating NK receptors, such as NKG2D and DNAM‐1, which recognize NK‐related stress ligands present on tumor cells, thereby generating NK‐mediated cytotoxicity [8]. Petley and others [268] studied MAIT cell function in mouse tumor models (B16F10 and E0771), revealing that MAIT cells mediate antitumor immune responses by modulating NK cell activity in an IFN‐γ‐dependent manner. This study also provides evidence for the similar functionality of MAIT cells in human cancer [268]. In addition, MAIT cells in the colon are activated by tumor cells presented through MR1, thereby negatively impacting the antitumor responses of NK cells and CD8⁺ T cells [201].

#### 5.10.2 Macrophages

TAMs are immune cells in the TME that are present mainly in the tumor stroma and mediate inflammatory responses. Typically, TAMs can polarize into different phenotypes of M1 and M2 macrophages after stimulation by various factors. In the early stages of tumors, M1 macrophages in TAMs play a predominant role in promoting inflammation and antitumor effects. However, as tumors progress, TAMs gradually polarize toward M2 macrophages, which play a predominant role in suppressing inflammation and promoting tumor growth [269]. Macrophages express high levels of MR1 [33, 72, 270], especially M2‐polarized macrophages and endogenous TAMs, in cancer patients, providing a foundation for the anti‐TAM response of MAIT cells. Specifically, MAIT cells target M2 macrophages directly through innate NK activation receptors. Additionally, 5‐OP‐RU induces TCR activation and further enhances the anti‐TAM capabilities of MAIT cells. On the basis of these findings, the next research directions involve CAR‐modified MAIT cells, such as MAIT cells expressing a CAR for mediator proteins, which exhibit cytotoxicity against MR1‐positive TAMs and enhanced antitumor abilities [23].

Furthermore, the interaction between MAIT cells and macrophages is evident in various gastrointestinal diseases. In MR1^−/−^ mice, the absence of GM‐CSF secretion by MAIT cells results in a hindered process of macrophage maturation [88]. In vitro, activated MAIT cells can induce monocyte/macrophage differentiation into the M2 phenotype. In a nonalcoholic steatohepatitis (NASH) mouse model induced by a methionine‐ and choline‐deficient (MCD) diet, mice lacking MAIT cells exhibited more severe hepatic steatosis and inflammation after MCD diet feeding. Additionally, the proportion of CD11c proinflammatory M1 macrophages increased relative to that of CD206 M2 macrophages in these mice, indicating that MAIT cells protect nonalcoholic fatty liver disease (NAFLD) patients from inflammation by producing regulatory cytokines and inducing anti‐inflammatory macrophage polarization [271]. In liver biopsy samples from patients with liver fibrosis and cirrhosis, the inhibition of MAIT cell activation reduces the expression of inflammation‐ and fibrosis‐related genes. In mouse models, the use of antagonists or antibodies to inhibit MAIT cell activation can restrict the progression of liver fibrosis following chronic toxicity or NASH‐induced liver injury and even promote the regression of liver fibrosis. Combined RNA‐seq and coculture experiments suggest that the inhibition of MAIT cell activation promotes the transformation of Ly6C^hi^MoMac into Ly6C^lo^MoMac and enhances the autophagic phenotype of both subgroups, thereby inducing the regression of liver fibrosis. These findings indicate that targeting the transformation of liver macrophage phenotypes through MAIT cell regulation may be a potential strategy for treating liver fibrosis [272].

#### 5.10.3 B Cells

To date, research related to tumor immunotherapy has focused mainly on T cells, but increasing evidence suggests that tumor‐infiltrating B cells and plasma cells (referred to as tumor‐infiltrating B lymphocytes [TIL‐Bs]) play crucial synergistic roles in tumor control. In many cancers, TIL‐Bs have shown significant predictive and prognostic significance in the context of standard treatments and ICB, providing new therapeutic opportunities for treating malignant tumors [273]. Research on the interaction between MAIT cells and B cells in the TME has not been reported, but their interaction is very close in other diseases. B cells are among the APCs for MAIT cells and highly express MR1 on their surface. After being infected by bacteria, B cells directly activate MAIT cells; secrete IFN‐γ, TNF‐α, and IL‐17; and upregulate CD69 expression [274, 275]. In vitro experiments have shown that MAIT cells increase the production of antibodies by B cells through the CD40L‒CD40 interaction and the TCR pathway [276]. Alternatively, plasma cell differentiation and antibody secretion from memory B cells is induced in a manner dependent on MR1 (cell factors such as IL‐6, IL‐10, and IL‐21) [277]. In many autoimmune diseases, the enhanced IL‐17 secretion capacity of MAIT cells promotes the autoimmune process [278]. This finding may be due to the synergistic effect of IL‐17 with B cell‐activating factor, which promotes the emergence of autoreactive B cells and autoantibody production [279, 280].

MM is a malignant tumor characterized by the abnormal proliferation of plasma cells. Compared with those in healthy controls, the numbers of MAIT cells in the circulation and bone marrow are lower in MM patients [7, 213]. MAIT cells from newly diagnosed MM patients show reduced IFN‐γ production and CD27 expression and decreased cytotoxic ability. In contrast, MAIT cells from healthy donors can kill MM cells in vitro in a dynamic manner similar to that of NK cells. These data highlight the potential therapeutic opportunity of manipulating MAIT cells as a form of treating MM [7].

#### 5.10.4 Neutrophils

Neutrophils participate in different stages of tumor initiation and progression, including tumor development, proliferation, and metastasis. The population of neutrophils in the TME exhibits heterogeneous phenotypes and diverse functions, playing dual roles as either protumoral (N2) or antitumoral (N1) factors [281]. MAIT cells are key regulators of innate and adaptive immune responses mediated by neutrophils in response to various pathogens. Following microbial infections, MAIT cells promote longer survival of neutrophils and their differentiation into cells resembling APCs (which express CD64, CD83, HLADR, CD54, CD40, HLAA, HLAB, and HLAC). Activated neutrophils can efficiently process exogenous antigens and activate CD4⁺ and CD8⁺ T cells [282]. In contrast, on the basis of in vitro experimental results, Schneider et al. [283] proposed a negative feedback model. Specifically, MAIT cells are rapidly activated within minutes after inflammation occurs, which aids in the recruitment of neutrophils, but neutrophils subsequently inhibit MAIT cell activation. Although moderately activated MAIT cells may support the survival of neutrophils, strongly activated MAIT cells induce neutrophil apoptosis in a TNF‐α‐dependent manner. Therefore, the balance between MAIT cells and neutrophils may be an important factor in providing effective and controlled immune responses [283].

In conclusion, in the TME and related diseases, the interactions between MAIT cells and other immune cells are bidirectional and complex. MAIT cells can mediate antitumor or protumor effects by regulating the functions of these cells, and some of these interaction mechanisms provide targets for disease treatment. In the future, in‐depth analysis of their regulatory networks is needed to advance the development of MAIT cell‐related immunotherapies.

## 6 MAIT Cell‐Targeted Therapeutic Strategy

As mentioned earlier, MAIT cells, a subset of T cells with unique innate‐like immune properties, play a critical immunomodulatory role in various pathological processes, including infectious diseases, autoimmune diseases, metabolic diseases, chronic inflammation, and cancer. Their core advantages stem from their conserved biological characteristics and functional plasticity, which can be summarized as follows: (1) widespread tissue distribution—they are enriched in mucosal tissues, peripheral blood, skin, and other systemic tissues, enabling participation in local pathological changes and systemic immune responses [27, 121]; (2) dual activation pathways—these cells are rapidly activated through MR1‐dependent and MR1‐independent pathways, initiating responses earlier than conventional T cells [72, 86]; (3) diverse effector functions—upon activation, they secrete cytokines such as IFN‐γ and IL‐17 to regulate immunity and tissue repair, release GZMB, and express NKG2D to mediate cytotoxicity for clearing infected/tumor cells [24]; (4) metabolic plasticity—these cells are capable of dynamic metabolic reprogramming to adapt to stress in different microenvironments [284, 285]; (5) microbiota interactivity—these cells depend on microbiota‐derived metabolic antigens for development while shaping microbiota composition, making them suitable for intervention in microbiota dysbiosis‐related diseases [286]; (6) low immune rejection risk—these cells are restricted by nonpolymorphic MR1, barely inducing GVHD in allogeneic therapy and are thus suitable for “off‐the‐shelf” cell therapy [225]; (7) therapeutic tolerance—these cells highly express multidrug resistance proteins, conferring tolerance to certain chemotherapeutic agents, and can be reactivated for antitumor functions via ICB [33, 287]; (8) precise regulatability—functional phenotypes of these cells can be modulated through MR1 ligands, cytokines, metabolic intervention, or genetic engineering (e.g., CAR modification) [195]. Based on these advantages, MAIT cells have emerged as important targets for immunotherapy. In this section, in the context of existing findings, we systematically discuss various targeted therapeutic strategies for MAIT cells, as well as their application prospects and challenges in multiple diseases.

### 6.1 Activation Strategies Based on MR1 Ligands

The functional regulation of MAIT cells via ligands presented by the nonpolymorphic MR1 has emerged as a cutting‐edge immunotherapeutic strategy for the treatment of infections, autoimmune diseases, tumors, and other conditions. Its core mechanism involves the use of small‐molecule metabolites or drug derivatives bound to MR1 to precisely modulate the activation status, cytokine profiles, and effector functions of MAIT cells through agonistic or inhibitory approaches. Meanwhile, the functional plasticity of MAIT cells in the tissue microenvironment and their dependence on pathological contexts must also be carefully considered [17, 19, 288]. The antigen‐binding pocket of MR1 exhibits significant structural plasticity, enabling it to accommodate a variety of metabolites derived from B vitamins. For instance, 5‐OP‐RU and RL‐6, 7‐diMe, which are key intermediates in the bacterial riboflavin synthesis pathway, act as classical agonistic ligands that effectively activate MAIT cells by occupying the A′ pocket. By contrast, the folate metabolite 6‐FP exerts inhibitory effects, suggesting that the structure–activity relationship of ligands holds bidirectional regulatory potential over MAIT cell responses [19, 20]. Additionally, nonvitamin ligands (e.g., diclofenac metabolites) can weakly activate certain MAIT TCRs, whereas 3‐formylsalicylic acid inhibits MAIT cell responses through competitive bindings, further expanding the pharmacological regulatory scope of MR1 ligands [288]. In the context of infection, MAIT cells are rapidly activated after their TCRs recognize bacterial metabolites presented by MR1 on the surface of infected cells. They then kill bacteria‐infected epithelial cells and macrophages, playing a critical role in controlling intracellular pathogens such as *K. pneumoniae* and *Francisella* [17, 51]. If local bacterial metabolism is insufficient to activate MAIT cells during infection, exogenous supplementation with high‐affinity agonistic ligands (e.g., 5‐OP‐RU) can significantly enhance MAIT cell proliferation and effector functions. For example, in a mouse model of *Legionella* infection, priming the in vivo MAIT cell immune response with 5‐OP‐RU combined with TLR9/2 agonists enhanced protection [132]. Conversely, in chronic infections such as *H. pylori*‐induced gastritis, sustained antigen exposure leads to MAIT cell exhaustion. In such cases, careful design of ligand administration strategies is required to restore their antibacterial capacity [142, 257]. With respect to autoimmune conditions, aberrant MAIT cell activation often drives tissue inflammation. For instance, in type 1 diabetes, MAIT cells mediate pancreatic β‐cell damage by secreting IFN‐γ and GZMB. This suggests that blocking TCR signaling with MR1‐inhibitory ligands (e.g., acetyl‐6‐FP) can alleviate inflammatory responses, thereby delaying disease progression [153]. In tumor immunoregulation, MAIT cell function exhibits marked context dependence. In the TME of colorectal cancer, hepatocellular carcinoma, and other malignancies, MAIT cells exhibit high expression levels of inhibitory receptors, such as PD‐1 and CTLA‐4, and tend to secrete IL‐17, which promotes angiogenesis and metastasis. Under these conditions, blocking MR1 with inhibitory ligands (e.g., 6‐FP or acetyl‐6‐FP) can effectively reverse the protumor phenotype of MAIT cells. Moreover, in melanoma models, treatment with MR1‐blocking antibodies has been shown to abrogate the inhibitory effect of MAIT cells on NK cells [200, 201, 289]. However, ligand design must avoid interfering with the antitumor functions of other MR1‐restricted T‐cell subsets. Therefore, an ideal strategy would involve developing ligand variants that target MAIT cell‐specific TCR signaling patterns or selectively enhancing MR1‐mediated presentation of tumor‐associated self‐antigens through metabolic intervention (e.g., modulation of the riboflavin metabolism pathway in tumor cells), thereby specifically activating antitumor responses [288, 290, 291]. Notably, the exhausted state of MAIT cells in tumors may also be reversed by combining low‐intensity TCR agonists (e.g., diclofenac metabolites) with ICIs, although strict evaluation of cytokine output is necessary to avoid IL‐17‐related risks [200, 288]. Furthermore, the pharmacokinetic properties of ligands require urgent optimization—for example, developing long‐acting agonists with tissue‐retention capabilities or adopting local drug‐delivery systems—to achieve sustained immunomodulation at lesion sites while minimizing the risk of systemic exposure. Despite the broad application prospects of MAIT cell regulation based on MR1 ligands, significant challenges remain. The chemical properties of MR1‐presented self‐antigens in tumors have not been elucidated, limiting the design of personalized ligands; long‐term MAIT cell inhibition may impair mucosal barrier defense; and interindividual diversity in TCR repertoires affects ligand responsiveness [153, 290, 292]. Future research should focus on developing ligand‐specific or mutant MR1 molecules to achieve precise regulation. Further, single‐cell technologies can be combined with metabolomics to thoroughly characterize the types and dynamic changes of MR1 ligands in different disease contexts, thereby advancing the development of personalized immunotherapeutic strategies targeting the MR1–MAIT axis.

### 6.2 Regulation of Cytokines and Costimulatory Signals

Interventional strategies targeting cytokines and costimulatory signals to modulate MAIT cell function are based on the unique activation and plasticity mechanisms of MAIT cells. Although MAIT cells can be activated via their TCRs that recognize microbe‐derived riboflavin metabolites presented by MR1, substantial evidence indicates that the full activation and polarization of their effector functions are highly dependent on costimulatory signals provided by cytokines, which could even be completely independent of TCRs. This provides multiple actionable targets for precise immunological intervention [86, 293]. Particularly in pathological contexts, MAIT cells exhibit remarkable functional plasticity, and their ultimate phenotype—whether pro‐ or anti‐inflammatory/repair‐oriented—is largely dictated by the combination of cytokines in the local microenvironment [294, 295, 296]. For example, the synergistic action of IL‐12 and IL‐18 potently induces MAIT cells to produce high levels of IFN‐γ and enhances their granzyme‐mediated cytotoxicity. This process occurs independently of TCRs, highlighting the innate‐like lymphocyte characteristics of MAIT cells. It has been confirmed as a key activation pathway for this cell population in various infection models involving bacteria (e.g., *M. tuberculosis* and *Streptococcus pneumoniae*) and viruses (e.g., influenza and dengue viruses) [72, 76, 293, 297, 298, 299]. Other cytokines, such as IL‐7 and IL‐15, are not only critical for the homeostatic survival and proliferation of MAIT cells (via high expression levels of IL‐7R and IL‐15R) but also “arm” the cells under specific conditions. Pretreatment with IL‐7 significantly enhances the ability of MAIT cells to produce IL‐17A and IFN‐γ following TCR activation and restores their impaired cytotoxic function in HIV infection [34, 71, 85]; by contrast, IL‐15 strongly activates the cytotoxicity of hepatic MAIT cells in a completely MR1‐independent manner by upregulating NKG2D and GZMB expression [104]. Type I IFNs have also been shown to be effective modulators of MAIT cells—they can either activate the killing program of MAIT cells independently or synergize with TCR signals to significantly amplify the secretion of IFN‐γ and TNF‐α. This is crucial for antibacterial (e.g., *K. pneumoniae*) and antiviral (e.g., HCV, IAV) responses [72, 300, 301]. Furthermore, cytokines can drive the functional polarization of MAIT cells—stimulation with IL‐23 tends to induce a regulatory phenotype with high coexpression of cytotoxic molecules and IL‐10, a process dependent on the transcription factor BATF, whereas IL‐18 preferentially promotes the production of proinflammatory IL‐17 [295, 296]. Notably, a complex crosstalk and integration network exists among cytokine signals. For instance, in PBMCs cultures, IL‐15 indirectly activates MAIT cells by inducing monocytes to produce IL‐18, whereas IL‐12 can induce c‐MAF‐dependent IL‐10 secretion, demonstrating the hierarchical and context‐dependent nature of cytokine network regulation [296, 302]. The regulatory role of costimulatory signals in MAIT cell responses also cannot be ignored—CD28 costimulation can enhance TCR signaling, and inducible T cell costimulator signaling has been confirmed to be essential for the development of the MAIT17 subset in mice. This suggests that a strategy of targeting costimulatory pathways may be applied to guide the differentiation or functional bias of MAIT cell subsets [86, 303]. The development of interventional strategies is based on the mechanisms listed herewith. In antibacterial immunity, the combined use of MR1 ligands and IL‐23 serves as an effective vaccine strategy, significantly enhancing protection against bacterial lung infections [270]; in chronic viral infections, exogenous IL‐7 therapy can reverse MAIT cell exhaustion and restore their cytotoxic function, with its efficacy even correlating with IL7RA genotypes [71, 85, 304]; in critically ill patients with COVID‐19, severe impairment of MAIT cell function has been observed, which is associated with elevated IL‐18 levels and inhibition by monocyte‐derived IL‐10, suggesting that anti‐IL‐18 or IL‐10R antagonists may have therapeutic potential [163, 305]; in autoimmune or inflammatory diseases, modulating cytokines that drive pathological MAIT cell responses or leveraging the IL‐10‐inducing potential of MAIT cells may alleviate disease [295, 296, 306]. In summary, cytokines and costimulatory signals dynamically integrate to regulate the activation, proliferation, differentiation, and effector functions of MAIT cells. Interventional strategies targeting these signaling pathways demonstrate clear clinical translation potential for infectious diseases, immune disorders, and tumors. Future research should further dissect the mechanisms of synergy between cytokine combinations and costimulatory signals in specific pathological microenvironments, as well as elucidate the underlying transcriptional regulatory networks and epigenetic modifications to advance the precision and clinical translation of MAIT cell‐targeted immunotherapeutic strategies.

### 6.3 Cell Therapy and Genetic Engineering Strategies

Current research progress in the field of cancer immunotherapy has positioned MAIT cells as highly promising “off‐the‐shelf” immunotherapeutic carriers owing to their unique biological characteristics—including the expression of an invariant TCR α chain restricted by the highly conserved MHC class I‐like molecule MR1, the ability to recognize microbe‐derived riboflavin metabolic derivatives, and strong cytotoxicity as well as the capacity to home to mucosal barrier tissues [1, 2, 307, 308]. Of particular importance is that MAIT cells do not recognize allogeneic MHC molecules or self‐antigens, thus presenting minimal risk for GVHD in allogeneic transplantation [225, 309].

To overcome the challenge of limited in vitro expansion caused by the low proportion of MAIT cells in peripheral blood, researchers have developed two main strategies. First, enrich MAIT cells derived from donor PBMCs that are positive for the 5‐OP‐RU/MR1 tetramer via immunomagnetic bead sorting. Then, achieve a 60–200‐fold in vitro expansion by combining these enriched MAIT cells with either irradiated PBMC feeder cells or latex bead‐based artificial APCs coated with MR1 tetramers/anti‐CD28 antibodies, in the presence of IL‐2 or IL‐15 [101, 310, 311]. Second, genetically engineer HSCs or induced pluripotent stem cells (iPSCs) by introducing MAIT TCR genes through lentiviral vectors. Subsequently, generate scalable HSC‐derived MAIT (HSC–MAIT) cells or iPSC‐reprogrammed MAIT‐like (reMAIT) cells using the OP9–DL1 coculture system or artificial thymic organoids [26, 312, 313]. Building on this foundation, CAR‐engineered modification of MAIT cells can endow them with the ability to target tumor‐specific antigens. For instance, mesothelin‐targeting CAR–MAIT cells effectively kill tumor cells in 3D organoid models. Moreover, they can recognize TAMs via the expression of NK‐activating receptors such as NKG2D and DNAM‐1, thereby reversing the inhibitory effect of the immunosuppressive microenvironment on conventional CAR–T cells [23]. Similarly, CD19‐targeting CAR–MAIT cells exhibit antilymphoma activity in immunodeficient mouse models without inducing GVHD [314].

The antitumor mechanism of CAR–MAIT cells exhibits triple synergy: (1) CAR‐dependent pathway—recognizing tumor surface antigens through the CAR; (2) TCR‐dependent pathway—recognizing 5‐OP‐RU presented by MR1 on the surface of tumor cells or APCs (some tumor cells can upregulate MR1 expression under stimulation by microbial metabolites); (3) NK‐like pathway—recognizing stress ligands (e.g., MIC‐A/B) via NK‐activating receptors (such as NKG2D and DNAM‐1). This pathway enables CAR–MAIT cells to maintain cytotoxicity even against MR1‐negative tumors, thus effectively combating tumor antigen escape [8, 23, 24]. In the treatment of solid tumors, CAR–MAIT cells demonstrate unique advantages. For example, HER2‐targeting CAR–MAIT cells infiltrate tumor tissues by expressing high levels of homing receptors, such as CXCR6 and CCR6, in breast cancer models. They also secrete IFN‐γ to activate NK cells and CTLs while inhibiting the immunosuppressive function of MDSCs [24, 268]. In ovarian cancer models, mesothelin‐targeting CAR–MAIT cells maintain cytotoxicity even in a microenvironment with immunosuppressive M2 macrophages, which is a significantly superior characteristic compared with conventional CAR–T cells with impaired function [23].

Despite the broad prospects of CAR–MAIT therapy, several challenges remain to be addressed. For instance, the risk of cytokine release syndrome can be controlled by introducing suicide genes (e.g., *iCasp9*) or conducting dose‐escalation trials [315]. The insufficient infiltration efficiency into solid tumors can be improved by optimizing the expression of homing receptors or in combination with tumor mRNA vaccines to enhance targeting [316]. Furthermore, the production of universal CAR–MAIT cells using the iPSC platform is expected to further reduce costs and promote clinical translation [26, 313, 317]. In conclusion, with their inherent mucosal homing ability, multiple tumor‐killing mechanisms, low GVDH risk, and engineerable properties, CAR–MAIT cells provide a breakthrough strategy for “off‐the‐shelf” immunotherapy for hematological malignancies and solid tumors.

### 6.4 Microbiota–Immune Axis Intervention Strategies

Recent studies have shown that the microbiota drives the development and functional differentiation of MAIT cells by providing riboflavin‐derived antigens, while MAIT cells in turn shape the composition of microbial communities through cytokine secretion and cytotoxic activity [318]. The imbalance of this symbiotic relationship is closely associated with various inflammatory diseases; therefore, targeting the microbiota–MAIT cell axis is a highly promising therapeutic strategy. Dysbiosis and MAIT cell dysfunction form a vicious cycle in multiple diseases. In IBD, the frequency of peripheral MAIT cells decreases, whereas they are enriched in the inflamed intestinal mucosa—CD is characterized by a skewed secretion of IL‐17, whereas UC shows high expression of CD69 and increased IL‐22 secretion [29, 306]. Animal models (oxazolone‐induced colitis) suggest that MAIT cells may exacerbate inflammation via IL‐17 [319]; however, but human studies have revealed that IL‐17 in active CD is mainly derived from conventional Tc17 cells [320]. This discrepancy may stem from the imbalance of the “microbiota–barrier–immunity triangle”—intestinal dysbiosis (abnormal Firmicutes/Bacteroidetes ratio) impairs barrier integrity, leading to translocation of microbial products that activate MAIT cells. High antigen load, in synergy with proinflammatory cytokines (IL‐12/IL‐18), drives the acquisition of an inflammatory phenotype [27, 286]. In patients with obesity, circulating MAIT cells are reduced, whereas visceral adipose MAIT cells are increased and exhibit an activated IL‐17⁺ GZMB⁺ phenotype [264, 265]. High‐fat diet‐induced dysbiosis increases MAIT cell apoptosis, and surviving cells promote the polarization of M1 macrophages in adipose tissue and insulin resistance via IL‐17 [321]. In NAFLD, MAIT cells migrate to the liver, upregulate PD‐1, and secrete IL‐4 to induce M2 macrophage differentiation [271]; however, during the fibrotic stage, they localize to fibrous septa and promote the proliferation of hepatic stellate cells through an MR1‐dependent contact mechanism [322]. Patients with alcoholic liver disease show impaired MAIT cell function and elevated plasma endotoxin levels, and fecal microbiota transplantation (FMT) can recapitulate their abnormal phenotype [323]. In patients with asthma and COPD, the frequency of airway MAIT cells is significantly reduced, which is negatively correlated with the dose of inhaled corticosteroids (ICS) [324, 325]. In severe asthma, the microbiota shifts toward Proteobacteria (e.g., *Haemophilus influenzae*), and ICS further impairs the IFN‐γ response of MAIT cells to pathogens [325, 326]. Residual MAIT cells exhibit an IL‐17/TNF‐skewed activated phenotype (CD69⁺ PD‐1⁺), which may be driven by local elevation of IL‐7 [327]. In patients with COPD, MAIT cells are enriched in lung tissue with increased IL‐17 secretion; reduced circulating frequency is associated with elevated serum C‐reactive protein levels and increased risk of acute exacerbations [328, 329]. MAIT cell depletion impairs antimicrobial defense, and the synergy between dysbiosis (decreased *Bacteroidetes*/expanded *Proteobacteria*) and the inflammatory microenvironment exacerbates the disease [330]. Chronic HIV/HCV infection leads to persistent depletion of circulating MAIT cells [30, 331], accompanied by intestinal dysbiosis (expanded *Bacteroidetes/expanded Proteobacteria*) and elevated plasma endotoxin [332]; however, pulmonary MAIT cells retain tissue repair transcriptional signatures during HIV infection [149]. After HSCT, MAIT cell reconstitution depends on donor microbiota diversity; the abundance of Bacteroidetes is positively correlated with MAIT cell frequency, and the genus *Blautia* can predict success of reconstitution [224, 225, 333].

Intervention strategies targeting the microbiota–MAIT cell axis focus on three key dimensions: (1) Direct modulation of receptor signaling—local administration of stable 5‐OP‐RU analogs can activate the antimicrobial and tissue repair programs of MAIT cells (e.g., topical application on mouse skin accelerates wound healing) [334]; intratracheal delivery induces MAIT cell‐dependent bacterial clearance in chronic TB infection [335]. Oral administration of the MR1 antagonist Ac‐6‐FP inhibits IL‐17 secretion by MAIT cells in obesity models, alleviates enteritis, and increases the abundance of Bacteroidetes [321]. (2) Remodeling of microbial communities—colonization with riboflavin‐producing bacteria (*Bacteroidetes*/*Proteobacteria*) enhances MAIT cell activation [318]. Inulin‐encapsulated *Bifidobacterium* extends intestinal retention (>96 h) to promote the proliferation of riboflavin‐producing bacteria, and genetic engineering to construct high riboflavin‐producing engineered bacteria (e.g., *Bifidobacterium* expressing RibD enzyme) represents a direction for precise intervention [82]. FMT promotes MAIT cell reconstitution and reduces the risk of GVHD after HSCT by restoring microbiota diversity, indirectly enhancing riboflavin synthesis [159, 225, 226]. Antibiotics such as rifaximin reduce pathogenic bacteria (*Klebsiella*/*Streptococcus*) and increase probiotics (Bacteroidetes) in patients with liver cirrhosis, thereby slowing MAIT cell depletion [336]. (3) Combined repair of the barrier and immune microenvironment—MAIT cell functional output is regulated by epithelial barrier integrity; when the barrier is intact, commensal bacteria provide TCR signals to drive tissue repair programs (expressing VEGFB/PDGFB/TGFBI) [27, 337]. When the barrier is damaged, pathogen‐associated molecular patterns and damage‐associated molecular patterns synergistically induce inflammatory responses. Therefore, combined targeting of MR1 and costimulatory signals may optimize therapeutic efficacy, and a sequential strategy (using antagonists to reduce inflammation followed by agonists to promote repair) is a potential therapeutic approach [337].

Current challenges focus on colonization stability (oral probiotics are easily destroyed by gastric acid, and the survival rate of anaerobic bacteria in lyophilized FMT is low; calcium tungstate microgel encapsulation technology improves implantation efficiency through “niche occupation”) [318], individual heterogeneity, and spatiotemporal complexity (MAIT cell function is competitively regulated by the tissue microenvironment, microbiota metabolic dynamics, and coexisting immune cells such as γδT/iNKT cells) [334, 338]. Future research needs to combine single‐cell multiomics and spatial transcriptomics to dissect the dynamic network of microbiota–MAIT cell interactions at different disease stages, develop MR1 ligand‐targeted delivery systems (e.g., nanocarriers), and explore MAIT cells as response biomarkers for microbiota‐directed therapy. By integrating microbiomics, synthetic biology, and immune engineering, targeting the microbiota–MAIT cell interactions at different disease stages, develop MR1 ligand–MAIT cell axis is expected to provide a next‐generation precision treatment strategy for chronic inflammatory diseases.

### 6.5 MAIT Vaccine Development Strategies

MAIT cell‐based vaccine development strategies are emerging as a crucial research direction for enhancing anti‐infective immunity. These vaccine development strategies are based on leveraging MR1‐dependent ability of MAIT cells to recognize antigens derived from the microbial riboflavin pathway, as well as their MR1‐independent activation property via cytokines, thereby designing vaccine platforms that can specifically activate and expand MAIT cells [20]. For instance, traditional vaccine strains are genetically engineered to enhance the production of MAIT cell ligands. In TB research, Bacillus Calmette–Guérin‐overexpressing riboflavin pathway genes can increase the secretion of antigens such as 5‐OP‐RU. Although vaccination in mice did not significantly improve pathogen clearance, a moderate reduction in bacterial loads in the lungs and spleen was observed, confirming the feasibility of MAIT cell activation [339]. Similar strategies have achieved more significant effects in bacterial vaccines. For example, an attenuated *Shigella* vaccine has been reported to activate MAIT cells, promoting MAIT‐mediated lysis of infected cells and induction of B‐cell responses [17]. Further, a *Salmonella* vaccine strain has been reported to expand two subsets of MAIT cells, which confer protection against *S. pneumoniae* and influenza virus [171]. In addition, direct integration of MR1 ligands as novel adjuvants is another key approach—adding 5‐OP‐RU to influenza vaccines enhances the proinflammatory response of MAIT cells and effectively controls viral replication. This effect is maintained in both young and elderly individuals, suggesting its potential to address immunosenescence [340, 341]. Viral vector vaccines also exhibit synergistic effects. After vaccination with chimpanzee adenovirus vectors (e.g., ChAdOx1), MAIT cell activation upregulates cytokine signaling pathways, thereby enhancing CD8⁺ T‐cell immunity—a mechanism that has been verified in the development of Ebola and COVID‐19 vaccines [342, 343, 344]. Notably, MAIT cells show a robust response to microbe‐based vaccines. For example, in a challenge with live typhoid *Salmonella* vaccine, circulating MAIT cells rapidly decreased (within 4 days); however, their frequency recovered after antibiotic treatment, indicating a dynamically reversible expansion profile [345]. Furthermore, adenovirus vectors can induce MAIT cell clonal expansion and TCR repertoire remodeling, which further strengthens long‐term immune memory [344]. However, TB vaccine development faces unique challenges. Although rhesus macaque models confirm that MAIT cells migrate to lesion sites during the early stage of TB infection and initiate Th1 responses [346], preactivation of MAIT cells with 5‐OP‐RU fails to limit TB infection in mice or primates and even triggers cell exhaustion owing to PD‐1 upregulation [346, 347]. This may be a result of *M. tuberculosis‐*specific immune evasion mechanisms (e.g., blocking γc/IL‐2Rβ receptors and inhibiting IFN‐γ production via PD‐1 signaling) [348, 349]. To overcome this limitation, an emerging cell therapy approach—reMAIT cells—was applied, wherein the cells successfully migrated to infected tissues in mouse models, reducing *M. abscessus* loads by 40–50% through the release of granulysin; furthermore, these cells showed the ability to mature and expand in vivo [26]. Combining reMAIT cells with preactivation using TB‐specific antigens may help avoid exhaustion. Optimization strategies also need to consider host–pathogen metabolic interactions—ligands produced by bacteria in the stationary growth phase and anaerobic environments exhibit the strongest MAIT cell activation [350], whereas *M. tuberculosis* may inhibit MAIT function by generating antagonists [351]. Thus, vaccine design must regulate ligand balance. In conclusion, MAIT cell‐targeted vaccines need to integrate ligand engineering, adjuvant optimization, vector selection, and metabolic microenvironment regulation, while addressing pathogen‐specific immunosuppressive mechanisms. Personalized therapies (e.g., reMAIT adoptive transfer) may provide a breakthrough direction for intractable infections such as TB.

### 6.6 Combination Treatment Strategies

#### 6.6.1 Chemotherapy

Chemotherapy is one of the primary treatments for gastrointestinal tumors, and the widespread application of neoadjuvant therapy in particular has provided long‐term clinical benefits for patients with cancer. However, current preclinical data from mouse models indicate that chemotherapy can promote tumor metastasis [352, 353]. This metastasis‐promoting ability may be associated with enhanced metastatic potential of cancer cells in the primary tumor and the inhibition of T‐cell function [354]. With the continuous exploration of underlying mechanisms, it has been found that the response of MAIT cells to chemotherapy is different from that of conventional T‐cell subsets. Turtle et al. [355] showed that CD8⁺CD161^hi^CD218^hi^ T cells exhibit chemoresistance both in vitro and in vivo. Drug efflux assays revealed that these cells possess high ATP‐binding cassette (ABC) transporter activity, enabling rapid efflux of ABCB1 substrate drug [355]. Multiple shared characteristics now confirm that this subset corresponds to MAIT cells [355, 356]. Similarly, Dusseaux et al. [33] and Fergusson et al. [357] verified that, compared with other CD8⁺ T‐cell subsets, MAIT cells uniquely and efficiently efflux the cytotoxic anthracycline drug daunorubicin, maintaining normal function upon daunorubicin exposure. In CRC, the FOLFOX regimen (comprising leucovorin, fluorouracil, and oxaliplatin) is currently a commonly used chemotherapy protocol. Studies have demonstrated that preoperative administration of FOLFOX or a combination FOLFOX with bevacizumab, does not reduce the frequency of MAIT cells or their ability to produce IFN‐γ in the peripheral blood, healthy liver tissue, or metastatic tumors of patients [206, 287]. Nevertheless, MAIT cells are not resistant to all chemotherapeutic agents. Novak et al. [227] observed in patients with hematological malignancies that myeloablative chemotherapy reduces the number of peripheral MAIT cells; approximately 30 days posttreatment, the median number of MAIT cells increased to 43% and remained stable in subsequent measurements at 60 and 100 days. Furthermore, 33% of patients achieved a full recovery of MAIT cell numbers (reaching 100% of pretreatment levels) within 60 days. Further analysis identified age as the only factor influencing MAIT cell recovery, with younger age correlating with earlier MAIT cell restoration. The pretreatment number of MAIT cells also exerted a prognostic impact on the early posttransplantation disease course [227]. These studies suggest that the contribution of MAIT cells to antitumor immunity may be particularly prominent in the context of combination therapy, where chemotherapy‐sensitive CTLs are eliminated.

#### 6.6.2 Immunotherapy

In recent years, ICB has achieved significant progress in cancer treatment. As revolutionary immunotherapeutic agents, ICIs enhance the body's ability to attack cancer cells by blocking key pathways in the immune system. These drugs primarily target critical immune checkpoint molecules, such as CTLA‐4, PD‐1, and PD‐L1, effectively combating cancer by activating the patient's immune response [358]. MAIT cells in the TME may express multiple ICI targets, including PD‐1, CTLA‐4, Lag‐3, and Tim‐3. These immune checkpoints form an immunosuppressive microenvironment, impairing the tumor‐killing capacity and immunomodulatory functions of MAIT cells. Sasson et al. [359] analyzed intestinal‐infiltrating and circulating lymphocytes in patients with melanoma receiving combined ipilimumab and nivolumab treatment and found that the combination therapy activated MAIT cells. Similarly, Favreau et al. [213] observed elevated PD‐1 levels on MAIT cells in the bone marrow and alveoli of patients with MM; PD‐1 blockade successfully reactivated these MAIT cells, leading to a significant reduction in in vivo tumor burden. A research team from Italy evaluated changes in circulating CD8⁺ T cells in 28 patients with metastatic melanoma receiving anti‐PD‐1 therapy using scRNA‐seq and flow cytometry. MAIT cells were found to be more abundant with a higher activation ratio in patients who responded to immunotherapy. Thus, MAIT cells can be considered a biomarker for patients who respond to anti‐PD‐1 treatment [360]. Analysis of differences in the expression patterns of tumor‐infiltrating MAIT cells before and after anti‐PD‐1 therapy in squamous cell carcinoma (SCC) and basal cell carcinoma (BCC) revealed that HLA‐DRB1, GZMH, CCL4, and CCL5 were upregulated posttreatment in both cancer types. In SCC, multiple granzyme‐encoding genes (e.g., *GZMA*, *GZMM*, and *GZMK*) showed significantly increased expression in intratumoral MAIT cells following PD‐1 blockade. In BCC, posttreatment MAIT cells exhibited higher expression levels of IFN‐γ and GNLY. Collectively, these results indicate that anti‐PD‐1 therapy promotes the expression of effector genes in MAIT cells within specific TMEs [232, 361]. Recent preclinical trials and human clinical studies have shown that the efficacy of ICIs is influenced by the patient's gut microbiota [362, 363], suggesting a close interactive relationship among ICIs, the gut microbiota, and MAIT cells. Whether the microbiota enhances ICI efficacy by increasing MAIT cell frequency or activating MAIT cells warrants further investigation.

### 6.7 Metabolic Reprogramming Strategies

In recent years, metabolic reprogramming targeting MAIT cells has emerged as a novel interventional strategy to modulate their function and differentiation. It aims to regulate cellular metabolic pathways to enhance the antipathogen capacity of MAIT cells or inhibit their detrimental effects in chronic inflammation. As a type of innate‐like T cell, MAIT cell function is highly dependent on cellular metabolic status—in particular, after activation, they undergo significant metabolic reprogramming, including upregulated glycolysis and adaptive mitochondrial metabolism, to support the production of effector molecules such as IFN‐γ and GZMB [284, 285, 364, 365]. Studies have shown that upon TCR activation, MAIT cells rapidly upregulate the glucose transporter GLUT‐1 and the amino acid transporter SLC7A5, thereby enhancing glycolytic flux. This process is tightly regulated by the master metabolic regulators MYC and mTORC1 [365]. Inhibition of glycolysis significantly impairs the production of IFN‐γ and cytotoxic molecules, highlighting the central role of glucose metabolism in MAIT cell effector functions [284, 285]. However, in metabolic diseases such as obesity and type 2 diabetes, MAIT cells exhibit metabolic dysregulation, including reduced glycolytic capacity, mitochondrial dysfunction, and ROS accumulation. These abnormalities further lead to enhanced pathological IL‐17 responses [264, 285, 364]. Notably, the accumulation of mitochondrial ROS (mROS) is directly associated with excessive IL‐17 production by MAIT cells in obesity. Scavenging ROS with antioxidants (e.g., glutathione) can significantly inhibit IL‐17 secretion and restore insulin sensitivity [364, 366], suggesting that targeting mitochondrial metabolism and oxidative stress is an effective strategy to correct MAIT cell dysfunction. In addition, lipid metabolism plays a critical role in the functional heterogeneity of MAIT cells. Recent studies have found that IL‐17‐producing MAIT cell subsets exhibit higher fatty acid uptake and mitochondrial membrane potential, implying that lipid metabolic reprogramming may drive their inflammatory phenotype [367]. Therefore, interventions using with small‐molecule inhibitors or metabolic substrates (e.g., regulating glucose utilization, fatty acid oxidation, or antioxidant pathways) may reprogram the metabolic state of MAIT cells, thereby reversing their pathogenic role in metabolic diseases and restoring protective functions. For example, supplementation with glutathione in obesity models not only reduces mROS levels but also improves MAIT cell metabolic health and alleviates IL‐17‐mediated insulin resistance [364, 366]. Similarly, modulating nutrient‐sensing pathways using mTORC1 or MYC inhibitors may limit the overactivated responses of MAIT cells [365]. Although most current evidence comes from in vitro studies and mouse models, these findings still strongly support targeting MAIT cell metabolism as a potential immunotherapeutic intervention. Future research needs to further validate its therapeutic potential in human disease models and optimize specific delivery strategies to avoid systemic immunosuppression [368].

Briefly, MAIT cell‐targeted therapeutic strategies reshape their functional states and interactions with the tissue microenvironment through multidimensional approaches, including MR1 ligand modulation, cytokine signal optimization, CAR engineering, microbiota–immune axis intervention, and metabolic reprogramming. Thereby, they exhibit the potential for precise regulation in anti‐infection and antitumor applications, as well as in immune‐related diseases. However, their dual functional properties, tissue specificity, and individual heterogeneity need to be further elucidated through spatiotemporal dynamic studies and combined intervention strategies to advance their clinical translation.

## 7 Conclusion and Outlook

MAIT cells, as unique sentinels of the immune system, have demonstrated complex and critical roles in defense against infection, inflammation regulation, and tumor immunity through their MR1‐dependent antigen recognition mechanism, tissue‐enrichment characteristics, and innate–adaptive immune bridge function. The central conclusion of this review is that MAIT cells are evolutionarily conserved rapid‐response units of the mucosal barrier that are activated by sensing microbial riboflavin metabolites, and subsequently secrete IFN‐γ, TNF‐α, IL‐17, and thereby exert cytotoxic effects, serving as the vanguard in the defense against bacterial/fungal infections. However, the function of MAIT cells has significant duality and tissue specificity—in microenvironments such as the intestines, lungs, or liver, it can secrete IL‐22 to promote tissue repair; however, it can also be converted into a proinflammatory mediator in autoimmune diseases and metabolic diseases, directly damaging tissues or amplifying pathogenic pathways such as Th17. In oncology, MAIT cells play paradoxical roles—they may exert limited antitumor effects in the early stages; however, the TME often induces high expression of inhibitory receptors, such as PD‐1 and TIM‐3, thereby driving the secretion of protumor factors, such as IL‐17 and IL‐8, and even suppressing other antitumor immune cells, ultimately turning accomplices of the tumor. The core of this functional plasticity lies in the precise combination of signals provided by the tissue microenvironment, including local cytokine networks, microbial metabolite profiles, matrix–cell interactions, and the dynamic balance of inhibitory receptor pathways.

Future research should focus on three practical directions: (1) Detailed analysis of tissue‐specific regulatory mechanisms. At present, it is urgent to clarify the key homing receptors and residency‐maintaining signals that drive MAIT cells to persistently reside in different tissues and to reveal the molecular switches underlying their functional polarization. This requires integrating spatial transcriptomics and high‐resolution imaging techniques for in situ mapping of the interaction patterns between MAIT cells and neighboring epithelial cells, stromal cells, and specific microbiota. In particular, attention should be given to clarifying how symbiotic bacterial metabolites finely regulate their activation thresholds and cytokine secretion profiles through MR1‐dependent or nondependent pathways, thereby explaining their contradictory behaviors in diseases such as IBD and tumors. (2) Acceleration of translational potential. In the development of treatment strategies, efforts should be made to design tissue‐targeted MR1 ligand drugs—optimize the stability and mucosal delivery efficiency of 5‐OP‐RU analogs and develop them as adjuvants for vaccines against multidrug‐resistant bacterial infections; at the same time, design high‐affinity MR1 antagonists to block the pathogenic activation of MAIT cells in autoimmune diseases. In the field of tumor immunotherapy, a stratified strategy must be implemented—for tumors with enriched MAIT cells and a tumor‐promoting phenotype, prioritize reversing the suppression/tumor‐promoting phenotype of MAIT cells and assess their synergistic effects with existing immunotherapies; design CAR–MAIT cells targeting tumor antigens to enhance solid tumor infiltration using their natural homing properties; for clearly tumor‐promoting subpopulations, develop clearance therapies based on specific surface markers. Concurrently advance biomarker clinical validation through multicenter cohort studies to confirm the association between disease activity and changes in the frequency of specific MAIT cell subpopulations in peripheral blood or mucosal samples, thereby advancing their use as noninvasive monitoring tools. (3) Overcoming technical bottlenecks. Existing mouse models (MR1‐KO or TCR transgenic mice) struggle to replicate the heterogeneity and functional complexity of human MAIT cells. An urgent need exists to develop an upgraded humanized model—transplanting human HSCs into immunodeficient mice carrying the human *MR1* gene and colonizing them with a human microbiome to reconstruct immune–microbiome interactions; construct a 3D coculture chip containing intestinal organoids, stromal cells, microbiota, and MAIT cells to simulate pathological microenvironments for drug screening. Additionally, optimizing in vitro expansion protocols is essential—on the basis of IL‐7/IL‐15/IL‐2 and MR1 ligand stimulation, add costimulatory signals and inhibitory receptor blockers to expand functionally intact, low‐exhaustion MAIT cells that retain tissue‐homing capacity, paving the way for adoptive therapy.

In summary, along the path from basic mechanisms to clinical translation, the tissue tropism, rapid efficacy, and microenvironment sensitivity of MAIT cells are both core advantages and challenges. Only through the integration of interdisciplinary technologies and precise regulatory strategies can we achieve “bidirectional control” of their functions—activating their mucosal defense potential during infection and blocking their pathogenic pathways in inflammation and tumors. This will ultimately pave the way for a new therapeutic paradigm based on the “immune–metabolic–microbiome” axis for the prevention and treatment of major human diseases.

## Author Contributions

**Cheng Zhu**: conceptualization, visualization, funding acquisition, and writing – original draft. **Qian Huai, Yishan Du, and Xingyu Li**: resources, software, and writing – review and editing. **Fumin Zhang, Yongkang Zhang, and Mengwei Wu**: supervision, resources, and writing – review and editing. **Ying Dai, Hanren Dai, Xiaolei Li, and Hua Wang**: conceptualization, supervision, funding acquisition, and writing – review and editing. All authors have read and approved the final manuscript.

## Ethics Statement

The study complied with the Declaration of Helsinki, and all subjects gave informed consent for inclusion in the study (The First Affiliated Hospital of Anhui Medical University, approval number: PJ2024‐03‐20). Written informed consent was obtained from all patients involved in the study.

## Conflicts of Interest

The authors declare no conflicts of interest.

## Data Availability

The data that support the findings of this study are openly available in TCGA (https://portal.gdc.cancer.gov), ICGC (https://dcc.icgc.org/), and GEO (https://www.ncbi.nlm.nih.gov/geo/).

## References

[1] D. I. Godfrey , H. F. Koay , J. McCluskey , and N. A. Gherardin , “The Biology and Functional Importance of MAIT Cells,” Nature Immunology 20, no. 9 (2019): 1110–1128. 31406380 10.1038/s41590-019-0444-8

[2] F. Legoux , M. Salou , and O. Lantz , “MAIT Cell Development and Functions: The Microbial Connection,” Immunity 53, no. 4 (2020): 710–723. 33053329 10.1016/j.immuni.2020.09.009

[3] W. Wang , C. Dai , P. Zhu , et al., “Liver Transplant‐facilitated CD161+Vα7.2+ MAIT Cell Recovery Demonstrates Clinical Benefits in Hepatic Failure Patients,” Nature Communications 16, no. 1 (2025): 4022. 10.1038/s41467-025-59308-x PMC12041255 40301342

[4] Y. Zhang , H. Liu , D. Liu , et al., “Hantaan Virus Infection Induces human Mucosal‐associated Invariant T Cell Pyroptosis Through IRE1α Pathway,” Communications Biology 8, no. 1 (2025): 538. 40169922 10.1038/s42003-025-07979-z PMC11961572

[5] A. Varelias , M. D. Bunting , K. L. Ormerod , et al., “Recipient Mucosal‐associated Invariant T Cells Control GVHD Within the Colon,” The Journal of Clinical Investigation 128, no. 5 (2018): 1919–1936. 29629900 10.1172/JCI91646 PMC5919881

[6] A. Willing , J. Jäger , S. Reinhardt , N. Kursawe , and M. A. Friese , “Production of IL‐17 by MAIT Cells Is Increased in Multiple Sclerosis and Is Associated With IL‐7 Receptor Expression,” The Journal of Immunology 200, no. 3 (2018): 974–982. 29298833 10.4049/jimmunol.1701213

[7] N. A. Gherardin , L. Loh , L. Admojo , et al., “Enumeration, Functional Responses and Cytotoxic Capacity of MAIT Cells in Newly Diagnosed and Relapsed Multiple Myeloma,” Scientific Reports 8, no. 1 (2018): 4159. 29515123 10.1038/s41598-018-22130-1 PMC5841305

[8] B. Ruf , V. V. Catania , S. Wabitsch , et al., “Activating Mucosal‐Associated Invariant T Cells Induces a Broad Antitumor Response,” Cancer Immunology Research 9, no. 9 (2021): 1024–1034. 34193462 10.1158/2326-6066.CIR-20-0925 PMC8416791

[9] M. M. Pisarska , M. R. Dunne , D. O'Shea , and A. E. Hogan , “Interleukin‐17 Producing Mucosal Associated Invariant T Cells—emerging Players in Chronic Inflammatory Diseases?,” European Journal of Immunology 50, no. 8 (2020): 1098–1108. 32617963 10.1002/eji.202048645

[10] Q. Huang , L. Duan , X. Qian , et al., “IL‐17 Promotes Angiogenic Factors IL‐6, IL‐8, and Vegf Production via Stat1 in Lung Adenocarcinoma,” Scientific Reports 6 (2016): 36551. 27819281 10.1038/srep36551 PMC5098156

[11] P. Wu , D. Wu , C. Ni , et al., “γδT17 cells Promote the Accumulation and Expansion of Myeloid‐derived Suppressor cells in human Colorectal Cancer,” Immunity 40, no. 5 (2014): 785–800. 24816404 10.1016/j.immuni.2014.03.013 PMC4716654

[12] P. Kulig , S. Burkhard , J. Mikita‐Geoffroy , et al., “IL17A‐Mediated Endothelial Breach Promotes Metastasis Formation,” Cancer Immunology Research 4, no. 1 (2016): 26–32. 26586773 10.1158/2326-6066.CIR-15-0154

[13] S. Porcelli , C. E. Yockey , M. B. Brenner , and S. P. Balk , “Analysis of T Cell Antigen Receptor (TCR) Expression by human Peripheral Blood CD4‐8‐ alpha/Beta T Cells Demonstrates Preferential Use of Several V Beta Genes and an Invariant TCR Alpha Chain,” The Journal of Experimental Medicine 178, no. 1 (1993): 1–16. 8391057 10.1084/jem.178.1.1 PMC2191070

[14] F. Tilloy , E. Treiner , S. H. Park , et al., “An Invariant T Cell Receptor Alpha Chain Defines a Novel TAP‐independent Major Histocompatibility Complex Class Ib‐restricted Alpha/Beta T Cell Subpopulation in Mammals,” The Journal of Experimental Medicine 189, no. 12 (1999): 1907–1921. 10377186 10.1084/jem.189.12.1907 PMC2192962

[15] E. Treiner , L. Duban , S. Bahram , et al., “Selection of Evolutionarily Conserved Mucosal‐associated Invariant T Cells by MR1,” Nature 422, no. 6928 (2003): 164–169. 12634786 10.1038/nature01433

[16] E. Martin , E. Treiner , L. Duban , et al., “Stepwise Development of MAIT Cells in Mouse and Human,” PLoS Biology 7, no. 3 (2009): e1000054. 19278296 10.1371/journal.pbio.1000054 PMC2653554

[17] L. Le Bourhis , E. Martin , and I. Péguillet , “Antimicrobial Activity of Mucosal‐associated Invariant T Cells,” Nature Immunology 11, no. 8 (2010): 701–708. 20581831 10.1038/ni.1890

[18] M. C. Gold , S. Cerri , S. Smyk‐Pearson , et al., “Human Mucosal Associated Invariant T Cells Detect Bacterially Infected Cells,” PLoS Biology 8, no. 6 (2010): e1000407. 20613858 10.1371/journal.pbio.1000407 PMC2893946

[19] L. Kjer‐Nielsen , O. Patel , A. J. Corbett , et al., “MR1 presents Microbial Vitamin B Metabolites to MAIT Cells,” Nature 491, no. 7426 (2012): 717–723. 23051753 10.1038/nature11605

[20] A. J. Corbett , S. B. Eckle , R. W. Birkinshaw , et al., “T‐cell Activation by Transitory Neo‐antigens Derived From Distinct Microbial Pathways,” Nature 509, no. 7500 (2014): 361–365. 24695216 10.1038/nature13160

[21] A. Rahimpour , H. F. Koay , A. Enders , et al., “Identification of Phenotypically and Functionally Heterogeneous Mouse Mucosal‐associated Invariant T Cells Using MR1 Tetramers,” The Journal of Experimental Medicine 212, no. 7 (2015): 1095–1108. 26101265 10.1084/jem.20142110 PMC4493408

[22] R. Reantragoon , A. J. Corbett , I. G. Sakala , et al., “Antigen‐loaded MR1 Tetramers Define T Cell Receptor Heterogeneity in Mucosal‐associated Invariant T Cells,” The Journal of Experimental Medicine 210, no. 11 (2013): 2305–2320. 24101382 10.1084/jem.20130958 PMC3804952

[23] Y. R. Li , J. Brown , Y. Yu , et al., “Targeting Immunosuppressive Tumor‐Associated Macrophages Using Innate T Cells for Enhanced Antitumor Reactivity,” Cancers 14, no. 11 (2022): 2749. 35681730 10.3390/cancers14112749 PMC9179365

[24] M. Dogan , E. Karhan , L. Kozhaya , et al., “Engineering Human MAIT Cells With Chimeric Antigen Receptors for Cancer Immunotherapy,” Journal of Immunology (Baltimore, Md: 1950) 209, no. 8 (2022): 1523–1531. 36165183 10.4049/jimmunol.2100856

[25] M. Dey , M. H. Kim , M. Nagamine , et al., “Biofabrication of 3D Breast Cancer Models for Dissecting the Cytotoxic Response of human T Cells Expressing Engineered MAIT Cell Receptors,” Biofabrication 14, no. 4 (2022). 10.1088/1758-5090/ac925a PMC9556424 36108605

[26] H. Wakao , K. Yoshikiyo , U. Koshimizu , et al., “Expansion of Functional Human Mucosal‐Associated Invariant T Cells via Reprogramming to Pluripotency and Redifferentiation,” Cell Stem Cell 12, no. 5 (2013): 546–558. 23523177 10.1016/j.stem.2013.03.001

[27] T. Leng , H. D. Akther , C. P. Hackstein , et al., “TCR and Inflammatory Signals Tune Human MAIT Cells to Exert Specific Tissue Repair and Effector Functions,” Cell Reports 28, no. 12 (2019): 3077–3091. e5. 31533032 10.1016/j.celrep.2019.08.050 PMC6899450

[28] A. Kurioka , L. J. Walker , P. Klenerman , and C. B. Willberg , “MAIT Cells: New Guardians of the Liver,” Clinical & Translational Immunology 5, no. 8 (2016): e98. 27588203 10.1038/cti.2016.51 PMC5007630

[29] N. E. Serriari , M. Eoche , L. Lamotte , et al., “Innate Mucosal‐associated Invariant T (MAIT) Cells Are Activated in Inflammatory Bowel Diseases,” Clinical and Experimental Immunology 176, no. 2 (2014): 266–274. 24450998 10.1111/cei.12277 PMC3992039

[30] C. Cosgrove , J. E. Ussher , A. Rauch , et al., “Early and Nonreversible Decrease of CD161++/MAIT Cells in HIV Infection,” Blood 121, no. 6 (2013): 951–961. 23255555 10.1182/blood-2012-06-436436 PMC3567342

[31] J. R. Fergusson , M. H. Hühn , L. Swadling , et al., “CD161(int)CD8+ T Cells: A Novel Population of Highly Functional, Memory CD8+ T Cells Enriched Within the Gut,” Mucosal Immunology 9, no. 2 (2016): 401–413. 26220166 10.1038/mi.2015.69 PMC4732939

[32] E. Leeansyah , A. Ganesh , M. F. Quigley , et al., “Activation, Exhaustion, and Persistent Decline of the Antimicrobial MR1‐restricted MAIT‐cell Population in Chronic HIV‐1 Infection,” Blood 121, no. 7 (2013): 1124–1135. 23243281 10.1182/blood-2012-07-445429 PMC3575756

[33] M. Dusseaux , E. Martin , N. Serriari , et al., “Human MAIT Cells Are Xenobiotic‐resistant, Tissue‐targeted, CD161hi IL‐17‐secreting T Cells,” Blood 117, no. 4 (2011): 1250–1259. 21084709 10.1182/blood-2010-08-303339

[34] X. Z. Tang , J. Jo , and A. T. Tan , “IL‐7 Licenses Activation of human Liver Intrasinusoidal Mucosal‐associated Invariant T Cells,” Journal of Immunology (Baltimore, Md: 1950) 190, no. 7 (2013): 3142–3152. 23447689 10.4049/jimmunol.1203218

[35] H. C. Jeffery , B. van Wilgenburg , A. Kurioka , et al., “Biliary Epithelium and Liver B Cells Exposed to Bacteria Activate Intrahepatic MAIT Cells Through MR1,” Journal of Hepatology 64, no. 5 (2016): 1118–1127. 26743076 10.1016/j.jhep.2015.12.017 PMC4822535

[36] T. Hinks , J. Wallington , A. Williams , R. Djukanovic , K. Staples , and T. Wilkinson , “Steroid‐induced Deficiency of Mucosal‐associated Invariant T Cells in the COPD Lung: Implications for NTHi Infection,” American Journal of Respiratory and Critical Care Medicine 194 (2016): 1208–1218. 27115408 10.1164/rccm.201601-0002OC PMC5114442

[37] A. Gibbs , E. Leeansyah , A. Introini , et al., “MAIT Cells Reside in the Female Genital Mucosa and Are Biased towards IL‐17 and IL‐22 Production in Response to Bacterial Stimulation,” Mucosal Immunology 10, no. 1 (2017): 35–45. 27049062 10.1038/mi.2016.30 PMC5053908

[38] M. Lepore , A. Kalinichenko , A. Colone , et al., “Parallel T‐cell Cloning and Deep Sequencing of human MAIT Cells Reveal Stable Oligoclonal TCRβ Repertoire,” Nature Communications 5, no. 1 (2014): 3866. 10.1038/ncomms4866 24832684

[39] M. J. Sobkowiak , H. Davanian , R. Heymann , et al., “Tissue‐resident MAIT Cell Populations in human Oral Mucosa Exhibit an Activated Profile and Produce IL‐17,” European Journal of Immunology 49, no. 1 (2019): 133–143. 30372518 10.1002/eji.201847759 PMC6519349

[40] J. M. Eberhard , P. Hartjen , S. Kummer , et al., “CD161+ MAIT Cells Are Severely Reduced in Peripheral Blood and Lymph Nodes of HIV‐Infected Individuals Independently of Disease Progression,” PLoS ONE 9, no. 11 (2014): e111323. 25369333 10.1371/journal.pone.0111323 PMC4219715

[41] Y. S. Kwon , H.‐M. Jin , Y.‐N. Cho , et al., “Mucosal‐Associated Invariant T Cell Deficiency in Chronic Obstructive Pulmonary Disease,” COPD 13, no. 2 (2016): 196–202. 26552490 10.3109/15412555.2015.1069806

[42] N. A. Gherardin , M. N. Souter , H. F. Koay , et al., “Human Blood MAIT Cell Subsets Defined Using MR1 Tetramers,” Immunology and Cell Biology 96, no. 5 (2018): 507–525. 29437263 10.1111/imcb.12021 PMC6446826

[43] J. Novak , J. Dobrovolny , L. Novakova , and T. Kozak , “The Decrease in Number and Change in Phenotype of Mucosal‐associated Invariant T Cells in the Elderly and Differences in Men and Women of Reproductive Age,” Scandinavian Journal of Immunology 80, no. 4 (2014): 271–275. 24846411 10.1111/sji.12193

[44] I. Kawachi , J. Maldonado , C. Strader , and S. Gilfillan , “MR1‐Restricted Vα19i Mucosal‐Associated Invariant T Cells Are Innate T Cells in the Gut Lamina Propria That Provide a Rapid and Diverse Cytokine Response1,” The Journal of Immunology 176, no. 3 (2006): 1618–1627. 16424191 10.4049/jimmunol.176.3.1618

[45] Y. Cui , K. Franciszkiewicz , Y. K. Mburu , et al., “Mucosal‐associated Invariant T Cell‐rich Congenic Mouse Strain Allows Functional Evaluation,” The Journal of Clinical Investigation 125, no. 11 (2015): 4171–4185. 26524590 10.1172/JCI82424 PMC4639991

[46] S. Jahreis , S. Böttcher , S. Hartung , et al., “Human MAIT Cells Are Rapidly Activated by Aspergillus spp. in an APC‐dependent Manner,” European Journal of Immunology 48, no. 10 (2018): 1698–1706. 30059139 10.1002/eji.201747312

[47] T. Sato , H. Thorlacius , B. Johnston , et al., “Role for CXCR6 in Recruitment of Activated CD8+ Lymphocytes to Inflamed Liver1,” The Journal of Immunology 174, no. 1 (2005): 277–283. 15611250 10.4049/jimmunol.174.1.277

[48] E. Billerbeck , Y. H. Kang , L. Walker , et al., “Analysis of CD161 Expression on human CD8+ T Cells Defines a Distinct Functional Subset With Tissue‐homing Properties,” Proceedings of the National Academy of Sciences of the United States of America 107, no. 7 (2010): 3006–3011. 20133607 10.1073/pnas.0914839107 PMC2840308

[49] J. Jo , A. T. Tan , J. E. Ussher , et al., “Toll‐Like Receptor 8 Agonist and Bacteria Trigger Potent Activation of Innate Immune Cells in human Liver,” PLoS Pathogens 10, no. 6 (2014): e1004210. 24967632 10.1371/journal.ppat.1004210 PMC4072808

[50] S. P. Singh , H. H. Zhang , H. Tsang , et al., “PLZF Regulates CCR6 and Is Critical for the Acquisition and Maintenance of the Th17 Phenotype in human Cells,” Journal of Immunology (Baltimore, Md: 1950) 194, no. 9 (2015): 4350–4361. 25833398 10.4049/jimmunol.1401093 PMC4412463

[51] L. Le Bourhis , M. Dusseaux , and A. Bohineust , “MAIT Cells Detect and Efficiently Lyse Bacterially‐infected Epithelial Cells,” PLoS Pathogens 9, no. 10 (2013): e1003681. 24130485 10.1371/journal.ppat.1003681 PMC3795036

[52] N. M. Provine , B. Binder , M. E. B. FitzPatrick , et al., “Unique and Common Features of Innate‐Like Human Vδ2+ γδT Cells and Mucosal‐Associated Invariant T Cells,” Original Research 9 (2018): 756. 10.3389/fimmu.2018.00756 PMC5924964 29740432

[53] M. Gutierrez‐Arcelus , N. Teslovich , A. R. Mola , et al., “Lymphocyte Innateness Defined by Transcriptional States Reflects a Balance Between Proliferation and Effector Functions,” Nature Communications 10, no. 1 (2019): 687. 10.1038/s41467-019-08604-4 PMC6368609 30737409

[54] D. Kovalovsky , O. U. Uche , S. Eladad , et al., “The BTB–zinc Finger Transcriptional Regulator PLZF Controls the Development of Invariant Natural Killer T Cell Effector Functions,” Nature Immunology 9, no. 9 (2008): 1055–1064. 18660811 10.1038/ni.1641 PMC2662733

[55] I. I. Ivanov , B. S. McKenzie , L. Zhou , et al., “The Orphan Nuclear Receptor RORgammat Directs the Differentiation Program of Proinflammatory IL‐17+ T Helper Cells,” Cell 126, no. 6 (2006): 1121–1133. 16990136 10.1016/j.cell.2006.07.035

[56] R. P. Wilson , M. L. Ives , G. Rao , et al., “STAT3 is a Critical Cell‐intrinsic Regulator of human Unconventional T Cell Numbers and Function,” The Journal of Experimental Medicine 212, no. 6 (2015): 855–864. 25941256 10.1084/jem.20141992 PMC4451129

[57] E. L. Pearce , A. C. Mullen , G. A. Martins , et al., “Control of Effector CD8+ T Cell Function by the Transcription Factor Eomesodermin,” Science (New York, NY) 302, no. 5647 (2003): 1041–1043. 10.1126/science.1090148 14605368

[58] S. J. Szabo , S. T. Kim , G. L. Costa , X. Zhang , C. G. Fathman , and L. H. Glimcher , “A Novel Transcription Factor, T‐bet, Directs Th1 Lineage Commitment,” Cell 100, no. 6 (2000): 655–669. 10761931 10.1016/s0092-8674(00)80702-3

[59] A. Kallies , A. Xin , G. T. Belz , and S. L. Nutt , “Blimp‐1 Transcription Factor Is Required for the Differentiation of Effector CD8(+) T Cells and Memory Responses,” Immunity 31, no. 2 (2009): 283–295. 19664942 10.1016/j.immuni.2009.06.021

[60] H. F. Koay , N. A. Gherardin , A. Enders , et al., “A Three‐stage Intrathymic Development Pathway for the Mucosal‐associated Invariant T Cell Lineage,” Nature Immunology 17, no. 11 (2016): 1300–1311. 27668799 10.1038/ni.3565

[61] S. J. Winter , H. Kunze‐Schumacher , E. Imelmann , Z. Grewers , T. Osthues , and A. Krueger , “MicroRNA miR‐181a/b‐1 Controls MAIT Cell Development,” Immunology and Cell Biology 97, no. 2 (2019): 190–202. 30291723 10.1111/imcb.12211

[62] F. Legoux , J. Gilet , E. Procopio , K. Echasserieau , K. Bernardeau , and O. Lantz , “Molecular Mechanisms of Lineage Decisions in Metabolite‐specific T Cells,” Nature Immunology 20, no. 9 (2019): 1244–1255. 31431722 10.1038/s41590-019-0465-3

[63] E. Martin , E. Treiner , L. Duban , et al., “Stepwise Development of MAIT Cells in Mouse and Human,” PLoS Biology 7 (2009): e54. 19278296 10.1371/journal.pbio.1000054 PMC2653554

[64] H. Wang , M. N. T. Souter , M. de Lima Moreira , et al., “MAIT Cell Plasticity Enables Functional Adaptation That Drives Antibacterial Immune Protection,” Science Immunology 9, no. 102 (2024): eadp9841. 39642244 10.1126/sciimmunol.adp9841

[65] H.‐F. Koay , N. A. Gherardin , A. Enders , et al., “A Three‐stage Intrathymic Development Pathway for the Mucosal‐associated Invariant T Cell Lineage,” Nature Immunology 17 (2016): 1300–1311. 27668799 10.1038/ni.3565

[66] E. W. Meermeier , M. J. Harriff , E. Karamooz , and D. M. Lewinsohn , “MAIT Cells and Microbial Immunity,” Immunology and Cell Biology 96, no. 6 (2018): 607–617. 29451704 10.1111/imcb.12022 PMC6045460

[67] A. Kurioka , J. E. Ussher , C. Cosgrove , et al., “MAIT Cells Are Licensed Through Granzyme Exchange to Kill Bacterially Sensitized Targets,” Mucosal Immunology 8, no. 2 (2015): 429–440. 25269706 10.1038/mi.2014.81 PMC4288950

[68] Z. Chen , H. Wang , and C. D'Souza , “Mucosal‐associated Invariant T‐cell Activation and Accumulation After in Vivo Infection Depends on Microbial Riboflavin Synthesis and co‐stimulatory Signals,” Mucosal Immunology 10, no. 1 (2017): 58–68. 27143301 10.1038/mi.2016.39

[69] W.‐J. Chua , S. M. Truscott , C. S. Eickhoff , et al., “Polyclonal Mucosa‐Associated Invariant T Cells Have Unique Innate Functions in Bacterial Infection,” Infection and Immunity 80 (2012): 3256–3267. 22778103 10.1128/IAI.00279-12 PMC3418730

[70] A. I. Meierovics , W.‐J. C. Yankelevich , and S. C. Cowley , “MAIT Cells Are Critical for Optimal Mucosal Immune Responses During in Vivo Pulmonary Bacterial Infection,” PNAS 110 (2013): E3119–E3128. 23898209 10.1073/pnas.1302799110 PMC3746930

[71] O. Sortino , E. Richards , J. Dias , E. Leeansyah , J. K. Sandberg , and I. Sereti , “IL‐7 Treatment Supports CD8+ Mucosa‐associated Invariant T‐cell Restoration in HIV‐1‐infected Patients on Antiretroviral Therapy,” AIDS (London, England) 32, no. 6 (2018): 825–828. 29543654 10.1097/QAD.0000000000001760 PMC6056340

[72] B. van Wilgenburg , I. Scherwitzl , and E. C. Hutchinson , et al., “MAIT Cells Are Activated During human Viral Infections,” Nature Communications 7 (2016): 11653. 10.1038/ncomms11653 PMC4931007 27337592

[73] B. van Wilgenburg , L. Loh , Z. Chen , et al., “MAIT Cells Contribute to Protection Against Lethal Influenza Infection in Vivo,” Nature Communications 9, no. 1 (2018): 4706. 10.1038/s41467-018-07207-9 PMC6226485 30413689

[74] Y. K. Yong , H. Y. Tan , A. Saeidi , et al., “Decrease of CD69 Levels on TCR Vα7.2+CD4+ Innate‐Like Lymphocytes Is Associated With Impaired Cytotoxic Functions in Chronic hepatitis B Virus‐infected Patients,” Innate Immun 23, no. 5 (2017): 459–467. 28606013 10.1177/1753425917714854

[75] E. Jesteadt , I. Zhang , H. Yu , A. Meierovics , W. J. Chua Yankelevich , and S. Cowley , “Interleukin‐18 Is Critical for Mucosa‐Associated Invariant T Cell Gamma Interferon Responses to Francisella Species in Vitro but Not in Vivo,” Infection and Immunity 86, no. 5 (2018): e00117–e00118. 29507084 10.1128/IAI.00117-18 PMC5913842

[76] J. E. Ussher , M. Bilton , E. Attwod , et al., “CD161++ CD8+ T Cells, Including the MAIT Cell Subset, Are Specifically Activated by IL‐12+IL‐18 in a TCR‐independent Manner,” European Journal of Immunology 44, no. 1 (2014): 195–203. 24019201 10.1002/eji.201343509 PMC3947164

[77] M. Barathan , R. Mohamed , J. Vadivelu , et al., “Peripheral Loss of CD8(+) CD161(++) TCRVα7·2(+) Mucosal‐associated Invariant T Cells in Chronic hepatitis C Virus‐infected Patients,” European Journal of Clinical Investigation 46, no. 2 (2016): 170–180. 26681320 10.1111/eci.12581

[78] L. L. Boeijen , N. R. Montanari , R. A. de Groen , et al., “Mucosal‐Associated Invariant T Cells Are More Activated in Chronic Hepatitis B, but Not Depleted in Blood: Reversal by Antiviral Therapy,” The Journal of Infectious Diseases 216, no. 8 (2017): 969–976. 28968772 10.1093/infdis/jix425

[79] F. J. Bolte , A. C. O'Keefe , and L. M. Webb , “Intra‐Hepatic Depletion of Mucosal‐Associated Invariant T Cells in Hepatitis C Virus‐Induced Liver Inflammation,” Gastroenterology 153, no. 5 (2017): 1392–1403. e2. 28780074 10.1053/j.gastro.2017.07.043 PMC5669813

[80] C. S. Fernandez , T. Amarasena , A. D. Kelleher , et al., “MAIT Cells Are Depleted Early but Retain Functional Cytokine Expression in HIV Infection,” Immunology and Cell Biology 93, no. 2 (2015): 177–188. 25348935 10.1038/icb.2014.91

[81] J. Hengst , B. Strunz , K. Deterding , et al., “Nonreversible MAIT Cell‐dysfunction in Chronic hepatitis C Virus Infection Despite Successful Interferon‐free Therapy,” European Journal of Immunology 46, no. 9 (2016): 2204–2210. 27296288 10.1002/eji.201646447

[82] F. Legoux , D. Bellet , C. Daviaud , et al., “Microbial Metabolites Control the Thymic Development of Mucosal‐associated Invariant T Cells,” Science (New York, NY) 366, no. 6464 (2019): 494–499. 10.1126/science.aaw2719 31467190

[83] A. Kurioka , B. van Wilgenburg , R. R. Javan , et al., “Diverse Streptococcus pneumoniae Strains Drive a Mucosal‐Associated Invariant T‐Cell Response through Major Histocompatibility Complex Class I–Related Molecule–Dependent and Cytokine‐Driven Pathways,” Journal of Infectious Diseases 217 (2017): 988–999. 10.1093/infdis/jix647 PMC5854017 29267892

[84] J. C. Wallington , A. P. Williams , K. J. Staples , and T. M. A. Wilkinson , “IL‐12 and IL‐7 Synergize to Control Mucosal‐associated Invariant T‐cell Cytotoxic Responses to Bacterial Infection,” The Journal of Allergy and Clinical Immunology 141, no. 6 (2018): 2182–2195. 28870466 10.1016/j.jaci.2017.08.009

[85] E. Leeansyah , J. Svärd , J. Dias , et al., “Arming of MAIT Cell Cytolytic Antimicrobial Activity Is Induced by IL‐7 and Defective in HIV‐1 Infection,” PLoS Pathogens 11, no. 8 (2015): e1005072. 26295709 10.1371/journal.ppat.1005072 PMC4546682

[86] C. J. Turtle , J. Delrow , R. C. Joslyn , et al., “Innate Signals Overcome Acquired TCR Signaling Pathway Regulation and Govern the Fate of human CD161(hi) CD8α⁺ Semi‐invariant T Cells,” Blood 118, no. 10 (2011): 2752–2762. 21791427 10.1182/blood-2011-02-334698 PMC3172793

[87] Z. Bánki , L. Krabbendam , D. Klaver , et al., “Antibody Opsonization Enhances MAIT Cell Responsiveness to Bacteria via a TNF‐dependent Mechanism,” Immunology and Cell Biology 97, no. 6 (2019): 538–551. 30695101 10.1111/imcb.12239 PMC6767153

[88] A. I. Meierovics and S. C. Cowley , “MAIT Cells Promote Inflammatory Monocyte Differentiation Into Dendritic Cells During Pulmonary Intracellular Infection,” The Journal of Experimental Medicine 213, no. 12 (2016): 2793–2809. 27799620 10.1084/jem.20160637 PMC5110023

[89] R. Salerno‐Goncalves , D. Luo , S. Fresnay , et al., “Challenge of Humans With Wild‐type Salmonella Enterica Serovar Typhi Elicits Changes in the Activation and Homing Characteristics of Mucosal‐Associated Invariant T Cells,” Frontiers in Immunology 8 (2017): 398. 28428786 10.3389/fimmu.2017.00398 PMC5382150

[90] S. B. G. Eckle , R. W. Birkinshaw , L. Kostenko , et al., “A Molecular Basis Underpinning the T Cell Receptor Heterogeneity of Mucosal‐associated Invariant T Cells,” Journal of Experimental Medicine 211, no. 8 (2014): 1585–1600. 25049336 10.1084/jem.20140484 PMC4113946

[91] J. Y. W. Mak , W. Xu , R. C. Reid , et al., “Stabilizing Short‐lived Schiff Base Derivatives of 5‐aminouracils That Activate Mucosal‐associated Invariant T Cells,” Nature Communications 8, no. 1 (2017): 14599. 10.1038/ncomms14599 PMC5344979 28272391

[92] C. Soudais , F. Samassa , M. Sarkis , et al., “In Vitro and In Vivo Analysis of the Gram‐Negative Bacteria‐Derived Riboflavin Precursor Derivatives Activating Mouse MAIT Cells,” Journal of Immunology (Baltimore, Md: 1950) 194, no. 10 (2015): 4641–4649. 25870247 10.4049/jimmunol.1403224

[93] S. B. Eckle , A. J. Corbett , A. N. Keller , et al., “Recognition of Vitamin B Precursors and Byproducts by Mucosal Associated Invariant T Cells,” The Journal of Biological Chemistry 290, no. 51 (2015): 30204–302011. 26468291 10.1074/jbc.R115.685990 PMC4683245

[94] J. E. Ussher , B. van Wilgenburg , R. F. Hannaway , et al., “TLR Signaling in human Antigen‐presenting Cells Regulates MR1‐dependent Activation of MAIT Cells,” European Journal of Immunology 46, no. 7 (2016): 1600–1614. 27105778 10.1002/eji.201545969 PMC5297987

[95] X. Xiao and J. Cai , “Mucosal‐Associated Invariant T Cells: New Insights Into Antigen Recognition and Activation,” Frontiers in Immunology 8 (2017): 1540. 29176983 10.3389/fimmu.2017.01540 PMC5686390

[96] K. Franciszkiewicz , M. Salou , F. Legoux , et al., “MHC Class I‐related Molecule, MR1, and Mucosal‐associated Invariant T Cells,” Immunological Reviews 272, no. 1 (2016): 120–138. 27319347 10.1111/imr.12423

[97] L. Loh , Z. Wang , S. Sant , et al., “Human Mucosal‐associated Invariant T Cells Contribute to Antiviral Influenza Immunity via IL‐18‐dependent Activation,” Proceedings of the National Academy of Sciences of the United States of America 113, no. 36 (2016): 10133–10138. 27543331 10.1073/pnas.1610750113 PMC5018778

[98] E. J. Won , J. K. Ju , Y. N. Cho , et al., “Clinical Relevance of Circulating Mucosal‐associated Invariant T Cell Levels and Their Anti‐cancer Activity in Patients With Mucosal‐associated Cancer,” Oncotarget 7, no. 46 (2016): 76274–76290. 27517754 10.18632/oncotarget.11187 PMC5342813

[99] J. J. Wang , C. Macardle , H. Weedon , D. Beroukas , and T. Banovic , “Mucosal‐associated Invariant T Cells Are Reduced and Functionally Immature in the Peripheral Blood of Primary Sjögren's syndrome Patients,” European Journal of Immunology 46, no. 10 (2016): 2444–2453. 27461134 10.1002/eji.201646300

[100] A. Chiba , N. Tamura , K. Yoshikiyo , et al., “Activation Status of Mucosal‐associated Invariant T Cells Reflects Disease Activity and Pathology of Systemic Lupus Erythematosus,” Arthritis Research & Therapy 19, no. 1 (2017): 58. 28288675 10.1186/s13075-017-1257-5 PMC5348792

[101] C. K. Slichter , A. McDavid , H. W. Miller , et al., “Distinct Activation Thresholds of human Conventional and Innate‐Like Memory T Cells,” JCI Insight 1, no. 8 (2016): e86292. 27331143 10.1172/jci.insight.86292 PMC4912124

[102] S. H. C. Havenith , S. L. Yong , S. M. Henson , et al., “Analysis of Stem‐cell‐Like Properties of human CD161++IL‐18Rα+ Memory CD8+ T Cells,” International Immunology 24, no. 10 (2012): 625–636. 22836020 10.1093/intimm/dxs069

[103] R. Martínez‐Barricarte , J. G. Markle , C. S. Ma , et al., “Human IFN‐γ Immunity to Mycobacteria Is Governed by both IL‐12 and IL‐23,” Science Immunology 3, no. 30 (2018): eaau6759. 30578351 10.1126/sciimmunol.aau6759 PMC6380365

[104] M.‐S. Rha , J. W. Han , J. H. Kim , et al., “Human Liver CD8+ MAIT Cells Exert TCR/MR1‐independent Innate‐Like Cytotoxicity in Response to IL‐15,” Journal of Hepatology 73, no. 3 (2020): 640–650. 32247824 10.1016/j.jhep.2020.03.033

[105] S. Jin , J. Chin , S. Seeber , et al., “TL1A/TNFSF15 directly Induces Proinflammatory Cytokines, Including TNFα, From CD3+CD161+ T Cells to Exacerbate Gut Inflammation,” Mucosal Immunology 6, no. 5 (2013): 886–899. 23250276 10.1038/mi.2012.124

[106] A. Sattler , L. G. Thiel , A. H. Ruhm , et al., “The TL1A‐DR3 Axis Selectively Drives Effector Functions in Human MAIT Cells,” The Journal of Immunology 203, no. 11 (2019): 2970–2978. 31628153 10.4049/jimmunol.1900465

[107] A. Chiba , R. Tajima , C. Tomi , Y. Miyazaki , T. Yamamura , and S. Miyake , “Mucosal‐associated Invariant T Cells Promote Inflammation and Exacerbate Disease in Murine Models of Arthritis,” Arthritis and Rheumatism 64, no. 1 (2012): 153–161. 21904999 10.1002/art.33314

[108] C. R. Shaler , J. Choi , P. T. Rudak , et al., “MAIT Cells Launch a Rapid, Robust and Distinct Hyperinflammatory Response to Bacterial Superantigens and Quickly Acquire an Anergic Phenotype That Impedes Their Cognate Antimicrobial Function: Defining a Novel Mechanism of Superantigen‐induced Immunopathology and Immunosuppression,” PLoS Biology 15, no. 6 (2017): e2001930. 28632753 10.1371/journal.pbio.2001930 PMC5478099

[109] J. K. Sandberg , A. Norrby‐Teglund , and E. Leeansyah , “Bacterial Deception of MAIT Cells in a Cloud of Superantigen and Cytokines,” PLoS Biology 15, no. 7 (2017): e2003167. 28742082 10.1371/journal.pbio.2003167 PMC5542701

[110] J. White , A. Herman , A. M. Pullen , R. Kubo , J. W. Kappler , and P. Marrack , “The V Beta‐specific Superantigen Staphylococcal Enterotoxin B: Stimulation of Mature T Cells and Clonal Deletion in Neonatal Mice,” Cell 56, no. 1 (1989): 27–35. 2521300 10.1016/0092-8674(89)90980-x

[111] M. Salio , O. Gasser , C. Gonzalez‐Lopez , et al., “Activation of Human Mucosal‐Associated Invariant T Cells Induces CD40L‐Dependent Maturation of Monocyte‐Derived and Primary Dendritic Cells,” Journal of Immunology (Baltimore, Md: 1950) 199, no. 8 (2017): 2631–2638. 28877992 10.4049/jimmunol.1700615 PMC5632842

[112] Y. R. Li , K. Zhou , Y. Zhu , T. Halladay , and L. Yang , “Breaking the Mold: Unconventional T Cells in Cancer Therapy,” Cancer Cell 43, no. 3 (2025): 317–322. 39672171 10.1016/j.ccell.2024.11.010

[113] Y.‐R. Li , K. Zhou , M. Wilson , et al., “Mucosal‐associated Invariant T Cells for Cancer Immunotherapy,” Molecular Therapy 31, no. 3 (2023): 631–646. 10.1016/j.ymthe.2022.11.019 PMC10014234 36463401

[114] P. Pinco and F. Facciotti , “Unconventional T Cells' Role in Cancer: Unlocking Their Hidden Potential to Guide Tumor Immunity and Therapy,” Cells 14, no. 10 (2025): 720. 40422223 10.3390/cells14100720 PMC12110390

[115] A. Heczey , X. Xu , A. N. Courtney , et al., “Anti‐GD2 CAR‐NKT Cells in Relapsed or Refractory Neuroblastoma: Updated Phase 1 Trial Interim Results,” Nature Medicine 29, no. 6 (2023): 1379–1388. 10.1038/s41591-023-02363-y 37188782

[116] Y. R. Li , Y. Zhou , J. Yu , et al., “Generation of Allogeneic CAR‐NKT Cells From Hematopoietic Stem and Progenitor Cells Using a Clinically Guided Culture Method,” Nature Biotechnology 43, no. 3 (2025): 329–344. 10.1038/s41587-024-02226-y PMC11919731 38744947

[117] M. Alnaggar , Y. Xu , J. Li , et al., “Allogenic Vγ9Vδ2 T Cell as New Potential Immunotherapy Drug for Solid Tumor: A Case Study for Cholangiocarcinoma,” Journal for Immunotherapy of Cancer 7, no. 1 (2019): 36. 30736852 10.1186/s40425-019-0501-8 PMC6368763

[118] A. J. Nicol , H. Tokuyama , S. R. Mattarollo , et al., “Clinical Evaluation of Autologous Gamma Delta T Cell‐based Immunotherapy for Metastatic Solid Tumours,” British Journal of Cancer 105, no. 6 (2011): 778–786. 21847128 10.1038/bjc.2011.293 PMC3171009

[119] L. Jiang , F. You , H. Wu , et al., “B7‐H3‐Targeted CAR‐Vδ1T Cells Exhibit Potent Broad‐Spectrum Activity Against Solid Tumors,” Cancer Research 84, no. 23 (2024): 4066–4080. 39240694 10.1158/0008-5472.CAN-24-0195 PMC11609632

[120] X. Zhai , F. You , S. Xiang , et al., “MUC1‐Tn‐targeting Chimeric Antigen Receptor‐modified Vγ9Vδ2 T Cells With Enhanced Antigen‐specific Anti‐tumor Activity,” American Journal of Cancer Research 11, no. 1 (2021): 79–91. 33520361 PMC7840711

[121] L. Le Bourhis , E. Martin , and I. Péguillet , “Antimicrobial Activity of Mucosal‐associated Invariant T Cells,” Nature Immunology 11, no. 8 (2010): 701–708. 20581831 10.1038/ni.1890

[122] T. S. C. Hinks and X.‐W. Zhang , “MAIT Cell Activation and Functions,” Frontiers in Immunology 11 (2020): 1014. 32536923 10.3389/fimmu.2020.01014 PMC7267072

[123] W.‐J. Chua , S. M. Truscott , C. S. Eickhoff , et al., “Polyclonal Mucosa‐Associated Invariant T Cells Have Unique Innate Functions in Bacterial Infection,” Infection and Immunity 80, no. 9 (2012): 3256–3267. 22778103 10.1128/IAI.00279-12 PMC3418730

[124] A. Meierovics , W.‐J. C. Yankelevich , and S. C. Cowley , “MAIT Cells Are Critical for Optimal Mucosal Immune Responses During in Vivo Pulmonary Bacterial Infection,” Proceedings of the National Academy of Sciences of the United States of America 110, no. 33 (2013): E3119–E3128. 23898209 10.1073/pnas.1302799110 PMC3746930

[125] J. Jiang , B. Yang , H. An , et al., “Mucosal‐associated Invariant T Cells From Patients With Tuberculosis Exhibit Impaired Immune Response,” Journal of Infection 72, no. 3 (2016): 338–352. 26724769 10.1016/j.jinf.2015.11.010

[126] E. Jesteadt , I. Zhang , H. Yu , et al., “Interleukin‐18 Is Critical for Mucosa‐Associated Invariant T Cell Gamma Interferon Responses to Francisella SpeciesIn Vitrobut NotIn Vivo,” Infection and Immunity 86, no. 5 (2018): e00117–e00118. 29507084 10.1128/IAI.00117-18 PMC5913842

[127] P. Marrack , C. Boulouis , W. R. Sia , et al., “Human MAIT Cell Cytolytic Effector Proteins Synergize to Overcome Carbapenem Resistance in Escherichia coli,” PLoS Biology 18, no. 6 (2020): e3000644. 32511236 10.1371/journal.pbio.3000644 PMC7302869

[128] M. Dusseaux , E. Martin , N. Serriari , et al., “Human MAIT Cells Are Xenobiotic‐resistant, Tissue‐targeted, CD161hi IL‐17–secreting T Cells,” Blood 117, no. 4 (2011): 1250–1259. 21084709 10.1182/blood-2010-08-303339

[129] F. R. DeLeo , L. Le Bourhis , and M. Dusseaux , “MAIT Cells Detect and Efficiently Lyse Bacterially‐Infected Epithelial Cells,” PLoS Pathogens 9, no. 10 (2013): e1003681. 24130485 10.1371/journal.ppat.1003681 PMC3795036

[130] J. Jiang , Z. Cao , L. Xiao , et al., “Tim‐3 Expression Is Induced by Mycobacterial Antigens and Identifies Tissue‐resident Subsets of MAIT Cells From Patients With Tuberculosis,” Microbes and Infection 25, no. 1‐2 (2023): 105021. 35811063 10.1016/j.micinf.2022.105021

[131] J. K. Sandberg , D. J. Smith , G. R. Hill , S. C. Bell , and D. W. Reid , “Reduced Mucosal Associated Invariant T‐Cells Are Associated With Increased Disease Severity and Pseudomonas aeruginosa Infection in Cystic Fibrosis,” PLoS ONE 9, no. 10 (2014): e109891. 25296025 10.1371/journal.pone.0109891 PMC4190362

[132] H. Wang , C. D'Souza , and X. Y. Lim , “MAIT Cells Protect Against Pulmonary Legionella Longbeachae Infection,” Nature Communications 9, no. 1 (2018): 3350. 10.1038/s41467-018-05202-8 PMC6105587 30135490

[133] R. Salerno‐Gonçalves , S. Fresnay , L. Magder , et al., “Mucosal‐Associated Invariant T Cells Exhibit Distinct Functional Signatures Associated With Protection Against Typhoid Fever,” Cellular Immunology 378 (2022): 104572. 35772315 10.1016/j.cellimm.2022.104572 PMC9377420

[134] J. M. Vinetz , D. T. Leung , T. R. Bhuiyan , et al., “Circulating Mucosal Associated Invariant T Cells Are Activated in Vibrio Cholerae O1 Infection and Associated With Lipopolysaccharide Antibody Responses,” PLoS Neglected Tropical Diseases 8, no. 8 (2014): e3076. 25144724 10.1371/journal.pntd.0003076 PMC4140671

[135] J. J. Douglas , J. L. Y. Tsang , and K. R. Walley , “Sepsis and the Innate‐Like Response,” Intensive Care Medicine 40, no. 2 (2013): 249–251. 24322274 10.1007/s00134-013-3141-3

[136] H. M. O'Hagan , W. Wang , and S. Sen , “Oxidative Damage Targets Complexes Containing DNA Methyltransferases, SIRT1, and Polycomb Members to Promoter CpG Islands,” Cancer Cell 20, no. 5 (2011): 606–619. 22094255 10.1016/j.ccr.2011.09.012 PMC3220885

[137] C. E. Niehaus , B. Strunz , M. Cornillet , et al., “MAIT Cells Are Enriched and Highly Functional in Ascites of Patients with Decompensated Liver Cirrhosis,” Hepatology 72, no. 4 (2020): 1378–1393. 32012321 10.1002/hep.31153

[138] J. Jiang , X. Chen , H. An , B. Yang , F. Zhang , and X. Cheng , “Enhanced Immune Response of MAIT Cells in Tuberculous Pleural Effusions Depends on Cytokine Signaling,” Scientific Reports 6, no. 1 (2016): 32320. 27586092 10.1038/srep32320 PMC5009363

[139] L. Ouyang , M. Wu , Z. Shen , et al., “Activation and Functional Alteration of Mucosal‐Associated Invariant T Cells in Adult Patients with Community‐Acquired Pneumonia,” Frontiers in Immunology 12 (2021): 788406. 34992604 10.3389/fimmu.2021.788406 PMC8724213

[140] R. F. Hannaway , X. Wang , M. Schneider , et al., “Mucosal‐associated Invariant T Cells and Vδ2+ Γδ T Cells in Community Acquired Pneumonia: Association of Abundance in Sputum With Clinical Severity and Outcome,” Clinical and Experimental Immunology 199, no. 2 (2020): 201–215. 31587268 10.1111/cei.13377 PMC6954682

[141] P. Georgel , M. Radosavljevic , C. Macquin , and S. Bahram , “The Non‐conventional MHC Class I MR1 Molecule Controls Infection by Klebsiella pneumoniae in Mice,” Molecular Immunology 48, no. 5 (2011): 769–775. 21190736 10.1016/j.molimm.2010.12.002

[142] J. S. Booth , R. Salerno‐Goncalves , T. G. Blanchard , et al., “Mucosal‐Associated Invariant T Cells in the Human Gastric Mucosa and Blood: Role in Helicobacter pylori Infection,” Frontiers in Immunology 6 (2015): 466. 26441971 10.3389/fimmu.2015.00466 PMC4585133

[143] C. D'Souza , T. Pediongco , and H. Wang , “Mucosal‐Associated Invariant T Cells Augment Immunopathology and Gastritis in Chronic Helicobacter pylori Infection,” The Journal of Immunology 200, no. 5 (2018): 1901–1916. 29378910 10.4049/jimmunol.1701512

[144] A. Bhandoola , C. R. Shaler , J. Choi , et al., “MAIT Cells Launch a Rapid, Robust and Distinct Hyperinflammatory Response to Bacterial Superantigens and Quickly Acquire an Anergic Phenotype That Impedes Their Cognate Antimicrobial Function: Defining a Novel Mechanism of Superantigen‐induced Immunopathology and Immunosuppression,” PLoS Biology 15, no. 6 (2017): e2001930. 28632753 10.1371/journal.pbio.2001930 PMC5478099

[145] C. Cosgrove , J. E. Ussher , A. Rauch , et al., “Early and Nonreversible Decrease of CD161++ /MAIT Cells in HIV Infection,” Blood 121, no. 6 (2013): 951–961. 23255555 10.1182/blood-2012-06-436436 PMC3567342

[146] A. Saeidi , V. L. Tien Tien , R. Al‐Batran , et al., “Attrition of TCR Vα7.2+ CD161++ MAIT Cells in HIV‐tuberculosis co‐infection Is Associated With Elevated Levels of PD‐1 Expression,” PLoS ONE 10, no. 4 (2015): e0124659. 25894562 10.1371/journal.pone.0124659 PMC4403924

[147] C. Boulouis , E. Leeansyah , S. Mairpady Shambat , A. Norrby‐Teglund , and J. K. Sandberg , “Mucosa‐Associated Invariant T Cell Hypersensitivity to Staphylococcus aureus Leukocidin ED and Its Modulation by Activation,” Journal of Immunology (Baltimore, Md: 1950) 208, no. 5 (2022): 1170–1179. 35140134 10.4049/jimmunol.2100912 PMC9012079

[148] V. H. Wu , J. M. L. Nordin , S. Nguyen , et al., “Profound Phenotypic and Epigenetic Heterogeneity of the HIV‐1‐infected CD4(+) T Cell Reservoir,” Nature Immunology 24, no. 2 (2023): 359–370. 36536105 10.1038/s41590-022-01371-3 PMC9892009

[149] S. Khuzwayo , M. Mthembu , E. W. Meermeier , et al., “MR1‐Restricted MAIT Cells from the Human Lung Mucosal Surface Have Distinct Phenotypic, Functional, and Transcriptomic Features That Are Preserved in HIV Infection,” Frontiers in Immunology 12 (2021): 631410. 33897687 10.3389/fimmu.2021.631410 PMC8062704

[150] A. Gibbs , K. Healy , V. Kaldhusdal , et al., “Preserved Mucosal‐Associated Invariant T Cells in the Cervical Mucosa of HIV‐Infected Women With Dominant Expression of the TRAV1‐2‐TRAJ20 T Cell Receptor α‐Chain,” The Journal of Infectious Diseases 226, no. 8 (2022): 1428–1440. 35511032 10.1093/infdis/jiac171 PMC9574661

[151] J. M. Brenchley , D. A. Price , T. W. Schacker , et al., “Microbial Translocation Is a Cause of Systemic Immune Activation in Chronic HIV Infection,” Nature Medicine 12, no. 12 (2006): 1365–1371. 10.1038/nm1511 17115046

[152] J. K. Sandberg , J. Dias , and B. L. Shacklett , and E. Leeansyah , “Will Loss of Your Mucosa‐associated Invariant T Cells Weaken Your HAART?,” Aids 27, no. 16 (2013): 2501–2504. 23595154 10.1097/QAD.0b013e3283620726 PMC4004624

[153] O. Rouxel , J. Da Silva , L. Beaudoin , et al., “Cytotoxic and Regulatory Roles of Mucosal‐associated Invariant T Cells in Type 1 Diabetes,” Nature Immunology 18, no. 12 (2017): 1321–1331. 28991267 10.1038/ni.3854 PMC6025738

[154] P. Xia , X. D. Xing , C. X. Yang , et al., “Activation‐induced Pyroptosis Contributes to the Loss of MAIT Cells in Chronic HIV‐1 Infected Patients,” Military Medical Research 9, no. 1 (2022): 24. 35619176 10.1186/s40779-022-00384-1 PMC9137088

[155] X. Tang , S. Zhang , Q. Peng , et al., “Sustained IFN‐I Stimulation Impairs MAIT Cell Responses to Bacteria by Inducing IL‐10 During Chronic HIV‐1 Infection,” Science Advances 6, no. 8 (2020): eaaz0374. 32128419 10.1126/sciadv.aaz0374 PMC7030930

[156] K. G. Lal , D. Kim , M. C. Costanzo , et al., “Dynamic MAIT Cell Response With Progressively Enhanced Innateness During Acute HIV‐1 Infection,” Nature Communications 11, no. 1 (2020): 272. 10.1038/s41467-019-13975-9 PMC6959336 31937782

[157] J. A. Juno , K. M. Wragg , T. Amarasena , et al., “MAIT Cells Upregulate α4β7 in Response to Acute Simian Immunodeficiency Virus/Simian HIV Infection but Are Resistant to Peripheral Depletion in Pigtail Macaques,” Journal of Immunology (Baltimore, Md: 1950) 202, no. 7 (2019): 2105–2120. 30777923 10.4049/jimmunol.1801405

[158] A. L. French , C. T. Evans , D. M. Agniel , et al., “Microbial Translocation and Liver Disease Progression in Women Coinfected With HIV and hepatitis C Virus,” The Journal of Infectious Diseases 208, no. 4 (2013): 679–689. 23687224 10.1093/infdis/jit225 PMC3719907

[159] J. Dias , E. Leeansyah , and J. K. Sandberg , “Multiple Layers of Heterogeneity and Subset Diversity in human MAIT Cell Responses to Distinct Microorganisms and to Innate Cytokines,” Proceedings of the National Academy of Sciences of the United States of America 114, no. 27 (2017): E5434–e5443. 28630305 10.1073/pnas.1705759114 PMC5502643

[160] K. Verdonck , E. González , S. Van Dooren , A. M. Vandamme , G. Vanham , and E. Gotuzzo , “Human T‐lymphotropic Virus 1: Recent Knowledge About an Ancient Infection,” The Lancet Infectious Diseases 7, no. 4 (2007): 266–281. 17376384 10.1016/S1473-3099(07)70081-6

[161] E. W. Meermeier , C. L. Zheng , J. G. Tran , et al., “Human Lung‐resident Mucosal‐associated Invariant T Cells Are Abundant, Express Antimicrobial Proteins, and Are Cytokine Responsive,” Communications Biology 5, no. 1 (2022): 942. 36085311 10.1038/s42003-022-03823-w PMC9463188

[162] T. Parrot , J. B. Gorin , A. Ponzetta , et al., “MAIT Cell Activation and Dynamics Associated With COVID‐19 Disease Severity,” Science Immunology 5, no. 51 (2020): eabe1670. 32989174 10.1126/sciimmunol.abe1670 PMC7857393

[163] H. Flament , M. Rouland , L. Beaudoin , et al., “Outcome of SARS‐CoV‐2 Infection Is Linked to MAIT Cell Activation and Cytotoxicity,” Nature Immunology 22, no. 3 (2021): 322–335. 33531712 10.1038/s41590-021-00870-z

[164] J. Youngs , N. M. Provine , N. Lim , et al., “Identification of Immune Correlates of Fatal Outcomes in Critically Ill COVID‐19 Patients,” PLoS Pathogens 17, no. 9 (2021): e1009804. 34529726 10.1371/journal.ppat.1009804 PMC8445447

[165] Y. Jouan , A. Guillon , L. Gonzalez , et al., “Phenotypical and Functional Alteration of Unconventional T Cells in Severe COVID‐19 Patients,” The Journal of Experimental Medicine 217, no. 12 (2020): e20200872. 32886755 10.1084/jem.20200872 PMC7472174

[166] P. T. Rudak , T. Yao , C. D. Richardson , and S. M. M. Haeryfar , “Measles Virus Infects and Programs MAIT Cells for Apoptosis,” The Journal of Infectious Diseases 223, no. 4 (2021): 667–672. 32623457 10.1093/infdis/jiaa407 PMC7904293

[167] J. M. Eberhard , S. Kummer , P. Hartjen , et al., “Reduced CD161(+) MAIT Cell Frequencies in HCV and HIV/HCV co‐infection: Is the Liver the Heart of the Matter?,” Journal of Hepatology 65, no. 6 (2016): 1261–1263. 27492054 10.1016/j.jhep.2016.07.031

[168] M. Spaan , S. J. Hullegie , B. J. Beudeker , et al., “Frequencies of Circulating MAIT Cells Are Diminished in Chronic HCV, HIV and HCV/HIV Co‐Infection and Do Not Recover During Therapy,” PLoS ONE 11, no. 7 (2016): e0159243. 27416100 10.1371/journal.pone.0159243 PMC4945024

[169] G. Luo , J. Zhang , T. Wang , et al., “A human Commensal‐pathogenic Fungus Suppresses Host Immunity via Targeting TBK1,” Cell Host & Microbe 32, no. 9 (2024): 1536–1551. e6. 39084229 10.1016/j.chom.2024.07.003

[170] B. Zhai , C. Liao , S. Jaggavarapu , et al., “Antifungal Heteroresistance Causes Prophylaxis Failure and Facilitates Breakthrough Candida parapsilosis Infections,” Nature Medicine 30, no. 11 (2024): 3163–3172. 10.1038/s41591-024-03183-4 PMC11840754 39095599

[171] T. Riffelmacher , M. Paynich Murray , C. Wientjens , et al., “Divergent Metabolic Programmes Control Two Populations of MAIT Cells That Protect the Lung,” Nature Cell Biology 25, no. 6 (2023): 877–891. 37231163 10.1038/s41556-023-01152-6 PMC10264248

[172] Q. Jing , R. Liu , Q. Jiang , et al., “Staphylococcus aureus Wraps Around Candida albicans and Synergistically Escapes From Neutrophil Extracellular Traps,” Frontiers in Immunology 15 (2024): 1422440. 39050841 10.3389/fimmu.2024.1422440 PMC11266059

[173] W. Awad , J. R. Mayall , W. Xu , et al., “Cigarette Smoke Components Modulate the MR1‐MAIT Axis,” The Journal of Experimental Medicine 222, no. 2 (2025): e20240896. 39820322 10.1084/jem.20240896 PMC11740918

[174] M. Mpina , N. J. Maurice , M. Yajima , et al., “Controlled Human Malaria Infection Leads to Long‐Lasting Changes in Innate and Innate‐Like Lymphocyte Populations,” Journal of Immunology (Baltimore, Md: 1950) 199, no. 1 (2017): 107–118. 28576979 10.4049/jimmunol.1601989 PMC5528886

[175] Y. G. Jo , H. M. Jin , Y. N. Cho , J. C. Kim , S. J. Kee , and Y. W. Park , “Activation and Impaired Tumor Necrosis Factor‐alpha Production of Circulating Mucosal‐Associated Invariant T Cells in Patients With Trauma,” J Innate Immun 11, no. 6 (2019): 506–515. 31085907 10.1159/000499343 PMC6758945

[176] S. J. Kang , H. M. Jin , E. J. Won , et al., “Activation, Impaired Tumor Necrosis Factor‐α Production, and Deficiency of Circulating Mucosal‐Associated Invariant T Cells in Patients With Scrub Typhus,” PLoS Neglected Tropical Diseases 10, no. 7 (2016): e0004832. 27463223 10.1371/journal.pntd.0004832 PMC4963088

[177] Y. Zhang , J. T. Bailey , E. Xu , et al., “Mucosal‐associated Invariant T Cells Restrict Reactive Oxidative Damage and Preserve Meningeal Barrier Integrity and Cognitive Function,” Nature Immunology 23, no. 12 (2022): 1714–1725. 36411380 10.1038/s41590-022-01349-1 PMC10202031

[178] M. Walkenhorst , J. K. Sonner , N. Meurs , et al., “Protective Effect of TCR‐mediated MAIT Cell Activation During Experimental Autoimmune Encephalomyelitis,” Nature Communications 15, no. 1 (2024): 9287. 10.1038/s41467-024-53657-9 PMC11519641 39468055

[179] D. De Federicis , C. Bassani , R. R. Chiarelli , et al., “Circulating MAIT Cells in Multiple Sclerosis and Amyotrophic Lateral Sclerosis,” Frontiers in Immunology 15 (2024): 1436717. 39108272 10.3389/fimmu.2024.1436717 PMC11300250

[180] A. Willing , J. Jäger , S. Reinhardt , N. Kursawe , and M. A. Friese , “Production of IL‐17 by MAIT Cells Is Increased in Multiple Sclerosis and Is Associated With IL‐7 Receptor Expression,” Journal of Immunology (Baltimore, Md: 1950) 200, no. 3 (2018): 974–982. 29298833 10.4049/jimmunol.1701213

[181] H. Koppejan , D. Jansen , M. Hameetman , R. Thomas , R. E. M. Toes , and F. A. van Gaalen , “Altered Composition and Phenotype of Mucosal‐associated Invariant T Cells in Early Untreated Rheumatoid Arthritis,” Arthritis Research & Therapy 21, no. 1 (2019): 3. 30611306 10.1186/s13075-018-1799-1 PMC6321723

[182] M. Kyriakidi , E. K. Vetsika , G. E. Fragoulis , M. Tektonidou , and P. P. Sfikakis , “Identification and Clinical Correlation of Circulating MAIT, Γδ T, ILC3, and Pre‐Inflammatory Mesenchymal Cells in Patients With Rheumatoid Arthritis and Spondyloarthritis,” Mediterranean Journal of Rheumatology 35, no. 2 (2024): 312–315. 39211026 10.31138/mjr.251022.iac PMC11350411

[183] H. J. T. Lien , T. T. Pedersen , B. Jakobsen , et al., “Single‐cell Resolution of Longitudinal Blood Transcriptome Profiles in Rheumatoid Arthritis, Systemic Lupus Erythematosus and Healthy Control Pregnancies,” Annals of the Rheumatic Diseases 83, no. 3 (2024): 300–311. 38049980 10.1136/ard-2023-224644 PMC10894842

[184] K. Abacar , T. Macleod , H. Direskeneli , and D. McGonagle , “How Underappreciated Autoinflammatory (innate immunity) Mechanisms Dominate Disparate Autoimmune Disorders,” Frontiers in Immunology 15 (2024): 1439371. 39372419 10.3389/fimmu.2024.1439371 PMC11449752

[185] Z. Wu , X. Chen , F. Han , and E. Leeansyah , “MAIT Cell Homing in Intestinal Homeostasis and Inflammation,” Science Advances 11, no. 6 (2025): eadu4172. 39919191 10.1126/sciadv.adu4172 PMC11804934

[186] V. Mitsialis , S. Wall , P. Liu , et al., “Single‐Cell Analyses of Colon and Blood Reveal Distinct Immune Cell Signatures of Ulcerative Colitis and Crohn's Disease,” Gastroenterology 159, no. 2 (2020): 591–608. e10. 32428507 10.1053/j.gastro.2020.04.074 PMC8166295

[187] M. Yuksel , F. Nazmi , D. Wardat , et al., “Standard Immunosuppressive Treatment Reduces Regulatory B Cells in Children With Autoimmune Liver Disease,” Frontiers in Immunology 13 (2022): 1053216. 36685568 10.3389/fimmu.2022.1053216 PMC9849683

[188] A. Renand , S. Habes , J. F. Mosnier , et al., “Immune Alterations in Patients with Type 1 Autoimmune Hepatitis Persist Upon Standard Immunosuppressive Treatment,” Hepatology Communications 2, no. 8 (2018): 968–981. 30094407 10.1002/hep4.1202 PMC6078209

[189] Y. N. Cho , S. J. Kee , T. J. Kim , et al., “Mucosal‐associated Invariant T Cell Deficiency in Systemic Lupus Erythematosus,” Journal of Immunology (Baltimore, Md: 1950) 193, no. 8 (2014): 3891–3901. 25225673 10.4049/jimmunol.1302701

[190] E. Litvinova , C. Bounaix , G. Hanouna , et al., “MAIT Cells Altered Phenotype and Cytotoxicity in lupus Patients Are Linked to Renal Disease Severity and Outcome,” Frontiers in Immunology 14 (2023): 1205405. 37885889 10.3389/fimmu.2023.1205405 PMC10598677

[191] E. Gülden , N. Palm , and K. C. Herold , “MAIT Cells: A Link Between Gut Integrity and Type 1 Diabetes,” Cell Metabolism 26, no. 6 (2017): 813–815. 29211981 10.1016/j.cmet.2017.11.007

[192] M. Shimamura , Y. Y. Huang , H. Goji , S. Endo , R. Migishima , and M. Yokoyama , “Regulation of Immunological Disorders by Invariant Vα19‐Jα33 TCR‐bearing Cells,” Immunobiology 216, no. 3 (2011): 374–378. 20832138 10.1016/j.imbio.2010.08.003

[193] M. Sadeghi , S. Dehnavi , A. M. Amiri , A. E. Butler , and A. Sahebkar , “Mucosal‐associated Invariant T Cells in Diabetes Mellitus: Protective or Destructive?,” International Immunopharmacology 161 (2025): 115069. 40499469 10.1016/j.intimp.2025.115069

[194] K. E. de Visser and J. A. Joyce , “The Evolving Tumor Microenvironment: From Cancer Initiation to Metastatic Outgrowth,” Cancer Cell 41, no. 3 (2023): 374–403. 36917948 10.1016/j.ccell.2023.02.016

[195] Y. R. Li , K. Zhou , M. Wilson , et al., “Mucosal‐associated Invariant T Cells for Cancer Immunotherapy,” Molecular Therapy: the Journal of the American Society of Gene Therapy 31, no. 3 (2023): 631–646. 36463401 10.1016/j.ymthe.2022.11.019 PMC10014234

[196] W. C. Huang , Y. C. Hsiao , C. C. Wu , Y. T. Hsu , and C. L. Chang , “Less Circulating Mucosal‐associated Invariant T Cells in Patients With Cervical Cancer,” Taiwanese Journal of Obstetrics & Gynecology 58, no. 1 (2019): 117–121. 30638464 10.1016/j.tjog.2018.11.022

[197] P. Sundström , L. Szeponik , F. Ahlmanner , et al., “Tumor‐infiltrating Mucosal‐associated Invariant T (MAIT) Cells Retain Expression of Cytotoxic Effector Molecules,” Oncotarget 10, no. 29 (2019): 2810–2823. 31073372 10.18632/oncotarget.26866 PMC6497460

[198] C. Shao , C. Zhu , Y. Zhu , et al., “Decrease of Peripheral Blood Mucosal‐associated Invariant T Cells and Impaired Serum Granzyme‐B Production in Patients With Gastric Cancer,” Cell & Bioscience 11, no. 1 (2021): 12. 33422137 10.1186/s13578-020-00518-9 PMC7796455

[199] W. Rodin , P. Sundström , F. Ahlmanner , et al., “Exhaustion in Tumor‐infiltrating Mucosal‐Associated Invariant T (MAIT) Cells From Colon Cancer Patients,” Cancer Immunology, Immunotherapy: CII 70, no. 12 (2021): 3461–3475. 33885944 10.1007/s00262-021-02939-y PMC8571139

[200] M. Duan , S. Goswami , J. Y. Shi , et al., “Activated and Exhausted MAIT Cells Foster Disease Progression and Indicate Poor Outcome in Hepatocellular Carcinoma,” Clinical Cancer Research: an Official Journal of the American Association for Cancer Research 25, no. 11 (2019): 3304–3316. 30723143 10.1158/1078-0432.CCR-18-3040

[201] J. Yan , S. Allen , E. McDonald , et al., “MAIT Cells Promote Tumor Initiation, Growth, and Metastases via Tumor MR1,” Cancer Discovery 10, no. 1 (2020): 124–141. 31826876 10.1158/2159-8290.CD-19-0569

[202] Q. Zhang , P. Li , W. Zhou , S. Fang , and J. Wang , “Participation of Increased Circulating MAIT Cells in Lung Cancer: A Pilot Study,” Journal of Cancer 13, no. 5 (2022): 1623–1629. 35371315 10.7150/jca.69415 PMC8965108

[203] C. Zheng , L. Zheng , J. K. Yoo , et al., “Landscape of Infiltrating T Cells in Liver Cancer Revealed by Single‐Cell Sequencing,” Cell 169, no. 7 (2017): 1342–1356. e16. 28622514 10.1016/j.cell.2017.05.035

[204] C. R. Shaler , M. E. Tun‐Abraham , A. I. Skaro , et al., “Mucosa‐associated Invariant T Cells Infiltrate Hepatic Metastases in Patients With Colorectal Carcinoma but Are Rendered Dysfunctional Within and Adjacent to Tumor Microenvironment,” Cancer Immunology, Immunotherapy 66, no. 12 (2017): 1563–1575. 28798979 10.1007/s00262-017-2050-7 PMC11029177

[205] C. L. Zimmer , I. Filipovic , M. Cornillet , et al., “Mucosal‐associated Invariant T‐cell Tumor Infiltration Predicts Long‐term Survival in Cholangiocarcinoma,” Hepatology 75, no. 5 (2022): 1154–1168. 34719787 10.1002/hep.32222

[206] L. Ling , Y. Lin , W. Zheng , et al., “Circulating and Tumor‐infiltrating Mucosal Associated Invariant T (MAIT) Cells in Colorectal Cancer Patients,” Scientific Reports 6, no. 1 (2016): 20358. 26837580 10.1038/srep20358 PMC4738248

[207] P. Sundström , F. Ahlmanner , P. Akéus , et al., “Human Mucosa‐Associated Invariant T Cells Accumulate in Colon Adenocarcinomas but Produce Reduced Amounts of IFN‐γ,” Journal of Immunology (Baltimore, Md: 1950) 195, no. 7 (2015): 3472–3481. 26297765 10.4049/jimmunol.1500258

[208] L. Zabijak , C. Attencourt , C. Guignant , et al., “Increased Tumor Infiltration by Mucosal‐associated Invariant T Cells Correlates With Poor Survival in Colorectal Cancer Patients,” Cancer Immunology, Immunotherapy 64, no. 12 (2015): 1601–1608. 26497850 10.1007/s00262-015-1764-7 PMC11028701

[209] A. M. Melo , A. M. O'Brien , and J. J. Phelan , “Mucosal‐Associated Invariant T Cells Display Diminished Effector Capacity in Oesophageal Adenocarcinoma,” Frontiers in Immunology 10 (2019): 1580. 31354725 10.3389/fimmu.2019.01580 PMC6635552

[210] J. Qu , B. Wu , L. Chen , et al., “CXCR6‐positive Circulating Mucosal‐associated Invariant T Cells Can Identify Patients With Non‐small Cell Lung Cancer Responding to anti‐PD‐1 Immunotherapy,” Journal of Experimental & Clinical Cancer Research: CR 43, no. 1 (2024): 134. 38698468 10.1186/s13046-024-03046-3 PMC11067263

[211] T. Comont , M. L. Nicolau‐Travers , S. Bertoli , C. Recher , F. Vergez , and E. Treiner , “MAIT Cells Numbers and Frequencies in Patients With Acute Myeloid Leukemia at Diagnosis: Association With Cytogenetic Profile and Gene Mutations,” Cancer Immunology, Immunotherapy: CII 71, no. 4 (2022): 875–887. 34477901 10.1007/s00262-021-03037-9 PMC10991316

[212] W. Wu , X. Liang , H. Li , et al., “Landscape of T Cells in NK‐AML(M4/M5) Revealed by Single‐Cell Sequencing,” Journal of Leukocyte Biology 112, no. 4 (2022): 745–758. 35258858 10.1002/JLB.5A0721-396RR

[213] M. Favreau , K. Venken , S. Faict , et al., “Both Mucosal‐associated Invariant and Natural Killer T‐cell Deficiency in Multiple Myeloma Can be Countered by PD‐1 Inhibition,” Haematologica 102, no. 7 (2017): e266–e270. 28385777 10.3324/haematol.2017.163758 PMC5566052

[214] R. L. Siegel , K. D. Miller , N. S. Wagle , and A. Jemal , “Cancer Statistics, 2023,” CA: A Cancer Journal for Clinicians 73, no. 1 (2023): 17–48. 36633525 10.3322/caac.21763

[215] I. Afanas'ev , “New Nucleophilic Mechanisms of Ros‐dependent Epigenetic Modifications: Comparison of Aging and Cancer,” Aging and Disease 5, no. 1 (2014): 52–62. 24490117 10.14336/AD.2014.050052 PMC3901614

[216] Y. Yin , A. Zeng , A. Abuduwayiti , et al., “MAIT Cells Are Associated With Responsiveness to Neoadjuvant Immunotherapy in COPD‐associated NSCLC,” Cancer Medicine 13, no. 6 (2024): e7112. 38509769 10.1002/cam4.7112 PMC10955227

[217] L. Shi , J. Lu , D. Zhong , et al., “Clinicopathological and Predictive Value of MAIT Cells in Non‐small Cell Lung Cancer for Immunotherapy,” Journal for Immunotherapy of Cancer 11, no. 1 (2023): e005902. 36657812 10.1136/jitc-2022-005902 PMC9853268

[218] L. Ouyang , M. Wu , J. Zhao , et al., “Mucosal‐associated Invariant T Cells Reduce and Display Tissue‐resident Phenotype With Elevated IL‐17 Producing Capacity in Non‐small Cell Lung Cancer,” International Immunopharmacology 113, no. Pt B (2022): 109461. 36435063 10.1016/j.intimp.2022.109461

[219] P. Sundström , N. Dutta , W. Rodin , A. Hallqvist , S. Raghavan , and M. Quiding Järbrink , “Immune Checkpoint Blockade Improves the Activation and Function of Circulating Mucosal‐associated Invariant T (MAIT) Cells in Patients With Non‐small Cell Lung Cancer,” Oncoimmunology 13, no. 1 (2024): 2312631. 38343750 10.1080/2162402X.2024.2312631 PMC10854269

[220] J. W. Vardiman , J. Thiele , D. A. Arber , et al., “The 2008 Revision of the World Health Organization (WHO) Classification of Myeloid Neoplasms and Acute Leukemia: Rationale and Important Changes,” Blood 114, no. 5 (2009): 937–951. 19357394 10.1182/blood-2009-03-209262

[221] N. Bozorgmehr , M. Hnatiuk , A. C. Peters , and S. Elahi , “Depletion of Polyfunctional CD26(high)CD8(+) T Cells Repertoire in Chronic Lymphocytic Leukemia,” Experimental Hematology & Oncology 12, no. 1 (2023): 13. 36707896 10.1186/s40164-023-00375-5 PMC9881277

[222] P. K. Sharma , E. B. Wong , R. J. Napier , et al., “High Expression of CD26 Accurately Identifies human Bacteria‐reactive MR1‐restricted MAIT Cells,” Immunology 145, no. 3 (2015): 443–453. 25752900 10.1111/imm.12461 PMC4479542

[223] E. M. Jarvis , S. Collings , A. Authier‐Hall , et al., “Mucosal‐Associated Invariant T (MAIT) Cell Dysfunction and PD‐1 Expression in Prostate Cancer: Implications for Immunotherapy,” Frontiers in Immunology 12 (2021): 748741. 34737749 10.3389/fimmu.2021.748741 PMC8560687

[224] A. Bhattacharyya , L. A. Hanafi , A. Sheih , et al., “Graft‐Derived Reconstitution of Mucosal‐Associated Invariant T Cells After Allogeneic Hematopoietic Cell Transplantation,” Biology of Blood and Marrow Transplantation 24, no. 2 (2018): 242–251. 29024803 10.1016/j.bbmt.2017.10.003 PMC5806215

[225] H. Andrlová , O. Miltiadous , A. I. Kousa , et al., “MAIT and Vδ2 Unconventional T Cells Are Supported by a Diverse Intestinal Microbiome and Correlate With Favorable Patient Outcome After Allogeneic HCT,” Science Translational Medicine 14, no. 646 (2022): eabj2829. 35613281 10.1126/scitranslmed.abj2829 PMC9893439

[226] M. G. Gao , Y. Hong , X. Y. Zhao , et al., “The Potential Roles of Mucosa‐Associated Invariant T Cells in the Pathogenesis of Gut Graft‐Versus‐Host Disease after Hematopoietic Stem Cell Transplantation,” Frontiers in Immunology 12 (2021): 720354. 34539656 10.3389/fimmu.2021.720354 PMC8448388

[227] J. Novak , J. Dobrovolny , J. Brozova , L. Novakova , and T. Kozak , “Recovery of Mucosal‐associated Invariant T Cells After Myeloablative Chemotherapy and Autologous Peripheral Blood Stem Cell Transplantation,” Clinical and Experimental Medicine 16, no. 4 (2016): 529–537. 26409838 10.1007/s10238-015-0384-z

[228] K. Kawaguchi , K. Umeda , E. Hiejima , et al., “Influence of Post‐transplant Mucosal‐associated Invariant T Cell Recovery on the Development of Acute Graft‐versus‐host Disease in Allogeneic Bone Marrow Transplantation,” International Journal of Hematology 108, no. 1 (2018): 66–75. 29582333 10.1007/s12185-018-2442-2

[229] J. M. Llovet , R. K. Kelley , A. Villanueva , et al., “Hepatocellular Carcinoma,” Nature Reviews Disease Primers 7, no. 1 (2021): 6. 10.1038/s41572-020-00240-3 PMC13537433 33479224

[230] J. M. Llovet , F. Castet , M. Heikenwalder , et al., “Immunotherapies for Hepatocellular Carcinoma,” Nature Reviews Clinical Oncology 19, no. 3 (2022): 151–172. 10.1038/s41571-021-00573-2 34764464

[231] L. Lu , J. Jiang , M. Zhan , et al., “Targeting Tumor‐Associated Antigens in Hepatocellular Carcinoma for Immunotherapy: Past Pitfalls and Future Strategies,” Hepatology (Baltimore, Md) 73, no. 2 (2021): 821–832. 10.1002/hep.31502 32767586

[232] T. Yao , P. Shooshtari , and S. M. M. Haeryfar , “Leveraging Public Single‐Cell and Bulk Transcriptomic Datasets to Delineate MAIT Cell Roles and Phenotypic Characteristics in Human Malignancies,” Frontiers in Immunology 11 (2020): 1691. 32849590 10.3389/fimmu.2020.01691 PMC7413026

[233] W. Huang , D. Ye , W. He , X. He , X. Shi , and Y. Gao , “Activated but Impaired IFN‐γ Production of Mucosal‐associated Invariant T Cells in Patients With Hepatocellular Carcinoma,” Journal for Immunotherapy of Cancer 9, no. 11 (2021): e003685. 34789552 10.1136/jitc-2021-003685 PMC8601081

[234] H. Wang , L. Kjer‐Nielsen , M. Shi , et al., “IL‐23 Costimulates Antigen‐specific MAIT Cell Activation and Enables Vaccination Against Bacterial Infection,” Science Immunology 4 (2019): eaaw0402. 31732518 10.1126/sciimmunol.aaw0402

[235] B. Ruf , M. Bruhns , S. Babaei , et al., “Tumor‐associated Macrophages Trigger MAIT Cell Dysfunction at the HCC Invasive Margin,” Cell 186, no. 17 (2023): 3686–3705. e32. 37595566 10.1016/j.cell.2023.07.026 PMC10461130

[236] S. Fu , M. Liu , C. Zhu , et al., “Regulatory Mucosa‐associated Invariant T Cells Controlled by β1 Adrenergic Receptor Signaling Contribute to Hepatocellular Carcinoma Progression,” Hepatology (Baltimore, Md) 78, no. 1 (2023): 72–87. 10.1097/HEP.0000000000000014 36626624

[237] S. Rizvi , S. A. Khan , C. L. Hallemeier , R. K. Kelley , and G. J. Gores , “Cholangiocarcinoma—evolving Concepts and Therapeutic Strategies,” Nature Reviews Clinical Oncology 15, no. 2 (2018): 95–111. 10.1038/nrclinonc.2017.157 PMC5819599 28994423

[238] P. J. Brindley , M. Bachini , S. I. Ilyas , et al., “Cholangiocarcinoma,” Nature Reviews Disease Primers 7, no. 1 (2021): 65. 10.1038/s41572-021-00300-2 PMC9246479 34504109

[239] H. Sung , J. Ferlay , R. L. Siegel , et al., “Global Cancer Statistics 2020: GLOBOCAN Estimates of Incidence and Mortality Worldwide for 36 Cancers in 185 Countries,” CA: A Cancer Journal for Clinicians 71, no. 3 (2021): 209–249. 33538338 10.3322/caac.21660

[240] M. C. S. Wong , J. Huang , V. Lok , et al., “Differences in Incidence and Mortality Trends of Colorectal Cancer Worldwide Based on Sex, Age, and Anatomic Location,” Clinical Gastroenterology and Hepatology 19, no. 5 (2021): 955–966. e61. 32088300 10.1016/j.cgh.2020.02.026

[241] M. S. Islam , V. Gopalan , A. K. Lam , and M. J. A. Shiddiky , “Current Advances in Detecting Genetic and Epigenetic Biomarkers of Colorectal Cancer,” Biosensors & Bioelectronics 239 (2023): 115611. 37619478 10.1016/j.bios.2023.115611

[242] M. Schmitt and F. R. Greten , “The Inflammatory Pathogenesis of Colorectal Cancer,” Nature Reviews Immunology 21, no. 10 (2021): 653–667. 10.1038/s41577-021-00534-x 33911231

[243] I. J. ME , R. Sanz‐Pamplona , F. Hermitte , and N. de Miranda , “Colorectal Cancer: A Paradigmatic Model for Cancer Immunology and Immunotherapy,” Molecular Aspects of Medicine 69 (2019): 123–129. 31136750 10.1016/j.mam.2019.05.003

[244] T. A. Wynn , “IL‐13 Effector Functions,” Annual Review of Immunology 21 (2003): 425–456. 10.1146/annurev.immunol.21.120601.141142 12615888

[245] M. Jaén , Á. Martín‐Regalado , R. A. Bartolomé , J. Robles , and J. I. Casal , “Interleukin 13 Receptor Alpha 2 (IL13Rα2): Expression, Signaling Pathways and Therapeutic Applications in Cancer,” Biochimica Et Biophysica Acta Reviews on Cancer 1877, no. 5 (2022): 188802. 36152905 10.1016/j.bbcan.2022.188802

[246] J. Kelly , Y. Minoda , T. Meredith , et al., “Chronically Stimulated human MAIT Cells Are Unexpectedly Potent IL‐13 Producers,” Immunology and Cell Biology 97, no. 8 (2019): 689–699. 31323167 10.1111/imcb.12281 PMC6790710

[247] A. Tanaka and S. Sakaguchi , “Regulatory T Cells in Cancer Immunotherapy,” Cell Research 27, no. 1 (2017): 109–118. 27995907 10.1038/cr.2016.151 PMC5223231

[248] Y. Liu , Q. Zhang , B. Xing , et al., “Immune Phenotypic Linkage Between Colorectal Cancer and Liver Metastasis,” Cancer Cell 40, no. 4 (2022): 424–437. e5. 35303421 10.1016/j.ccell.2022.02.013

[249] C. K. Vorkas , C. Krishna , K. Li , et al., “Single‐Cell Transcriptional Profiling Reveals Signatures of Helper, Effector, and Regulatory MAIT Cells During Homeostasis and Activation,” Journal of Immunology (Baltimore, Md: 1950) 208, no. 5 (2022): 1042–1056. 35149530 10.4049/jimmunol.2100522 PMC9012082

[250] G. Churlaud , F. Pitoiset , F. Jebbawi , et al., “Human and Mouse CD8(+)CD25(+)FOXP3(+) Regulatory T Cells at Steady State and During Interleukin‐2 Therapy,” Frontiers in Immunology 6 (2015): 171. 25926835 10.3389/fimmu.2015.00171 PMC4397865

[251] S. Li , Y. Simoni , E. Becht , et al., “Human Tumor‐Infiltrating MAIT Cells Display Hallmarks of Bacterial Antigen Recognition in Colorectal Cancer,” Cell Reports Medicine 1, no. 3 (2020): 100039. 33205061 10.1016/j.xcrm.2020.100039 PMC7659584

[252] E. Morgan , I. Soerjomataram , H. Rumgay , et al., “The Global Landscape of Esophageal Squamous Cell Carcinoma and Esophageal Adenocarcinoma Incidence and Mortality in 2020 and Projections to 2040: New Estimates from GLOBOCAN 2020,” Gastroenterology 163, no. 3 (2022): 649–658. e2. 35671803 10.1053/j.gastro.2022.05.054

[253] C. T. Demarest and A. C. Chang , “The Landmark Series: Multimodal Therapy for Esophageal Cancer,” Annals of Surgical Oncology 28, no. 6 (2021): 3375–3382. 33629251 10.1245/s10434-020-09565-5

[254] G. Luo , Y. Zhang , P. Guo , L. Wang , Y. Huang , and K. Li , “Global Patterns and Trends in Stomach Cancer Incidence: Age, Period and Birth Cohort Analysis,” International Journal of Cancer 141, no. 7 (2017): 1333–1344. 28614909 10.1002/ijc.30835

[255] R. Wang , S. Song , J. Qin , et al., “Evolution of Immune and Stromal Cell States and Ecotypes During Gastric Adenocarcinoma Progression,” Cancer Cell 41, no. 8 (2023): 1407–1426. e9. 37419119 10.1016/j.ccell.2023.06.005 PMC10528152

[256] H. Jiang , D. Yu , P. Yang , et al., “Revealing the Transcriptional Heterogeneity of Organ‐specific Metastasis in human Gastric Cancer Using Single‐cell RNA Sequencing,” Clinical and Translational Medicine 12, no. 2 (2022): e730. 35184420 10.1002/ctm2.730 PMC8858624

[257] C. D'Souza , T. Pediongco , and H. Wang , “Mucosal‐Associated Invariant T Cells Augment Immunopathology and Gastritis in Chronic Helicobacter pylori Infection,” Journal of Immunology (Baltimore, Md: 1950) 200, no. 5 (2018): 1901–1916. 29378910 10.4049/jimmunol.1701512

[258] S. Ming , M. Zhang , Z. Liang , et al., “OX40L/OX40 Signal Promotes IL‐9 Production by Mucosal MAIT Cells during Helicobacter pylori Infection,” Frontiers in Immunology 12 (2021): 626017. 33777009 10.3389/fimmu.2021.626017 PMC7990886

[259] S. R. Hingorani , “Epithelial and Stromal co‐evolution and Complicity in Pancreatic Cancer,” Nature Reviews Cancer 23, no. 2 (2023): 57–77. 36446904 10.1038/s41568-022-00530-w PMC10470605

[260] B. Ren , M. Cui , G. Yang , et al., “Tumor Microenvironment Participates in Metastasis of Pancreatic Cancer,” Molecular Cancer 17, no. 1 (2018): 108. 30060755 10.1186/s12943-018-0858-1 PMC6065152

[261] E. Kuric , L. Krogvold , K. F. Hanssen , K. Dahl‐Jørgensen , O. Skog , and O. Korsgren , “No Evidence for Presence of Mucosal‐Associated Invariant T Cells in the Insulitic Lesions in Patients Recently Diagnosed With Type 1 Diabetes,” The American Journal of Pathology 188, no. 8 (2018): 1744–1748. 29803829 10.1016/j.ajpath.2018.04.009

[262] A. Lehuen , J. Diana , P. Zaccone , and A. Cooke , “Immune Cell Crosstalk in Type 1 Diabetes,” Nature Reviews Immunology 10, no. 7 (2010): 501–513. 10.1038/nri2787 20577267

[263] J. A. Bluestone , K. Herold , and G. Eisenbarth , “Genetics, Pathogenesis and Clinical Interventions in Type 1 Diabetes,” Nature 464, no. 7293 (2010): 1293–1300. 20432533 10.1038/nature08933 PMC4959889

[264] I. Magalhaes , K. Pingris , C. Poitou , et al., “Mucosal‐associated Invariant T Cell Alterations in Obese and Type 2 Diabetic Patients,” The Journal of Clinical Investigation 125, no. 4 (2015): 1752–1762. 25751065 10.1172/JCI78941 PMC4396481

[265] E. Carolan , L. M. Tobin , and B. A. Mangan , “Altered Distribution and Increased IL‐17 Production by Mucosal‐associated Invariant T Cells in Adult and Childhood Obesity,” Journal of Immunology (Baltimore, Md: 1950) 194, no. 12 (2015): 5775–5780. 25980010 10.4049/jimmunol.1402945

[266] L. Chiossone , P. Y. Dumas , M. Vienne , and E. Vivier , “Natural Killer Cells and Other Innate Lymphoid Cells in Cancer,” Nature Reviews Immunology 18, no. 11 (2018): 671–688. 10.1038/s41577-018-0061-z 30209347

[267] N. D. Huntington , J. Cursons , and J. Rautela , “The Cancer‐natural Killer Cell Immunity Cycle,” Nature Reviews Cancer 20, no. 8 (2020): 437–454. 32581320 10.1038/s41568-020-0272-z

[268] E. V. Petley , H. F. Koay , M. A. Henderson , et al., “MAIT Cells Regulate NK Cell‐mediated Tumor Immunity,” Nature Communications 12, no. 1 (2021): 4746. 10.1038/s41467-021-25009-4 PMC8346465 34362900

[269] L. Cassetta and J. W. Pollard , “A Timeline of Tumour‐associated Macrophage Biology,” Nature Reviews Cancer 23, no. 4 (2023): 238–257. 36792751 10.1038/s41568-022-00547-1

[270] H. Wang , L. Kjer‐Nielsen , M. Shi , et al., “IL‐23 Costimulates Antigen‐specific MAIT Cell Activation and Enables Vaccination Against Bacterial Infection,” Science Immunology 4, no. 41 (2019): eaaw0402. 31732518 10.1126/sciimmunol.aaw0402

[271] Y. Li , B. Huang , X. Jiang , et al., “Mucosal‐Associated Invariant T Cells Improve Nonalcoholic Fatty Liver Disease through Regulating Macrophage Polarization,” Frontiers in Immunology 9 (2018): 1994. 30233587 10.3389/fimmu.2018.01994 PMC6131560

[272] M. Mabire , P. Hegde , A. Hammoutene , et al., “MAIT Cell Inhibition Promotes Liver Fibrosis Regression via Macrophage Phenotype Reprogramming,” Nature Communications 14, no. 1 (2023): 1830. 10.1038/s41467-023-37453-5 PMC10067815 37005415

[273] C. M. Laumont , A. C. Banville , M. Gilardi , D. P. Hollern , and B. H. Nelson , “Tumour‐infiltrating B Cells: Immunological Mechanisms, Clinical Impact and Therapeutic Opportunities,” Nature Reviews Cancer 22, no. 7 (2022): 414–430. 35393541 10.1038/s41568-022-00466-1 PMC9678336

[274] R. Salerno‐Goncalves , T. Rezwan , and M. B. Sztein , “B Cells Modulate Mucosal Associated Invariant T Cell Immune Responses,” Frontiers in Immunology 4 (2014): 511. 24432025 10.3389/fimmu.2013.00511 PMC3882667

[275] R. Lamichhane and J. E. Ussher , “Expression and Trafficking of MR1,” Immunology 151, no. 3 (2017): 270–279. 28419492 10.1111/imm.12744 PMC5461101

[276] G. Murayama , A. Chiba , H. Suzuki , et al., “A Critical Role for Mucosal‐Associated Invariant T Cells as Regulators and Therapeutic Targets in Systemic Lupus Erythematosus,” Frontiers in immunology 10 (2019): 2681. 31849932 10.3389/fimmu.2019.02681 PMC6895065

[277] M. S. Bennett , S. Trivedi , A. S. Iyer , J. S. Hale , and D. T. Leung , “Human Mucosal‐associated Invariant T (MAIT) Cells Possess Capacity for B Cell Help,” Journal of Leukocyte Biology 102, no. 5 (2017): 1261–1269. 28807929 10.1189/jlb.4A0317-116R PMC5636046

[278] S. J. Pedersen and W. P. Maksymowych , “The Pathogenesis of Ankylosing Spondylitis: An Update,” Current Rheumatology Reports 21, no. 10 (2019): 58. 31712904 10.1007/s11926-019-0856-3

[279] I. Sanz and F. E. Lee , “B Cells as Therapeutic Targets in SLE,” Nature Reviews Rheumatology 6, no. 6 (2010): 326–337. 20520647 10.1038/nrrheum.2010.68 PMC3934759

[280] B. R. Blazar , W. J. Murphy , and M. Abedi , “Advances in Graft‐versus‐host Disease Biology and Therapy,” Nature Reviews Immunology 12, no. 6 (2012): 443–458. 10.1038/nri3212 PMC3552454 22576252

[281] Z. G. Fridlender , J. Sun , S. Kim , et al., “Polarization of Tumor‐associated Neutrophil Phenotype by TGF‐beta: “N1” versus “N2” TAN,” Cancer Cell 16, no. 3 (2009): 183–194. 19732719 10.1016/j.ccr.2009.06.017 PMC2754404

[282] M. S. Davey , M. P. Morgan , A. R. Liuzzi , et al., “Microbe‐specific Unconventional T Cells Induce human Neutrophil Differentiation Into Antigen Cross‐presenting Cells,” Journal of Immunology (Baltimore, Md: 1950) 193, no. 7 (2014): 3704–3716. 25165152 10.4049/jimmunol.1401018 PMC4169984

[283] M. Schneider , R. F. Hannaway , R. Lamichhane , et al., “Neutrophils Suppress Mucosal‐associated Invariant T Cells in Humans,” European Journal of Immunology 50, no. 5 (2020): 643–655. 31944287 10.1002/eji.201948394

[284] M. E. Zinser , A. J. Highton , A. Kurioka , et al., “Human MAIT Cells Show Metabolic Quiescence With Rapid Glucose‐dependent Upregulation of Granzyme B Upon Stimulation,” Immunology and Cell Biology 96, no. 6 (2018): 666–674. 29423939 10.1111/imcb.12020 PMC6055666

[285] A. O'Brien , R. M. Loftus , M. M. Pisarska , et al., “Obesity Reduces mTORC1 Activity in Mucosal‐Associated Invariant T Cells, Driving Defective Metabolic and Functional Responses,” Journal of Immunology (Baltimore, Md: 1950) 202, no. 12 (2019): 3404–3411. 31076528 10.4049/jimmunol.1801600

[286] E. Treiner , “Mucosal‐associated Invariant T Cells in Inflammatory Bowel Diseases: Bystanders, Defenders, or Offenders?,” Frontiers in Immunology 6 (2015): 27. 25699045 10.3389/fimmu.2015.00027 PMC4313715

[287] C. R. Shaler , M. E. Tun‐Abraham , A. I. Skaro , et al., “Mucosa‐associated Invariant T Cells Infiltrate Hepatic Metastases in Patients With Colorectal Carcinoma but Are Rendered Dysfunctional Within and Adjacent to Tumor Microenvironment,” Cancer Immunology, Immunotherapy: CII 66, no. 12 (2017): 1563–1575. 28798979 10.1007/s00262-017-2050-7 PMC11029177

[288] A. N. Keller , S. B. Eckle , W. Xu , et al., “Drugs and Drug‐Like Molecules Can Modulate the Function of Mucosal‐associated Invariant T Cells,” Nature Immunology 18, no. 4 (2017): 402–411. 28166217 10.1038/ni.3679

[289] L. Zabijak , C. Attencourt , C. Guignant , et al., “Increased Tumor Infiltration by Mucosal‐associated Invariant T Cells Correlates With Poor Survival in Colorectal Cancer Patients,” Cancer Immunology, Immunotherapy: CII 64, no. 12 (2015): 1601–1608. 26497850 10.1007/s00262-015-1764-7 PMC11028701

[290] M. Lepore , A. Kalinichenko , S. Calogero , et al., “Functionally Diverse human T Cells Recognize Non‐microbial Antigens Presented by MR1,” Elife 6 (2017): e24476. 28518056 10.7554/eLife.24476 PMC5459576

[291] H. J. Gober , M. Kistowska , L. Angman , P. Jenö , L. Mori , and G. De Libero , “Human T Cell Receptor Gammadelta Cells Recognize Endogenous Mevalonate Metabolites in Tumor Cells,” The Journal of Experimental Medicine 197, no. 2 (2003): 163–168. 12538656 10.1084/jem.20021500 PMC2193814

[292] H. F. Koay , N. A. Gherardin , C. Xu , et al., “Diverse MR1‐restricted T Cells in Mice and Humans,” Nature Communications 10, no. 1 (2019): 2243. 10.1038/s41467-019-10198-w PMC6529461 31113973

[293] R. Lamichhane , M. Schneider , S. M. de la Harpe , et al., “TCR‐ or Cytokine‐Activated CD8(+) Mucosal‐Associated Invariant T Cells Are Rapid Polyfunctional Effectors That Can Coordinate Immune Responses,” Cell Reports 28, no. 12 (2019): 3061–3076. e5. 31533031 10.1016/j.celrep.2019.08.054

[294] L. C. Garner , A. Amini , M. E. B. FitzPatrick , et al., “Single‐cell Analysis of human MAIT Cell Transcriptional, Functional and Clonal Diversity,” Nature Immunology 24, no. 9 (2023): 1565–1578. 37580605 10.1038/s41590-023-01575-1 PMC10457204

[295] L. Camard , T. Stephen , H. Yahia‐Cherbal , et al., “IL‐23 Tunes Inflammatory Functions of human Mucosal‐associated Invariant T Cells,” Iscience 28, no. 2 (2025): 111898. 40008359 10.1016/j.isci.2025.111898 PMC11850163

[296] C. Boulouis , E. Mouchtaridi , T. R. Müller , et al., “Human MAIT Cell Response Profiles Biased Toward IL‐17 or IL‐10 Are Distinct Effector States Directed by the Cytokine Milieu,” Proceedings of the National Academy of Sciences of the United States of America 122, no. 6 (2025): e2414230122. 39903121 10.1073/pnas.2414230122 PMC11831165

[297] W. J. Chua , S. M. Truscott , C. S. Eickhoff , A. Blazevic , D. F. Hoft , and T. H. Hansen , “Polyclonal Mucosa‐associated Invariant T Cells Have Unique Innate Functions in Bacterial Infection,” Infection and Immunity 80, no. 9 (2012): 3256–3267. 22778103 10.1128/IAI.00279-12 PMC3418730

[298] S. Suliman , M. Murphy , M. Musvosvi , et al., “MR1‐Independent Activation of Human Mucosal‐Associated Invariant T Cells by Mycobacteria,” Journal of Immunology (Baltimore, Md: 1950) 203, no. 11 (2019): 2917–2927. 31611259 10.4049/jimmunol.1900674 PMC6859375

[299] A. Kurioka , B. van Wilgenburg , R. R. Javan , et al., “Diverse Streptococcus pneumoniae Strains Drive a Mucosal‐Associated Invariant T‐Cell Response through Major Histocompatibility Complex Class I‐Related Molecule‐Dependent and Cytokine‐Driven Pathways,” The Journal of Infectious Diseases 217, no. 6 (2018): 988–999. 29267892 10.1093/infdis/jix647 PMC5854017

[300] R. Lamichhane , H. Galvin , R. F. Hannaway , et al., “Type I Interferons Are Important co‐stimulatory Signals During T Cell Receptor Mediated human MAIT Cell Activation,” European Journal of Immunology 50, no. 2 (2020): 178–191. 31608441 10.1002/eji.201948279

[301] J. C. López‐Rodríguez , S. J. Hancock , K. Li , et al., “Type I Interferons Drive MAIT Cell Functions Against Bacterial Pneumonia,” The Journal of Experimental Medicine 220, no. 10 (2023): e20230037. 37516912 10.1084/jem.20230037 PMC10373297

[302] A. Sattler , C. Dang‐Heine , P. Reinke , and N. Babel , “IL‐15 Dependent Induction of IL‐18 Secretion as a Feedback Mechanism Controlling human MAIT‐cell Effector Functions,” European Journal of Immunology 45, no. 8 (2015): 2286–2298. 26046663 10.1002/eji.201445313

[303] H. Tao , Y. Pan , S. Chu , et al., “Differential Controls of MAIT Cell Effector Polarization by mTORC1/mTORC2 via Integrating Cytokine and Costimulatory Signals,” Nature Communications 12, no. 1 (2021): 2029. 10.1038/s41467-021-22162-8 PMC8016978 33795689

[304] F. Han , M. Y. Gulam , Y. Zheng , et al., “IL7RA single Nucleotide Polymorphisms Are Associated With the Size and Function of the MAIT Cell Population in Treated HIV‐1 Infection,” Frontiers in Immunology 13 (2022): 985385. 36341446 10.3389/fimmu.2022.985385 PMC9632172

[305] Q. Yang , Y. Wen , F. Qi , et al., “Suppressive Monocytes Impair MAIT Cells Response via IL‐10 in Patients With Severe COVID‐19,” Journal of Immunology (Baltimore, Md: 1950) 207, no. 7 (2021): 1848–1856. 34452933 10.4049/jimmunol.2100228

[306] K. Haga , A. Chiba , T. Shibuya , et al., “MAIT Cells Are Activated and Accumulated in the Inflamed Mucosa of Ulcerative Colitis,” Journal of Gastroenterology and Hepatology 31, no. 5 (2016): 965–972. 26590105 10.1111/jgh.13242

[307] E. Treiner , L. Duban , S. Bahram , et al., “Selection of Evolutionarily Conserved Mucosal‐associated Invariant T Cells by MR1,” Nature 422, no. 6928 (2003): 164–169. 12634786 10.1038/nature01433

[308] E. Martin , E. Treiner , L. Duban , et al., “Stepwise Development of MAIT Cells in Mouse and human,” PLoS Biology 7, no. 3 (2009): e54. 19278296 10.1371/journal.pbio.1000054 PMC2653554

[309] A. Kurioka , A. S. Jahun , R. F. Hannaway , et al., “Shared and Distinct Phenotypes and Functions of Human CD161++ Vα7.2+ T Cell Subsets,” Frontiers in Immunology 8 (2017): 1031. 28912775 10.3389/fimmu.2017.01031 PMC5582200

[310] R. Priya and R. R. Brutkiewicz , “MR1 Tetramer‐Based Artificial APCs Expand MAIT Cells From Human Peripheral Blood That Effectively Kill Glioblastoma Cells,” ImmunoHorizons 5, no. 6 (2021): 500–511. 34172533 10.4049/immunohorizons.2100003

[311] T. Parrot , K. Healy , C. Boulouis , et al., “Expansion of Donor‐unrestricted MAIT Cells With Enhanced Cytolytic Function Suitable for TCR Redirection,” JCI Insight 6, no. 5 (2021): e140074. 33561009 10.1172/jci.insight.140074 PMC8021122

[312] Y. R. Li , Y. Zhou , Y. J. Kim , et al., “Development of Allogeneic HSC‐engineered iNKT Cells for off‐the‐shelf Cancer Immunotherapy,” Cell Reports Medicine 2, no. 11 (2021): 100449. 34841295 10.1016/j.xcrm.2021.100449 PMC8607011

[313] Y. R. Li , Z. S. Dunn , Y. Zhou , D. Lee , and L. Yang , “Development of Stem Cell‐Derived Immune Cells for off‐the‐Shelf Cancer Immunotherapies,” Cells 10, no. 12 (2021): 3497. 34944002 10.3390/cells10123497 PMC8700013

[314] A. Bohineust , M. Tourret , L. Derivry , and S. Caillat‐Zucman , “Mucosal‐associated Invariant T (MAIT) Cells, a New Source of Universal Immune Cells for Chimeric Antigen Receptor (CAR)‐cell Therapy,” Bulletin Du Cancer 108, no. 10 (2021): S92–S95. 34920812 10.1016/j.bulcan.2021.07.003

[315] H. Murthy , M. Iqbal , J. C. Chavez , and M. A. Kharfan‐Dabaja , “Cytokine Release Syndrome: Current Perspectives,” ImmunoTargets and Therapy 8 (2019): 43–52. 31754614 10.2147/ITT.S202015 PMC6825470

[316] L. Ma , T. Dichwalkar , J. Y. H. Chang , et al., “Enhanced CAR‐T Cell Activity Against Solid Tumors by Vaccine Boosting Through the Chimeric Receptor,” Science (New York, NY) 365, no. 6449 (2019): 162–168. 10.1126/science.aav8692 PMC6800571 31296767

[317] Y. Zhou , M. Li , K. Zhou , et al., “Engineering Induced Pluripotent Stem Cells for Cancer Immunotherapy,” Cancers 14, no. 9 (2022): 2266. 35565395 10.3390/cancers14092266 PMC9100203

[318] C. Tastan , E. Karhan , W. Zhou , et al., “Tuning of human MAIT Cell Activation by Commensal Bacteria Species and MR1‐dependent T‐cell Presentation,” Mucosal Immunology 11, no. 6 (2018): 1591–1605. 30115998 10.1038/s41385-018-0072-x PMC6279574

[319] Y. Yasutomi , A. Chiba , K. Haga , et al., “Activated Mucosal‐associated Invariant T Cells Have a Pathogenic Role in a Murine Model of Inflammatory Bowel Disease,” Cellular and Molecular Gastroenterology and Hepatology 13, no. 1 (2022): 81–93. 34461283 10.1016/j.jcmgh.2021.08.018 PMC8593615

[320] A. M. Globig , A. V. Hipp , P. Otto‐Mora , et al., “High‐dimensional Profiling Reveals Tc17 Cell Enrichment in Active Crohn's Disease and Identifies a Potentially Targetable Signature,” Nature Communications 13, no. 1 (2022): 3688. 10.1038/s41467-022-31229-z PMC9237103 35760777

[321] A. Toubal , B. Kiaf , L. Beaudoin , et al., “Mucosal‐associated Invariant T Cells Promote Inflammation and Intestinal Dysbiosis Leading to Metabolic Dysfunction During Obesity,” Nature Communications 11, no. 1 (2020): 3755. 10.1038/s41467-020-17307-0 PMC7381641 32709874

[322] P. Hegde , E. Weiss , V. Paradis , et al., “Mucosal‐associated Invariant T Cells Are a Profibrogenic Immune Cell Population in the Liver,” Nature Communications 9, no. 1 (2018): 2146. 10.1038/s41467-018-04450-y PMC5984626 29858567

[323] A. Riva , V. Patel , A. Kurioka , et al., “Mucosa‐associated Invariant T Cells Link Intestinal Immunity With Antibacterial Immune Defects in Alcoholic Liver Disease,” Gut 67, no. 5 (2018): 918–930. 29097439 10.1136/gutjnl-2017-314458 PMC5890654

[324] T. S. Hinks , X. Zhou , K. J. Staples , et al., “Innate and Adaptive T Cells in Asthmatic Patients: Relationship to Severity and Disease Mechanisms,” The Journal of Allergy and Clinical Immunology 136, no. 2 (2015): 323–333. 25746968 10.1016/j.jaci.2015.01.014 PMC4534770

[325] T. S. Hinks , J. C. Wallington , A. P. Williams , R. Djukanović , K. J. Staples , and T. M. Wilkinson , “Steroid‐induced Deficiency of Mucosal‐associated Invariant T Cells in the Chronic Obstructive Pulmonary Disease Lung. Implications for Nontypeable Haemophilus influenzae Infection,” American Journal of Respiratory and Critical Care Medicine 194, no. 10 (2016): 1208–1218. 27115408 10.1164/rccm.201601-0002OC PMC5114442

[326] J. L. Simpson , J. Daly , K. J. Baines , et al., “Airway Dysbiosis: Haemophilus influenzae and Tropheryma in Poorly Controlled Asthma,” The European Respiratory Journal 47, no. 3 (2016): 792–800. 26647445 10.1183/13993003.00405-2015

[327] X. Wen , S. Nian , G. Wei , et al., “Changes in the Phenotype and Function of Mucosal‐associated Invariant T Cells in Neutrophilic Asthma,” International Immunopharmacology 106 (2022): 108606. 35180624 10.1016/j.intimp.2022.108606

[328] W. Qiu , N. Kang , Y. Wu , et al., “Mucosal Associated Invariant T Cells Were Activated and Polarized toward Th17 in Chronic Obstructive Pulmonary Disease,” Frontiers in Immunology 12 (2021): 640455. 33868270 10.3389/fimmu.2021.640455 PMC8044354

[329] T. Pincikova , T. Parrot , L. Hjelte , et al., “MAIT Cell Counts Are Associated With the Risk of Hospitalization in COPD,” Respiratory Research 23, no. 1 (2022): 127. 35585629 10.1186/s12931-022-02045-2 PMC9114286

[330] R. P. Dickson , J. R. Erb‐Downward , F. J. Martinez , and G. B. Huffnagle , “The Microbiome and the Respiratory Tract,” Annual Review of Physiology 78 (2016): 481–504. 10.1146/annurev-physiol-021115-105238 PMC4751994 26527186

[331] A. Khlaiphuengsin , N. Chuaypen , P. Sodsai , et al., “Successful Direct‐acting Antiviral Therapy Improves Circulating Mucosal‐associated Invariant T Cells in Patients With Chronic HCV Infection,” PLoS ONE 15, no. 12 (2020): e0244112. 33382729 10.1371/journal.pone.0244112 PMC7775079

[332] E. Merlini , M. Cerrone , B. van Wilgenburg , et al., “Association between Impaired Vα7.2+CD161++CD8+ (MAIT) and Vα7.2+CD161‐CD8+ T‐Cell Populations and Gut Dysbiosis in Chronically HIV‐ and/or HCV‐Infected Patients,” Frontiers in Microbiology 10 (2019): 1972. 31555223 10.3389/fmicb.2019.01972 PMC6722213

[333] T. Konuma , C. Kohara , E. Watanabe , et al., “Reconstitution of Circulating Mucosal‐Associated Invariant T Cells After Allogeneic Hematopoietic Cell Transplantation: Its Association With the Riboflavin Synthetic Pathway of Gut Microbiota in Cord Blood Transplant Recipients,” Journal of Immunology (Baltimore, Md: 1950) 204, no. 6 (2020): 1462–1473. 32041784 10.4049/jimmunol.1900681

[334] M. G. Constantinides , V. M. Link , S. Tamoutounour , et al., “MAIT Cells Are Imprinted by the Microbiota in Early Life and Promote Tissue Repair,” Science (New York, NY) 366, no. 6464 (2019): eaax6624. 10.1126/science.aax6624 PMC7603427 31649166

[335] S. Sakai , K. D. Kauffman , S. Oh , C. E. Nelson , C. E. Barry 3rd , and D. L. Barber , “MAIT Cell‐directed Therapy of Mycobacterium Tuberculosis Infection,” Mucosal Immunology 14, no. 1 (2021): 199–208. 32811991 10.1038/s41385-020-0332-4 PMC7790750

[336] X. Yu , Y. Jin , and W. Zhou , “Rifaximin Modulates the Gut Microbiota to Prevent Hepatic Encephalopathy in Liver Cirrhosis without Impacting the Resistome,” Frontiers in Cellular and Infection Microbiology 11 (2021): 761192. 35118004 10.3389/fcimb.2021.761192 PMC8804384

[337] T. S. C. Hinks , E. Marchi , M. Jabeen , et al., “Activation and in Vivo Evolution of the MAIT Cell Transcriptome in Mice and Humans Reveals Tissue Repair Functionality,” Cell Reports 28, no. 12 (2019): 3249–3262. e5. 31533045 10.1016/j.celrep.2019.07.039 PMC6859474

[338] L. J. Howson , W. Awad , A. von Borstel , et al., “Absence of Mucosal‐associated Invariant T Cells in a Person With a Homozygous Point Mutation in MR1,” Science Immunology 5, no. 49 (2020): eabc9492. 32709702 10.1126/sciimmunol.abc9492 PMC8581949

[339] R. J. Dey , B. Dey , M. Harriff , E. T. Canfield , D. M. Lewinsohn , and W. R. Bishai , “Augmentation of the Riboflavin‐Biosynthetic Pathway Enhances Mucosa‐Associated Invariant T (MAIT) Cell Activation and Diminishes Mycobacterium Tuberculosis Virulence,” MBio 13, no. 1 (2021): e0386521. 35164552 10.1128/mbio.03865-21 PMC8844931

[340] R. Rashu , M. Ninkov , C. M. Wardell , et al., “Targeting the MR1‐MAIT Cell Axis Improves Vaccine Efficacy and Affords Protection Against Viral Pathogens,” PLoS Pathogens 19, no. 6 (2023): e1011485. 37384813 10.1371/journal.ppat.1011485 PMC10337970

[341] S. Jalali , C. M. Harpur , A. T. Piers , et al., “A High‐dimensional Cytometry Atlas of Peripheral Blood Over the human Life Span,” Immunology and Cell Biology 100, no. 10 (2022): 805–821. 36218032 10.1111/imcb.12594 PMC9828744

[342] J. E. Ledgerwood , A. D. DeZure , D. A. Stanley , et al., “Chimpanzee Adenovirus Vector Ebola Vaccine,” The New England Journal of Medicine 376, no. 10 (2017): 928–938. 25426834 10.1056/NEJMoa1410863

[343] M. N. Ramasamy , A. M. Minassian , K. J. Ewer , et al., “Safety and Immunogenicity of ChAdOx1 nCoV‐19 Vaccine Administered in a Prime‐boost Regimen in Young and Old Adults (COV002): A Single‐blind, Randomised, Controlled, Phase 2/3 Trial,” Lancet (London, England) 396, no. 10267 (2021): 1979–1993. 33220855 10.1016/S0140-6736(20)32466-1 PMC7674972

[344] N. M. Provine , A. Amini , L. C. Garner , et al., “MAIT Cell Activation Augments adenovirus Vector Vaccine Immunogenicity,” Science (New York, NY) 371, no. 6528 (2021): 521–526. 10.1126/science.aax8819 PMC7610941 33510029

[345] L. J. Howson , G. Napolitani , D. Shepherd , et al., “MAIT Cell Clonal Expansion and TCR Repertoire Shaping in human Volunteers Challenged With Salmonella Paratyphi A,” Nature Communications 9, no. 1 (2018): 253. 10.1038/s41467-017-02540-x PMC5772558 29343684

[346] A. N. Bucsan , N. Rout , T. W. Foreman , S. A. Khader , J. Rengarajan , and D. Kaushal , “Mucosal‐activated Invariant T Cells Do Not Exhibit Significant Lung Recruitment and Proliferation Profiles in Macaques in Response to Infection With Mycobacterium Tuberculosis CDC1551,” Tuberculosis 116 (2019): S11–S18. 10.1016/j.tube.2019.04.006 PMC7050191 31072689

[347] S. Sakai , N. E. Lora , K. D. Kauffman , et al., “Functional Inactivation of Pulmonary MAIT Cells Following 5‐OP‐RU Treatment of Non‐human Primates,” Mucosal Immunology 14, no. 5 (2021): 1055–1066. 34158594 10.1038/s41385-021-00425-3 PMC8217205

[348] J. Jiang , B. Yang , H. An , et al., “Mucosal‐associated Invariant T Cells From Patients With Tuberculosis Exhibit Impaired Immune Response,” The Journal of Infection 72, no. 3 (2016): 338–352. 26724769 10.1016/j.jinf.2015.11.010

[349] J. Jiang , X. Wang , H. An , et al., “Mucosal‐associated Invariant T‐cell Function Is Modulated by Programmed Death‐1 Signaling in Patients With Active Tuberculosis,” American Journal of Respiratory and Critical Care Medicine 190, no. 3 (2014): 329–339. 24977786 10.1164/rccm.201401-0106OC

[350] M. Schmaler , A. Colone , J. Spagnuolo , et al., “Modulation of Bacterial Metabolism by the Microenvironment Controls MAIT Cell Stimulation,” Mucosal Immunology 11, no. 4 (2018): 1060–1070. 29743612 10.1038/s41385-018-0020-9

[351] M. J. Harriff , C. McMurtrey , C. A. Froyd , et al., “MR1 displays the Microbial Metabolome Driving Selective MR1‐restricted T Cell Receptor Usage,” Science Immunology 3, no. 25 (2018): eaao2556. 30006464 10.1126/sciimmunol.aao2556 PMC7085347

[352] Y. S. Chang , S. P. Jalgaonkar , J. D. Middleton , and T. Hai , “Stress‐inducible Gene Atf3 in the Noncancer Host Cells Contributes to Chemotherapy‐exacerbated Breast Cancer Metastasis,” Proceedings of the National Academy of Sciences of the United States of America 114, no. 34 (2017): E7159–e7168. 28784776 10.1073/pnas.1700455114 PMC5576783

[353] I. Keklikoglou , C. Cianciaruso , E. Güç , et al., “Chemotherapy Elicits Pro‐metastatic Extracellular Vesicles in Breast Cancer Models,” Nature Cell Biology 21, no. 2 (2019): 190–202. 30598531 10.1038/s41556-018-0256-3 PMC6525097

[354] J. B. Mitchem , D. J. Brennan , B. L. Knolhoff , et al., “Targeting Tumor‐infiltrating Macrophages Decreases Tumor‐initiating Cells, Relieves Immunosuppression, and Improves Chemotherapeutic Responses,” Cancer Research 73, no. 3 (2013): 1128–1241. 23221383 10.1158/0008-5472.CAN-12-2731 PMC3563931

[355] C. J. Turtle , H. M. Swanson , N. Fujii , E. H. Estey , and S. R. Riddell , “A Distinct Subset of Self‐renewing human Memory CD8+ T Cells Survives Cytotoxic Chemotherapy,” Immunity 31, no. 5 (2009): 834–844. 19879163 10.1016/j.immuni.2009.09.015 PMC2789980

[356] M. Neuenhahn and D. H. Busch , “The Quest for CD8+ Memory Stem Cells,” Immunity 31, no. 5 (2009): 702–704. 19932070 10.1016/j.immuni.2009.10.002

[357] J. R. Fergusson , J. E. Ussher , A. Kurioka , P. Klenerman , and L. J. Walker , “High MDR‐1 Expression by MAIT Cells Confers Resistance to Cytotoxic but Not Immunosuppressive MDR‐1 Substrates,” Clinical and Experimental Immunology 194, no. 2 (2018): 180–191. 30231297 10.1111/cei.13165 PMC6194332

[358] L. H. Butterfield and Y. G. Najjar , “Immunotherapy Combination Approaches: Mechanisms, Biomarkers and Clinical Observations,” Nature Reviews Immunology 24, no. 6 (2023): 399–416. 10.1038/s41577-023-00973-8 PMC11460566 38057451

[359] S. C. Sasson , J. J. Zaunders , K. Nahar , et al., “Mucosal‐associated Invariant T (MAIT) Cells Are Activated in the Gastrointestinal Tissue of Patients With Combination ipilimumab and nivolumab Therapy‐related Colitis in a Pathology Distinct From Ulcerative Colitis,” Clinical and Experimental Immunology 202, no. 3 (2020): 335–352. 32734627 10.1111/cei.13502 PMC7670140

[360] S. De Biasi , L. Gibellini , D. Lo Tartaro , et al., “Circulating Mucosal‐associated Invariant T Cells Identify Patients Responding to anti‐PD‐1 Therapy,” Nature Communications 12, no. 1 (2021): 1669. 10.1038/s41467-021-21928-4 PMC7961017 33723257

[361] K. E. Yost , A. T. Satpathy , D. K. Wells , et al., “Clonal Replacement of Tumor‐specific T Cells Following PD‐1 Blockade,” Nature Medicine 25, no. 8 (2019): 1251–1259. 10.1038/s41591-019-0522-3 PMC6689255 31359002

[362] L. F. Mager , R. Burkhard , N. Pett , et al., “Microbiome‐derived Inosine Modulates Response to Checkpoint Inhibitor Immunotherapy,” Science 369, no. 6510 (2020): 1481–1489. 32792462 10.1126/science.abc3421

[363] R. C. Simpson , E. R. Shanahan , R. A. Scolyer , and G. V. Long , “Towards Modulating the Gut Microbiota to Enhance the Efficacy of Immune‐checkpoint Inhibitors,” Nature Reviews Clinical Oncology 20, no. 10 (2023): 697–715. 10.1038/s41571-023-00803-9 37488231

[364] A. O. Brien , N. Kedia‐Mehta , L. Tobin , et al., “Targeting Mitochondrial Dysfunction in MAIT Cells Limits IL‐17 Production in Obesity,” Cellular & Molecular Immunology 17, no. 11 (2020): 1193–1195. 32107463 10.1038/s41423-020-0375-1 PMC7784973

[365] N. Kedia‐Mehta , M. M. Pisarska , C. Rollings , et al., “The Proliferation of human Mucosal‐associated Invariant T Cells Requires a MYC‐SLC7A5‐glycolysis Metabolic Axis,” Science Signaling 16, no. 781 (2023): eabo2709. 37071733 10.1126/scisignal.abo2709

[366] R. Bergin , D. Kinlen , N. Kedia‐Mehta , et al., “Mucosal‐associated Invariant T Cells Are Associated With Insulin Resistance in Childhood Obesity, and Disrupt Insulin Signalling via IL‐17,” Diabetologia 65, no. 6 (2022): 1012–1017. 35305128 10.1007/s00125-022-05682-w PMC9076704

[367] S. Chandra , G. Ascui , T. Riffelmacher , et al., “Transcriptomes and Metabolism Define Mouse and human MAIT Cell Populations,” Science Immunology 8, no. 89 (2023): eabn8531. 37948512 10.1126/sciimmunol.abn8531 PMC11160507

[368] T. W. Mak , M. Grusdat , G. S. Duncan , et al., “Glutathione Primes T Cell Metabolism for Inflammation,” Immunity 46, no. 4 (2017): 675–689. 28423341 10.1016/j.immuni.2017.03.019

[369] N. A. Gherardin , J. McCluskey , J. Rossjohn , and D. I. Godfrey , “The Diverse Family of MR1‐Restricted T Cells,” The Journal of Immunology 201, no. 10 (2018): 2862–2871. 30397170 10.4049/jimmunol.1801091

[370] J. Peng , B.‐F. Sun , C.‐Y. Chen , et al., “Single‐cell RNA‐seq Highlights Intra‐tumoral Heterogeneity and Malignant Progression in Pancreatic Ductal Adenocarcinoma,” Cell Research 29, no. 9 (2019): 725–738. 31273297 10.1038/s41422-019-0195-y PMC6796938

[371] N. Caronni , F. La Terza , and F. M. Vittoria , “IL‐1β+ Macrophages Fuel Pathogenic Inflammation in Pancreatic Cancer,” Nature 623, no. 7986 (2023): 415–422. 37914939 10.1038/s41586-023-06685-2

[372] S. Kim , G. Leem , J. Choi , et al., “Integrative Analysis of Spatial and Single‐cell Transcriptome Data From human Pancreatic Cancer Reveals an Intermediate Cancer Cell Population Associated With Poor Prognosis,” Genome Medicine 16, no. 1 (2024): 20. 38297291 10.1186/s13073-024-01287-7 PMC10832111

[373] N. G. Steele , E. S. Carpenter , S. B. Kemp , et al., “Multimodal Mapping of the Tumor and Peripheral Blood Immune Landscape in Human Pancreatic Cancer,” Nature Cancer 1, no. 11 (2020): 1097–1112. 34296197 10.1038/s43018-020-00121-4 PMC8294470

[374] E. P. Storrs , P. Chati , A. Usmani , et al., “High‐dimensional Deconstruction of Pancreatic Cancer Identifies Tumor Microenvironmental and Developmental Stemness Features That Predict Survival,” NPJ Precision Oncology 7, no. 1 (2023): 105. 37857854 10.1038/s41698-023-00455-z PMC10587349

[375] G. Werba , D. Weissinger , E. A. Kawaler , et al., “Single‐cell RNA Sequencing Reveals the Effects of Chemotherapy on human Pancreatic Adenocarcinoma and Its Tumor Microenvironment,” Nature Communications 14, no. 1 (2023): 797. 10.1038/s41467-023-36296-4 PMC9925748 36781852

[376] S. Zhang , W. Fang , S. Zhou , et al., “Single Cell Transcriptomic Analyses Implicate an Immunosuppressive Tumor Microenvironment in Pancreatic Cancer Liver Metastasis,” Nature Communications 14, no. 1 (2023): 5123. 10.1038/s41467-023-40727-7 PMC10447466 37612267

[377] J. Zhang , R. Bajari , D. Andric , et al., “The International Cancer Genome Consortium Data Portal,” Nature Biotechnology 37, no. 4 (2019): 367–369. 10.1038/s41587-019-0055-9 30877282

[378] M. J. Goldman , B. Craft , M. Hastie , et al., “Visualizing and Interpreting Cancer Genomics Data via the Xena Platform,” Nature Biotechnology 38, no. 6 (2020): 675–678. 10.1038/s41587-020-0546-8 PMC7386072 32444850

